# Macrophage-to-endothelial cell crosstalk by the cholesterol metabolite 27HC promotes atherosclerosis in male mice

**DOI:** 10.1038/s41467-023-39586-z

**Published:** 2023-07-25

**Authors:** Liming Yu, Lin Xu, Haiyan Chu, Jun Peng, Anastasia Sacharidou, Hsi-hsien Hsieh, Ada Weinstock, Sohaib Khan, Liqian Ma, José Gabriel Barcia Durán, Jeffrey McDonald, Erik R. Nelson, Sunghee Park, Donald P. McDonnell, Kathryn J. Moore, Lily Jun-shen Huang, Edward A. Fisher, Chieko Mineo, Linzhang Huang, Philip W. Shaul

**Affiliations:** 1 Center for Pulmonary and Vascular Biology, Department of Pediatrics, University of Texas Southwestern Medical Center, Dallas, TX 75390 USA; 2 Quantitative Biomedical Research Center and Peter O’Donnell Jr. School of Public Health, University of Texas Southwestern Medical Center, Dallas, TX 75390 USA; 3 Department of Cell Biology, University of Texas Southwestern Medical Center, Dallas, TX 75390 USA; 4 Department of Medicine, New York University School of Medicine, New York, NY 10016 USA; 5 Department of Medicine, University of Chicago School of Medicine, Chicago, IL 60637 USA; 6 University of Cincinnati Cancer Center, Cincinnati, OH 45267 USA; 7 Department of Molecular and Integrative Physiology, University of Illinois at Urbana-Champaign, Urbana, IL 61801 USA; 8 Department of Molecular Genetics, University of Texas Southwestern Medical Center, Dallas, TX 75390 USA; 9 Department of Pharmacology and Cancer Biology, Duke University School of Medicine, Durham, NC 27710 USA; 10 State Key Laboratory of Genetic Engineering, Fudan University, Shanghai, 200433 China; 11 Shanghai Key Laboratory of Metabolic Remodeling and Health, Fudan University, Shanghai, 200433 China; 12 Institute of Metabolism and Integrative Biology, Fudan University, Shanghai, 200433 China

**Keywords:** Hormone receptors, Atherosclerosis

## Abstract

Hypercholesterolemia and vascular inflammation are key interconnected contributors to the pathogenesis of atherosclerosis. How hypercholesterolemia initiates vascular inflammation is poorly understood. Here we show in male mice that hypercholesterolemia-driven endothelial activation, monocyte recruitment and atherosclerotic lesion formation are promoted by a crosstalk between macrophages and endothelial cells mediated by the cholesterol metabolite 27-hydroxycholesterol (27HC). The pro-atherogenic actions of macrophage-derived 27HC require endothelial estrogen receptor alpha (ERα) and disassociation of the cytoplasmic scaffolding protein septin 11 from ERα, leading to extranuclear ERα- and septin 11-dependent activation of NF-κB. Furthermore, pharmacologic inhibition of cyp27a1, which generates 27HC, affords atheroprotection by reducing endothelial activation and monocyte recruitment. These findings demonstrate cell-to-cell communication by 27HC, and identify a major causal linkage between the hypercholesterolemia and vascular inflammation that partner to promote atherosclerosis. Interventions interrupting this linkage may provide the means to blunt vascular inflammation without impairing host defense to combat the risk of atherosclerotic cardiovascular disease that remains despite lipid-lowering therapies.

## Abstract (web summary)

Hypercholesterolemia and vascular inflammation both contribute to the pathogenesis of atherosclerosis, but how hypercholesterolemia initiates vascular inflammation is not fully understood. Here the authors report that crosstalk between macrophages and endothelial cells mediated by the cholesterol metabolite 27-hydroxycholesterol drives vascular inflammation and contributes to atherosclerosis in male mice.

## Introduction

In hypercholesterolemia-induced atherosclerosis, two key processes are the entry of circulating low-density lipoprotein cholesterol (LDL) into the subendothelial space, and endothelial activation prompting the recruitment of monocytes, which differentiate into macrophages to engulf the LDL to become foam cells^1,2^. In prior studies of LDL transport into the artery wall by endothelial cell scavenger receptor class B, type 1 (SR-B1), we found that the LDL entry in and of itself does not influence macrophage accumulation in the arterial intima^3^. Thus, other yet-to-be-defined processes underlie how hypercholesterolemia promotes the endothelial activation and resulting vascular inflammation that partner with dyslipidemia to cause atherosclerosis^4–7^. The present investigation was initiated with studies determining how atherosclerosis is impacted by macrophage sterol 27-hydroxylase (cyp27a1), which catalyzes cholesterol conversion to 27-hydroxycholesterol (27HC) and cholestenoic acid^8,9^. Cerebrotendinous xanthomatosis (CTX) patients, who carry loss-of-function mutations in *cyp27a1*, are at higher risk of developing atherosclerosis despite having circulating lipid levels within normal range^10,11^. Prior work in cell culture has suggested that macrophage cyp27a1 affords atheroprotection by promoting reverse cholesterol transport, mediating cholesterol removal from macrophages through multiple mechanisms^10,12,13^. These include cyp27a1-derived 27HC function as a ligand for liver X receptors (LXR) that upregulates ATP-binding cassette transporter A1 (ABCA1), which mediates cholesterol efflux^14^. However, homozygous versus heterozygous global deletion of *cyp27a1* in mice yields opposing effects on atherosclerosis severity^15^. Seeking to delineate how macrophage cyp27a1 influences atherosclerosis, in the present project the initial hypothesis raised was that macrophage cyp27a1 affords atheroprotection.

Contrary to initial expectations, here we demonstrate in male mice that the selective deletion of *cyp27a1* from macrophages results in a decrease in atherosclerotic lesion severity. We show that the majority of lesion macrophage accumulation in the setting of hypercholesterolemia is surprisingly dependent on macrophage cyp27a1, and that this is related to the promotion of monocyte recruitment prompted by endothelial activation. Experiments with normal versus absent macrophage *cyp27a1* expression concurrent with endothelial cell gene manipulation demonstrate that the pro-atherogenic actions of macrophage-derived 27HC require binding of the oxysterol to endothelial estrogen receptor alpha (ERα). Interrogation of the endothelial ERα interactome reveals that in contrast to 17β-estradiol (E2), 27HC causes the disassociation of the cytoplasmic scaffolding protein septin 11 from ERα. We further identify the ensuing dynamic protein-protein interactions that lead to septin 11-dependent activation of NF-κB. In parallel, NF-kB activation by septin 11 in endothelium is implicated in the disease-promoting macrophage-to-endothelial cell communication by 27HC. Entirely consistent with these mechanisms, pharmacological inhibition of cyp27a1 in macrophages affords atheroprotection by reducing endothelial activation and monocyte recruitment. Collectively, by demonstrating cell-to-cell communication by 27HC in vivo, these findings identify a major causal linkage between the hypercholesterolemia and the vascular inflammation that partner to promote atherosclerosis. Interventions that disrupt this linkage may provide a means to blunt vascular inflammation without impairing host defense to combat the substantial risk of atherosclerotic cardiovascular disease that remains despite lipid-lowering therapies.

## Results

### Macrophage-derived 27HC promotes atherosclerosis

**Fig. 1:**
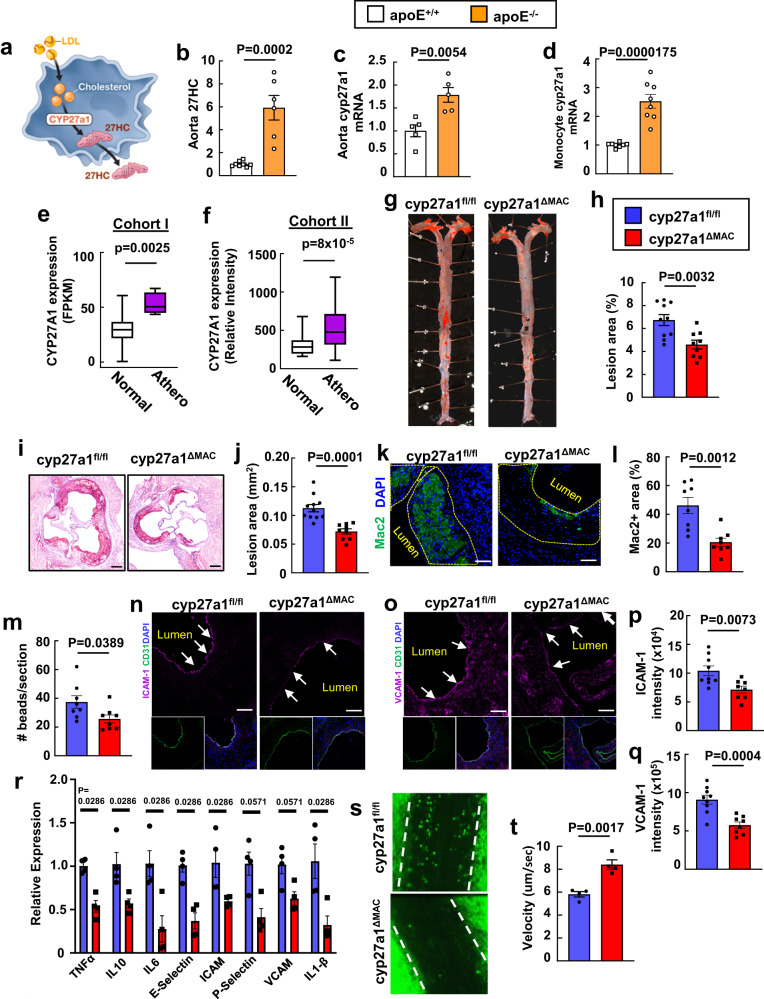
Macrophage-derived 27HC promote atherosclerosis by increasing vascular inflammation. **a** Schematic of cyp27a1 conversion of cholesterol to 27HC in a macrophage. 27HC content (**b**, *n* = 8, 6) and *cyp27a1* expression (**c**, n = 5) were evaluated in aortas from apoE^+/+^ and apoE^−/−^ mice following receipt of standard chow or atherogenic diet, respectively. **d** C*yp27a1* expression in circulating monocytes (n = 8) from apoE^+/+^ and apoE^-/-^ mice administered diets as in (**b**). **e**, **f** *Cyp27a1* expression was assessed in human atherosclerotic versus control arteries in two independent cohorts. Cohorts I (**e**) and II (**f**) contained n = 4 and 198, and 32 and 32 samples respectively. FPKM is fragments per kilobase of transcript per million mapped reads. Box-and-whisker plots are provided; central line denotes the median value, edges represent the upper and lower quartiles, and whiskers indicate minimum and maximum values. In (**b**), (**c**) and (**d**) values are expressed relative to findings in apoE^+/+^. **g–r** Atherosclerosis and related parameters in apoE^-/-^ background cyp27a1^fl/fl^ and cyp27a1^ΔMAC^ mice. Representative lipid-stained en face images of aortas (**g**) and lesion areas (**h**; percent of total surface area, n = 10 and 9). Representative lipid and haematoxylin-stained aortic root sections (**i**), and lesion areas (**j**, n = 11 and 9). Representative immunohistochemistry images of Mac2 staining in aortic root (**k**; nuclei DAPI stained) and quantification of Mac2 positive staining expressed per unit lesion area (**l**; n = 8). **m** Monocyte recruitment to atherosclerotic lesions was evaluated by YG bead incorporation into circulating monocytes and quantification of beads per aortic root section 24 h later. N = 8. Immunohistochemical determinations of endothelial ICAM-1 (**n**, **p**) and VCAM-1 abundance (**o**, **q**) with representative images (**n**, **o**) and quantification (**p**, **q**, n = 9 and 8). Arrows indicate endothelium. **r** Inflammatory gene expression in the aorta (n = 4). **s** Representative still images of leukocyte-endothelial cell adhesion in apoE^-/-^ background cyp27a1^fl/fl^ and cyp27a1^∆MAC^ mice. **t** Summary data for leukocyte velocity (n = 4). Scale bar equals 100 um (**i**) or 50 um (**k**, **n**, **o**). Except in (**e**) and (**f)**, data are mean ± SEM. P values by two-sided Student’s t-test (**b**–**f**, **h**, **j**, **l**, **m**, **p**, **q**, **t**) or two-sided Mann-Whitney U-test (**r**) are shown. Source data are provided as a Source Data file.

To initiate the inquiry into how cyp27a1 production of 27HC in macrophages (Fig. 1a) influences atherosclerosis, we first determined that both 27HC content and *cyp27a1* expression are increased in the aortas of hypercholesterolemic apoE^-/-^ mice compared to those of normocholesterolemic standard chow-fed wild-type mice (Fig. 1b, c, Supplementary Fig. 1a). Our previously-reported single-cell RNA-sequencing (RNAseq) of plaque immune cells from mice with hypercholesterolemia or improved cholesterolemia was then queried^16–18^. This revealed that macrophages and monocytes are the immune cell types expressing the most cyp27a1 (Supplementary Fig. 1b), and that cyp27a1 expression is increased in macrophages and not in other immune cells in the setting of greater hypercholesterolemia (Supplementary Fig. 1c, d). *Cyp27a1* gene expression in circulating monocytes is also upregulated in the setting of hypercholesterolemia (Fig. 1d, Supplementary Fig. 2). These observations mirror prior findings for 27HC abundance and immunostaining for cyp27a1 in human atherosclerosis^19,20^, which we now strengthen by determining that cyp27a1 transcript level is greater in atherosclerotic versus normal arteries in two independent human cohorts (Fig. 1e, f). Single-cell RNAseq studies of human atherosclerotic lesions reveal that cyp27a1 is expressed primarily in foamy macrophages, and not in other immune or non-immune cells^21,22^. Thus, mice and humans have a similar profile for cyp27a1 transcript abundance in immune cells relevant to atherosclerosis pathogenesis, with expression primarily demonstrable in macrophages.

The impact of macrophage cyp27a1 on the initiating events in plaque formation and early atherosclerosis progression was then determined. Floxed control mice (cyp27a1^fl/fl^) and mice deficient in the enzyme in macrophages (cyp27a1^∆MAC^) were placed on apoE^-/-^ background and fed an atherogenic diet for 8 weeks following weaning (Supplementary Fig. 3a–d). Whereas plasma total cholesterol, triglyceride and HDL levels, and lipoprotein profiles were similar in the two genotype groups (Supplementary Table 1, Supplementary Fig. 4a), serum 27HC was 627 ± 43 and 446 ± 32 ng/ml in cyp27a1^fl/fl^ and cyp27a1^ΔMAC^ mice, respectively (*n* = 10 and 9, *p* = 0.0013). Contrary to prior in vitro findings indicating that macrophage 27HC production promotes cholesterol efflux and thus may be atheroprotective^10,12,13^, cyp27a1^∆MAC^ mice had less atherosclerosis than cyp27a1^fl/fl^ controls (Fig. 1g–j). The expression of LXR target genes in macrophages including ATP-binding cassette A1 (ABCA1) (Supplementary Fig. 4f) was not altered with *cyp27a1* silencing, providing additional evidence that macrophage cyp27a1 does not influence reverse cholesterol transport. Instead, in the absence of any impact on circulating monocyte number (Supplementary Fig. 4g, h), the macrophage enzyme causes substantial promotion of vascular inflammation, with loss-of-function in cyp27a1^∆MAC^ mice yielding a 55% decline in lesion macrophage abundance indicated by Mac2 area per unit lesion area (Fig. 1k, l). Therefore, to our initial surprise, macrophage cyp27a1 is not atheroprotective, and instead it promotes the disorder and is responsible for the majority of macrophage accumulation in atherosclerotic lesions.

To explain macrophage cyp27a1-related changes in lesion macrophage abundance, lesion necrotic area and apoptosis were evaluated, and they were similar in cyp27a1^fl/fl^ and cyp27a1^ΔMAC^ mice (Supplementary Fig. 5a–d). Monocyte recruitment, which is the major driver of lesion macrophage accumulation^23,24^, was quantified by marking circulating monocytes by their uptake of latex beads (Supplementary Fig. 6a, b). Recruitment was decreased with macrophage *cyp27a1* silencing (Fig. 1m), and this was likely explained by declines in both endothelial cell ICAM-1 and VCAM-1 expression (Fig. 1n–q). In parallel, a number of inflammatory genes in the aorta were downregulated in cyp27a1^ΔMAC^ mice compared to cyp27a1^fl/fl^ mice (Fig. 1r). In addition, intravital microscopy of the mesenteric microcirculation directly demonstrated that the deletion of *cyp27a1* from macrophages in apoE^-/-^ mice raises the velocity of adherent leukocytes (Fig. 1s, t, Supplemental Movies 1 and 2), indicating a decrease in leukocyte-endothelial cell adhesion. Thus, in the setting of hypercholesterolemia, endothelial cell adhesion molecule expression and leukocyte-endothelial cell adhesion are promoted by macrophage cyp27a1. Contrary to the initial hypothesis, these overall findings reveal that macrophage-derived 27HC is proatherogenic, driving the majority of lesion macrophage abundance by stimulating endothelial activation.

### Macrophage-derived 27HC cross-talk to endothelial ERα

**Fig. 2:**
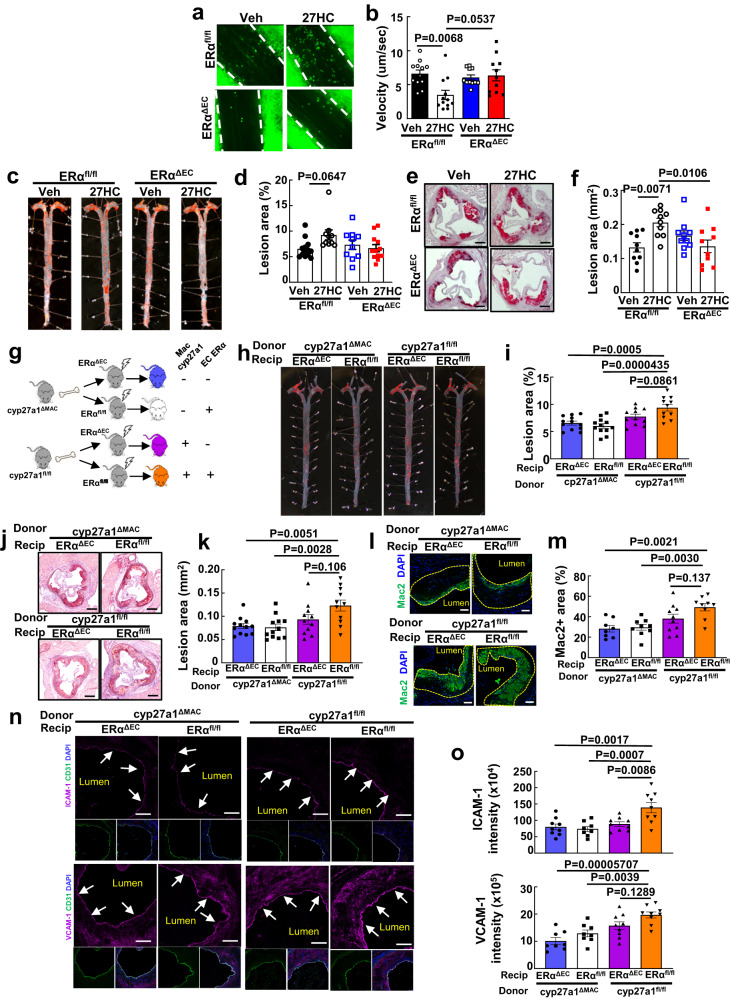
Macrophage-derived 27HC drives vascular inflammation and promotes atherosclerosis via endothelial ERα. **a** Representative still images of leukocyte-endothelial cell adhesion evaluated by intravital microscopy in ERα^fl/fl^ and ERα^∆EC^ mice injected with vehicle versus 27HC (20 mg/kg BW) daily for 3 days. **b** Summary data for leukocyte velocity in (**a**), n = 11-12. **c**–**f** ApoE^-/-^ background ERα^fl/fl^ and ERα^ΔEC^ mice were placed on an atherogenic diet for 8 weeks and administered vehicle versus 27HC, and samples were obtained. Representative lipid-stained en face images of aortas (**c**), and lesion areas (**d**, percent of total surface area). N = 13 and 10 for ERα^fl/fl^ given vehicle versus 27HC, respectively, and n = 10 and 12 for ERα^ΔEC^ given vehicle versus 27HC, respectively. Representative lipid and haematoxylin-stained aortic root sections (**e**), and lesion areas (**f**, n = 10). **g**–**o**, Atherosclerosis and related parameters in bone marrow transplant experiments performed employing apoE^-/-^ background cyp27a1^fl/fl^ versus cyp27a1^ΔMAC^ donors and apoE^-/-^ background ERα^fl/fl^ versus ERα^ΔEC^ recipients. **g** Schematic of donors and recipients. Representative lipid-stained en face images of aortas (**h**) and lesion areas (**i**, n = 11-12). Representative lipid and haematoxylin-stained aortic root sections (**j**), and lesion areas (**k**, n = 11-12). Representative immunohistochemistry images of Mac2 staining in aortic root (**l**; nuclei DAPI stained) and quantification of Mac2 positive staining (**m**; expressed per unit lesion area, n = 8−10). Immunohistochemical determinations of endothelial ICAM-1 and VCAM-1 abundance with representative images (**n**) and quantification (**o**, n = 8-9). Arrows indicate endothelium. Scale bar equals 100 um (**e**, **j**) or 50 um (**l**, **n**). Data are mean ± SEM. In (**b**) and (**d**), P values shown are by Krusakal-Wallis with Dunn’s post hoc testing. In all other graphs (**f**, **i**, **k**, **m**, **o**), P values shown are by ANOVA with Tukey’s post-hoc testing. Source data are provided as a Source Data file.

The molecular mechanism by which macrophage cyp27a1-derived 27HC increases endothelial activation was then pursued recognizing our prior discovery that the 27HC generated from cholesterol by cyp27a1 is an estrogen receptor (ER) α ligand^25,26^. We first determined if ERα is a target of 27HC in endothelial cells by which the oxysterol promotes endothelial activation in vivo. Intravital microscopy demonstrated that whereas 27HC administration decreases leukocyte velocity in control floxed ERα mice (ERα^fl/fl^), the proadhesive action of 27HC is entirely absent in mice deficient in ERα selectively in endothelial cells (ERα^ΔEC^) (Fig. 2a, b, Supplementary Fig. 3e, Supplemental Movies 3–6). The impact of 27HC supplementation on atherogenesis was also evaluated in ERα^fl/fl^ and ERα^ΔEC^ mice. Resulting serum 27HC levels were 883 ± 63, 1774 ± 224*, 698 ± 32 and 2151 ± 256** ng/ml in ERα^fl/fl^ administered vehicle versus 27HC and in ERα^ΔEC^ given vehicle versus 27HC, *N* = 13, 14, 10 and 12, respectively (*p = 0.0012 and **P = 0.00002 versus vehicle); with its administration, serum 27HC levels rose similarly in ERα^fl/fl^ and ERα^ΔEC^ mice. Without altering circulating total cholesterol, triglycerides or HDL, or lipid profile (Supplementary Table 1 and Supplementary Fig. 4b), the provision of exogenous 27HC increased early plaque formation and early atherosclerosis progression in apoE^−/−^ mice expressing endothelial ERα, but not in apoE^-/-^ lacking endothelial ERα (Fig.  2c–f). Thus, endothelial cells, and endothelial ERα specifically, are the relevant targets of 27HC in vivo by which the oxysterol increases endothelial activation and atherosclerosis severity.

Based on these observations, to directly determine if macrophage cyp27a1-derived 27HC mediates proatherogenic crosstalk between macrophages and endothelial cells, bone marrow transplant experiments were performed employing donors with normal versus silenced macrophage *cyp27a1* expression and recipients with normal versus deficient endothelial ERα abundance (Fig. 2g, Supplementary Fig. 7a). Atherosclerotic lesion quantification in both en face preparations of aorta and aortic root sections revealed that compared to mice deficient in both macrophage cyp27a1 and endothelial *ERα*, the provision of either the receptor in endothelium or the enzyme in macrophages alone did not affect lesion severity (Fig. 2h–k). It was only with the introduction of macrophage cyp27a1 in mice expressing endothelial ERα that lesion size increased beyond that observed in the absence of macrophage cyp27a1 and endothelial ERα. In parallel, neither endothelial ERα nor macrophage cyp27a1 provision alone influenced lesion macrophage content, and it was only in the presence of endothelial ERα that macrophage cyp27a1 enhanced macrophage accumulation (Fig. 2l, m). The basis by which macrophage-to-endothelial cell crosstalk by 27HC participates in atherosclerosis development was further delineated with the finding that increases in endothelial cell ICAM-1 and VCAM-1 expression above those found in the absence of macrophage *cyp27a1* and endothelial *ERα* were only promoted by macrophage cyp27a1 in mice expressing the receptor in endothelium (Fig. 2n, o). It should be noted that the capacity to demonstrate this proatherogenic action of endothelial ERα by loss-of-function and return of function may have been tempered by the concomitant absence versus presence of atheroprotective mechanisms mediated by the receptor^27^. Despite this possible limitation, these findings reveal that in the setting of hypercholesterolemia, macrophage-derived 27HC partners with endothelial ERα to promote endothelial activation and vascular inflammation, particularly the bulk of intimal macrophage accumulation, and consequently atherosclerotic lesion development.

### Septin 11 mediates endothelial activation by 27HC

**Fig. 3:**
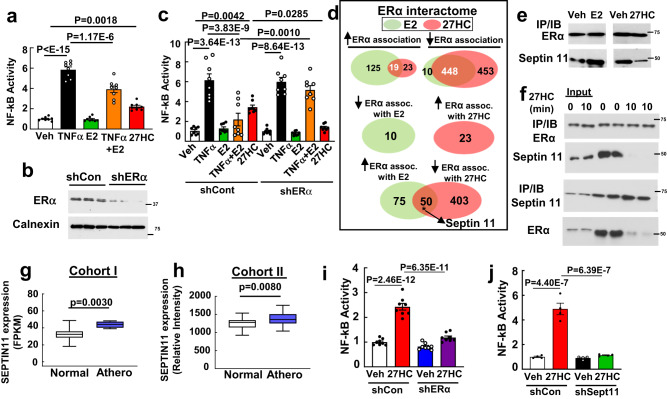
Septin 11 mediates 27HC activation of endothelial inflammation via ERα. **a**–**c** 27HC activation of NF-κB activity in HAEC via ERα. **a**, NF-κB luciferase activity was measured in HAEC treated with TNFα (10 ng/ml), E2 (10^−8^M),TNFα plus E2, or 27HC (20uM) for 6 h (n = 8). **b**, **c** Following transduction with control shRNA versus shRNA targeting ERα (**b**, findings for 3 samples per group are shown), the experiments described in (**a**) were performed (**c**, n = 8). **d** Venn diagrams of proteins identified by LC-MS/MS to have increased or decreased association with ERα upon E2 versus 27HC treatment in HAEC. Diagrams display intersects for common directional changes with the two ligands (upper panel), and opposing directional changes with the two ligands (lower two panels). **e** Co-immunoprecipitation (IP) of endogenous ERα and septin 11 in HAEC treated with vehicle versus E2 (left panel), or with vehicle versus 27HC (right panel) for 30 min. **f** HAEC were treated with 27HC, and IP of endogenous ERα was performed and immunoblotting was done for ERα and septin 11 (upper panel), or IP of septin 11 was performed followed by immunoblotting. *Septin 11* expression in human atherosclerotic versus control arteries in two independent cohorts. Cohorts I (**g**) and II (**h**), which were analyzed by RNAseq and microarray, respectively, contained n = 4 and 201, and 32 and 32 samples respectively. FPKM is fragments per kilobase of transcript per million mapped reads. Box-and-whisker plots are provided in which the central line denotes the median value, the edges represent the upper and lower quartiles, and whiskers indicate the minimum and maximum values. Effect of vehicle versus 27HC on NF-κB activity in HAEC expressing or lacking ERα (**i**, n = 8) or septin 11 (**j**, n = 4). In (**a**), (**c**), (**i**) and (**j**), NF-κB activity is expressed relative to values with vehicle treatment. Data are mean ± SEM. In (**a**) and (**c**), P values shown are by Krusakal-Wallis with Dunn’s post hoc testing, and in (**g**) and (**h**) two-sided Student’s t tests were used. In (**i**) and (**j**), P values by ANOVA with Tukey’s post-hoc testing are shown. The findings in (**e**) and (**f**) were confirmed in two independent experiments. Source data are provided as a Source Data file.

To further determine how 27HC activates inflammatory processes in endothelial cells, we leveraged the opposing actions of 17β-estradiol (E2) and 27HC, which inhibit and promote NF-κB activity, respectively, in human aortic endothelial cells (HAEC) (Fig. 3a). Demonstrating that the diametric effects of the two ligands are both mediated by ERα (Fig. 3b, c), we overexpressed ERα in HAEC and then compared the ERα interactomes with E2 versus 27HC treatment by liquid chromatography and tandem mass spectrometry (LC-MS/MS). Unique sets of proteins displayed increased ERα association or disassociation in response to E2 versus 27HC (Fig. 3d, Supplementary Tables 2 and 3). One of the proteins identified to have contrasting interaction with ERα bound by E2 versus 27HC was septin 11, which is a cytoplasmic scaffolding protein^28^. LC-MS/MS revealed that in response to E2, septin 11 is recruited to ERα, and in contrast, septin 11 disassociates from 27HC-bound ERα (Fig. 3d, lower panel). Co-immunoprecipitation (co-IP) studies in HAEC expressing endogenous levels of ERα and septin 11 verified the LC-MS/MS findings and displayed the dynamic nature of the response to 27HC (Fig. 3e, f). Interestingly, we found that septin 11 transcript levels are greater in human atherosclerotic arteries compared to normal arteries in two independent human cohorts (Fig. 3g, h). shRNA silencing of ERα and septin 11 in HAEC revealed identical prevention of 27HC activation of NF-κB activation (Fig. 3i, j) and monocyte-endothelial cell adhesion (Supplementary Fig. 8a, b). Thus, septin 11 is critically involved in the unique proinflammatory actions of the 27HC-ERα tandem in endothelial cells.

**Fig. 4:**
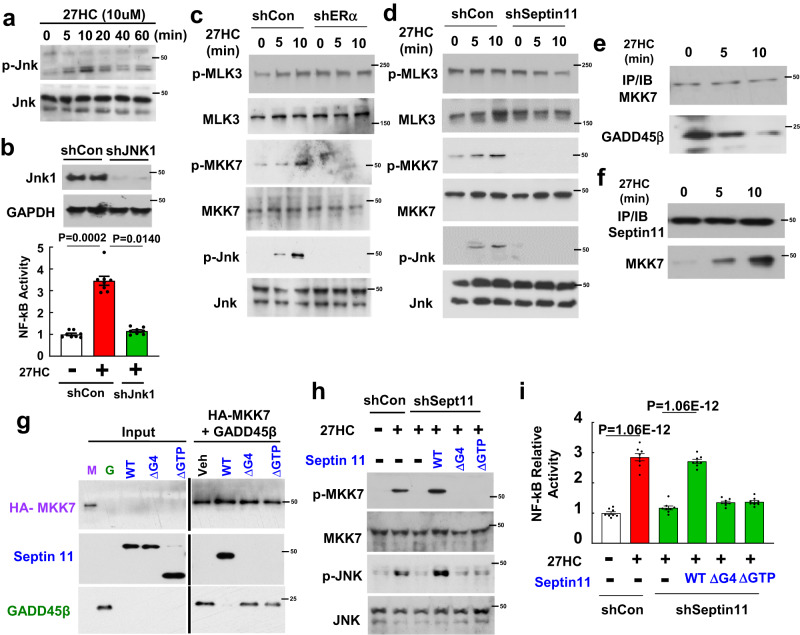
Septin 11 mediates endothelial activation by the 27HC-ERα tandem by disrupting the interaction between MKK7 and its inhibitory protein GADD45β and thereby activating Jnk. **a** HAEC were treated with 27HC and immunoblotting was performed to detect Jnk Thr183/Tyr185 phosphorylation and total Jnk. **b** Following transfection of HAEC with control shRNA or shRNA targeting Jnk1 (upper panel, findings for 2 samples/group are shown), the effects of vehicle versus 27HC on NF-κB activity were determined (lower panel, n = 8). Following transduction of HAEC with control shRNA or shRNA targeting ERα (**c**) or septin 11 (**d**), HAEC were treated with 27HC, and immunoblotting was performed to detect activating phosphorylation of MLK3, MKK7 and Jnk, and total MLK3, MKK7 and Jnk. HAEC were treated with 27HC for 0 to 10 min, MKK7 was IP’d and immunoblotting was performed for MKK7 and GADD45β (**e**), or septin 11 was IP’d and immunoblotting was done for MKK7 and septin 11 (**f**). **g** In pull-down experiments a complex between HA-tagged bound MKK7 and GADD45β was formed. Wild-type septin 11 (WT) or septin 11 deletion mutants lacking AA184-192 (septin 11-∆G4) or AA38-304 (septin 11-∆GTP) were added, and protein associations were evaluated by immunoblotting. **h**, **i** Requirement for septin 11 interaction with MKK7 in 27HC action was evaluated in HAEC in which endogenous *septin 11* was knocked down by shRNA, and reconstitution was performed with either wild-type septin 11, septin 11-∆G4, or septin 11-∆GTP. (**h**) Cells were treated with vehicle or 27HC, and immunoblotting was performed to detect activating phosphorylation of MKK7 and Jnk, and total MKK7 and Jnk. (**i**) In the same study groups as in (**h**), NF-κB activity was evaluated in cells treated with vehicle or 27HC. n = 8. In (**b**) and (**i**), NF-κB activity is expressed relative to values with vehicle treatment. Data are mean ± SEM. In (**b**) and (**i**), P values by ANOVA with Tukey’s post-hoc testing are shown. The findings in (**a**) and (**c**)–(**h**) were confirmed in two independent experiments. Source data are provided as a Source Data file.

Recognizing the cytoplasmic localization of septin 11, how it participates in NF-κB activation by 27HC was then determined in studies of kinase signaling, which is controlled by a subpopulation of extra-nuclear ERα in a variety of cell types including endothelium^29, 30^. Jnk was implicated in 27HC action by observing that the oxysterol induces an activating phosphorylation of Jnk-Thr183/Tyr185 in endothelial cells (Fig. 4a), and that Jnk1 silencing fully prevents 27HC activation of NF-κB (Fig. 4b). In parallel with the increase in Jnk-Thr183/Tyr185 phosphorylation, the Jnk kinase kinase MKK7^31^ was activated by 27HC (Fig. 4c, d); in contrast, MLK3, the kinase for MKK7^31^, was not activated, and 27HC activation of both MKK7 and Jnk was equally negated by silencing ERα or septin 11. To then determine how septin 11 modulates the Jnk signaling pathway in response to 27HC, using co-IP we assessed whether 27HC alters the interaction between MKK7 and its inhibitory protein GADD45β^32^. 27HC caused a decrease in MKK7-GADD45β interaction (Fig. 4e), and there was concomitant recruitment of septin 11 to MKK7 (Fig. 4f). Pull-down experiments then demonstrated direct interaction between septin 11 and MKK7 (Supplementary Fig. 9b, c). To determine if the interaction is required in 27HC modulation of Jnk signaling and resulting activation of NF-kB, mutant forms of septin 11 were sought that lack interaction with MKK7. Since GTP binding induces conformational changes in septins that influence their protein-protein interactions^33^, we introduced substitutions or deletions within the highly-conserved GTP-binding domain, which includes three common GTP-binding motifs designated G1, G3 and G4 (Supplementary Fig. 9a)^34^. Deletion of the G4 (xKxD) motif (∆G4, AA184-192) or the entire G-interface (∆GTP, AA38-304) yielded stable proteins that did not interact with MKK7 (Supplementary Fig. 9b, c). In further pull-down experiments (Fig. 4g), following the formation of a complex between MKK7 and GADD45β, the addition of wild-type septin 11 caused disassociation of MKK7 and GADD45β, and septin 11 binding to MKK7 was observed. In contrast, neither septin 11-∆G4 or septin 11-∆GTP, which do not interact with MKK7, caused GADD45β disassociation from MKK7. To then determine if septin 11 interaction with MKK7 is required for 27HC action in intact endothelial cells, endogenous septin 11 was deleted and reconstitution was performed with either wild-type septin 11, septin 11-∆G4, or septin 11-∆GTP (Supplementary Fig. 9d). Upon 27HC treatment, cells harboring wild-type septin 11 displayed recruitment of the protein to MKK7 (Supplementary Fig. 9e), disassociation of GADD45β from MKK7 (Supplementary Fig. 9f), MKK7 and Jnk activating phosphorylation (Fig. 4h), and NF-κB activation (Fig. 4i). In contrast, these responses were absent in cells expressing septin 11-∆G4 or septin 11-∆GTP (Supplementary Fig. 9e, f, Fig. 4h, i). These observations reveal that through the modulation of the protein-protein interaction between MKK7 and GADD45β that governs the control of NF-kB activity by Jnk, septin 11 uniquely mediates endothelial activation by 27HC binding to ERα.

### Macrophage-derived 27HC cross-talk to endothelial septin 11

**Fig. 5:**
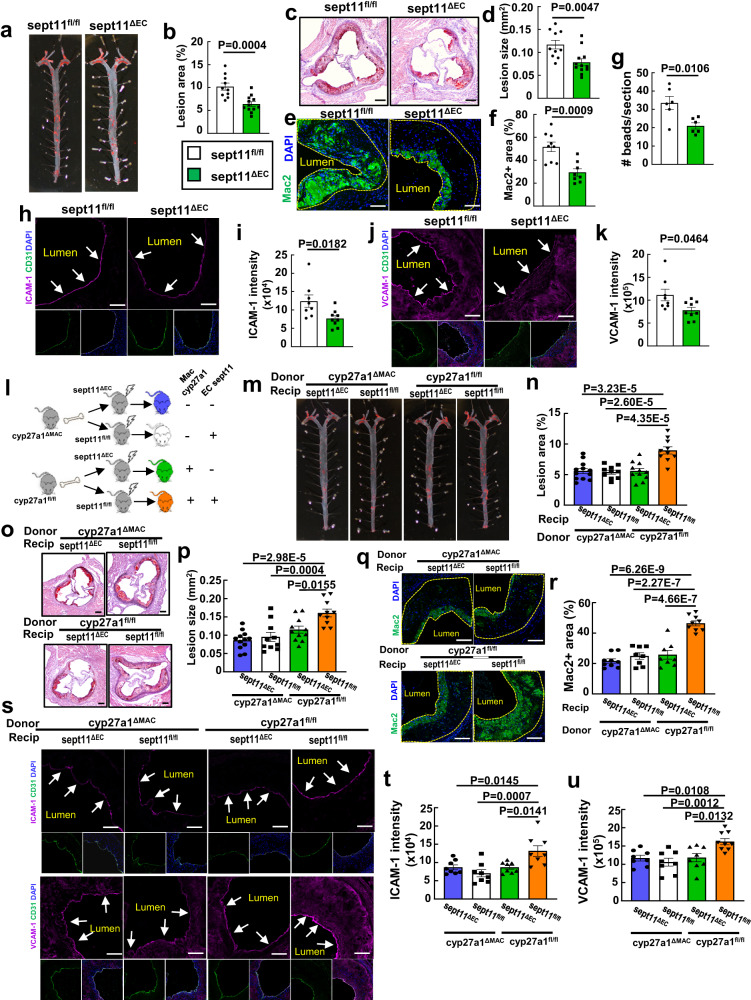
Macrophage-derived 27HC drives vascular inflammation and promotes atherosclerosis via endothelial septin 11. **a-k** Atherosclerosis and related parameters were assessed in apoE^-/-^ background sept1^fl/fl^ and sept11^ΔEC^ mice placed on an atherogenic diet for 8 weeks post-weaning. **a**, **b** Representative lipid-stained en face images of aortas (**a**) and lesion areas (**b**; percent of total surface area, n = 10 and 12). Representative lipid and haematoxylin-stained aortic root sections (**c**) and lesion areas (**d**, n = 10 and 12). Representative immunohistochemistry images of Mac2 staining in aortic root (**e**; nuclei DAPI stained) and quantification of Mac2 positive staining (**f**; expressed per unit lesion area, n = 8-9). **g**, Monocyte recruitment was evaluated by YG bead incorporation into circulating monocytes and quantification of beads per aortic root section 24 h later. The quantitation was corrected for monocyte labeling efficiency. N = 6. Immunohistochemical determinations of endothelial ICAM-1 (**h**, **i**) and VCAM-1 abundance (**j**, **k**) with representative images (**h**, **j**) and quantification (**i**, **k**, n = 8-9). Arrows indicate endothelium. **l**–**u** Atherosclerosis and related parameters in bone marrow transplant experiments performed employing apoE^-/-^ background cyp27a1^fl/fl^ versus cyp27a1^ΔMAC^ donors and apoE^-/-^ background sept11^fl/fl^ versus sept11^ΔEC^ recipients. **l** Schematic of donors and recipients. Representative lipid-stained en face images of aortas (**m**) and lesion areas (**n**, n = 10-12). Representative lipid and haematoxylin-stained aortic root sections (**o**), and lesion areas (**p**, n = 10-12). Representative immunohistochemistry images of Mac2 staining in aortic root (**q**) and quantification of Mac2 positive staining (**r**, n = 8-9). **s–u** Immunohistochemical determinations of endothelial ICAM-1 and VCAM-1 abundance with representative images (**s**) and quantification (**t, u**, n = 8-9). Scale bar equals 100um (**c**, **o**) or 50 um (**e**, **h**, **j**, **q**, **s**). Data are mean ± SEM, **P** values by two-sided Student’s t test (**b**, **d**, **f**, **g**, and **i**), by two-sided Mann-Whitney (**k**), or by ANOVA with Tukey’s post-hoc testing (**n**, **p**, **r**, **t** and **u**) are shown. Source data are provided as a Source Data file.

Having discovered in cell culture that septin 11 regulates endothelial activation by 27HC, its impact on atherosclerosis was determined in control floxed *septin 11* mice (sept11^fl/fl^) and mice deficient in *septin 11* in endothelial cells (sept11^∆EC^) on apoE^-/-^ background (Supplementary Fig. 3f–i). Atherosclerotic lesion severity was decreased by endothelial *septin 11* silencing in the absence of any changes in circulating lipids (Fig. 5a–d, Supplementary Table 1, Supplementary Fig. 4c). The decline in atherosclerosis severity with endothelial *septin 11* deletion was related to a decrease in lesion macrophage abundance (Fig. 5e, f), which was not due to a change in lesion necrosis or apoptosis, and instead to an attenuation in monocyte recruitment (Supplementary Figs. 5e–h, 6c, Fig. 5g). The decrease in monocyte recruitment in mice lacking endothelial cell septin 11 is likely explained by an attenuation in endothelial cell ICAM-1 and VCAM-1 expression (Fig. 5h–k), which is consistent with the identified role of septin 11 in endothelial NF-kB regulation.

To determine if septin 11 in the endothelium is required for the pro-atherogenic actions of macrophage-derived 27HC, bone marrow with normal versus silenced myeloid cell cyp27a1 expression was transplanted into mice with normal versus deficient endothelial *septin 11* (Fig. 5l, Supplementary Fig. 7b). Evaluations of atherosclerotic lesion size demonstrated that relative to mice deficient in both macrophage *cyp27a1* and endothelial *septin 11*, the provision of either the endothelial septin 11 protein or the macrophage enzyme alone did not advance atherosclerosis (Fig. 5m–p). It was only with the introduction of macrophage cyp27a1 in mice expressing endothelial septin 11 that atherosclerotic lesion size increased beyond that observed in the absence of both macrophage cyp27a1 and endothelial septin 11. In parallel, endothelial septin 11 expressing mice provided macrophage cyp27a1-positive bone marrow were the only group in which there is an augumention in lesion macrophage abundance (Fig. 5q, r), and it was only in the presence of endothelial septin 11 that macrophage cyp27a1 enhanced endothelial cell ICAM-1 and VCAM-1 expression (Fig. 5s–u). These observations demonstrate that endothelial septin 11 mediates the disease-promoting actions of macrophage-derived 27HC. Phenocopying the outcome of the bone marrow transplant experiments in recipients in which endothelial ERα was manipulated (Fig. 2h–o), they additionally strengthen the evidence of a key role for 27HC-mediated crosstalk between macrophages and endothelial cells in atherosclerosis development.

### Cyp27a1 inhibition is atheroprotective

**Fig. 6:**
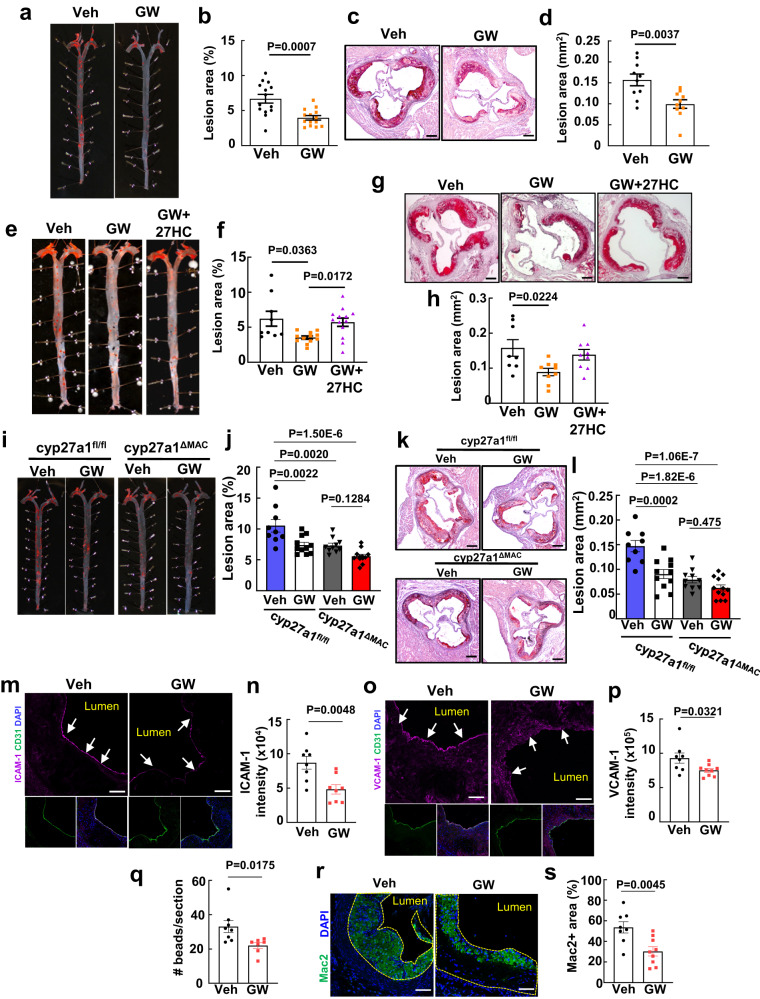
Cyp27a1 inhibition is atheroprotective. **a–d** ApoE^-/-^ mice were placed on an atherogenic diet for 8 weeks, receiving vehicle or the cyp27a1 inhibitor GW273297X (GW) during the last 4 weeks, and atherosclerotic lesions were evaluated. Representative lipid-stained en face images of aortas (**a**) and lesion areas (**b**; percent of total surface area, n = 14). Representative lipid and haematoxylin-stained aortic root sections (**c**), and lesion areas (**d**, n = 10). **e**–**h** In parallel experiments, apoE^-/-^ mice received vehicle, cyp27a1 inhibitor GW, or GW plus exogenous 27HC during the last 4 weeks of an 8 week period on an atherogenic diet. Representative lipid-stained en face images of aortas (**e**) and lesion areas (**f**, n = 9-13). Representative lipid and haematoxylin-stained aortic root sections (**g**), and lesion areas (**h**, n = 8-9). **i**–**l** The effect of vehicle versus GW was determined in apoE^-/-^ background cyp27a1^fl/fl^ and cyp27a1^ΔMAC^ mice. Representative lipid-stained en face images of aortas (**i**) and lesion areas (**j**, n = 9-12). Representative lipid and haematoxylin-stained aortic root sections (**k**), and lesion areas (**l**, n = 9-12). Effect of cyp27a1 inhibitor on abundance of endothelial ICAM-1 (**m, n**) and VCAM-1 abundance (**o**, **p**) with representative images (**m**, **o**) and quantification (**n**, **p**, n = 8-9). **q** Effect of cyp27a1 inhibitor on monocyte recruitment, evaluated by YG bead incorporation into circulating monocytes and quantification of beads per aortic root section 24 h later. The quantitation was corrected for monocyte labeling efficiency. N = 7-8. **r**, **s** Effect of cyp27a1 inhibitor on lesion macrophage accumulation. Representative immunohistochemistry images of Mac2 staining in aortic root (**r**; nuclei DAPI stained) and quantification of Mac2 positive staining (**s**; expressed per unit lesion area, n = 8-9). Scale bar equals 100 um (**c**, **g**, **k**) or 50 um (**m**, **o**, **r**). Data are mean ± SEM, *P* values by two-sided Student’s t test (**b**, **d**, **n**, **p**, **q** and **s**), by Krusakal-Wallis with Dunn’s post hoc testing (**f**), or by ANOVA with Tukey post-hoc testing (**h**, **j** and **l**) are shown. Source data are provided as a Source Data file.

Having revealed that cyp27a1 hydroxylation of cholesterol to form 27HC in macrophages is proatherosclerotic, the effect of the small molecule cyp27a1 inhibitor GW273297X^35, 36^ on atherogenesis was explored in apoE^-/-^ mice. Inhibitor treatment during week 5 to 8 of atherogenic diet feeding did not alter circulating total cholesterol or triglycerides or HDL, or the lipid profile (Supplementary Table 1, Supplementary Fig. 4d). However, in association with a decline in aorta 27HC content that paralleled a trend for a lowering with macrophage cyp27a1 silencing (Supplementary Fig. 1e, f), there was a substantial decrease in atherosclerotic lesion severity, both in en face preparations of the aorta and in the aortic root (Fig. 6a–d). Recognizing that in addition to converting cholesterol to 27HC, cyp27a1 generates cholestenoic acid^8,9^ and catalyzes the 25-hydroxylation of vitamin D3^37^, whether supplementation with 27HC reverses the effect of cyp27a1 inhibition was also evaluated. Replenishment of 27HC in inhibitor-treated mice, which did not affect circulating lipids (Supplementary Table 1, Supplementary Fig. 4e), caused an increase in lesion severity to levels observed in mice not treated with the cyp27a1 inhibitor (Fig. 6e–h). Thus, even when initiated after atherogenesis has begun, cyp27a1 inhibition is atheroprotective, and the beneficial effect is due to the resulting decline in 27HC production.

To determine if the effect of cyp27a1 inhibition on atherosclerosis is due specifically to the attenuation of macrophage enzyme activity, further experiments determined how lesions are affected by macrophage cyp27a1 silencing or inhibitor treatment alone or in combination. The individual interventions caused similar decreases in lesion abundance, and no additivity was observed (Fig. 6i–l). To determine if the disease attenuation with inhibitor treatment involves the demonstrated mechanisms related to cell-to-cell communication by macrophage-derived by 27HC and the endothelial cell ERα and septin 11 partnership, endothelial activation and vascular inflammation were evaluated. The inhibitor caused decreases in endothelial ICAM-1 and VCAM-1 expression, monocyte recruitment to lesions was blunted, and in the absence of a change in necrotic area or apoptosis, lesion macrophage accumulation was attenuated by 44% (Fig. 6m–s, Supplementary Fig. 5i–l, 6d). These observations strength the evidence that cholesterol metabolism to 27HC in macrophages is pro-atherogenic by causing endothelial cell activation to promote lesion macrophage accumulation through the stimulation of monocyte recruitment. The question arises whether there are other relevant sources of proatherogenic 27HC, but that is unlikely because pharmacologic cyp27a1 inhibition had no impact beyond the atheroprotection afforded by myeloid cell deletion of the enzyme. The findings further reveal that the mechanisms can be targeted pharmacologically to break the link between hypercholesterolemia and vascular inflammation and thereby attenuate atherosclerosis severity.

## Discussion

Although it is well-recognized that vascular inflammation, particularly mediated by NF-κB in endothelial cells, is critically involved in the pathogenesis of atherosclerosis^2,5,7^, the triggers for inflammation in the setting of hypercholesterolemia continue to be poorly understood. In the present work we show in male mice that hypercholesterolemia-driven endothelial activation, monocyte recruitment, foam cell formation and lesion development are promoted by a previously unidentified crosstalk between monocytes/macrophages and endothelial cells mediated by the cholesterol metabolite 27HC. The endothelial processes entail 27HC binding to ERα, resulting disassociation of the cytosolic adapter protein septin 11 from ERα, septin 11 recruitment to MKK7 leading to release of the inhibitory protein GADD45β, MKK7 activation which activates Jnk, and Jnk-dependent activation of NF-κB that results in the upregulation of ICAM-1 and VCAM-1 expression (Supplementary Fig. 10a). Whereas it is known that the phosphorylation of septins modifies their function^38,39^, the present work reveals a mechanism by which a member of the large family of septin proteins regulates the activity of a kinase. Parallel studies with combinatory manipulation of macrophage cyp27a1 and either endothelial ERα or endothelial septin 11 further provide an in vivo demonstration of cell-to-cell communication by 27HC, and its key role in disease pathogenesis. 27HC export from macrophages and uptake by endothelial cells was not directly measured, but the testing of the requirements for both endothelial ERα and septin 11 in entirely independent bone marrow transplant experiments evaluating the effects of monocyte/macrophage cyp27a1 provides strong evidence of 27HC-mediated cross-talk. Although it cannot be entirely ruled out, it is very unlikely that the monocyte/macrophage 27HC effect on endothelial cell phenotype and disease development via ERα and septin 11 entails an additional intermediate process.

In previous work, we observed that maneuvers that cause exaggerated elevation of 27HC in the setting of hypercholesterolemia in mice increase atherosclerosis severity^40^. This finding raised the possibility that the endogenous conversion of cholesterol to 27HC may be proatherogenic. However, other studies have suggested that 27HC generation by cyp27a1 in macrophages participates in reverse cholesterol transport, mediating cholesterol removal from macrophages and thereby being atheroprotective^10,12,13^. The latter concept has been purported to explain why a subset of individuals with cerebrotendinous xanthomatosis (CTX) due to loss-of-function mutations in cyp27a1 are at increased risk of developing atherosclerosis despite normal levels of circulating cholesterol^11,41,42^. However, besides monocytes and macrophages, cyp27a1 is abundantly expressed in the liver, small intestine and colon, and in most other tissues and cell types^42–44^. In addition, in mice in which effects of gene dosage are readily evaluated, homozygous versus heterozygous global deletion of *cyp27a1* has yielded opposing effects on atherosclerosis severity^15^. Furthermore, the 27HC generated by cyp27a1 may serve as an LXR ligand, upregulating ABCA1 leading to increased cholesterol efflux in macrophages and also stimulating anti-atherogenic processes in endothelial cells^14,45^. As such, how the enzyme influences atherosclerosis is complex. We now reveal that in macrophages, which are the primary plaque immune cell expressing cyp27a1, the enzyme promotes atherosclerosis by coupling hypercholesterolemia to endothelial activation and vascular inflammation, being responsible for the majority of macrophage accumulation in the artery wall. Single-cell RNAseq studies of human atherosclerotic lesions reveal that cyp27a1 is expressed primarily in foamy macrophages^21,22^, suggesting that the mechanisms now revealed in mice may be relevant to the disorder in humans.

The current observations suggest that 27HC is effluxed from macrophages to have paracrine action on endothelial cells. In cell culture studies it has been determined that 27HC secretion is considerably greater from macrophages compared to endothelial cells and fibroblasts, and that it requires macrophage cyp27a1. The secretion was dependent on the presence of an acceptor in the media, and it was found that both albumin and HDL are effective acceptors^12^, suggesting that they may transport 27HC in the extracellular space. Regarding 27HC acceptance by endothelial cells, if HDL-associated, it may entail the high affinity HDL acceptor SR-BI which is expressed in endothelium^46^. Since 27HC is a ligand for ERα^25,26^, its acceptance may alternatively involve that receptor. A third option is that 27HC may not require a receptor to adhere to or enter the endothelial cell because of its lipophilic nature.

The present findings revealing how hypercholesterolemia promotes vascular inflammation are directly relevant to the development of therapies to provide primary and secondary prevention of atherosclerotic cardiovascular disease. Despite successes targeting traditional atherosclerotic risk factors including hypercholesterolemia, cardiovascular disease continues to be a leading cause of death world-wide. Cardiovascular events continue to occur despite optimal lipid-lowering therapy both in broad clinical practice and in the context of controlled clinical trials^47,48^. Numerous preclinical and human biomarker studies have provided strong evidence of the contribution of inflammation to atherosclerosis pathogenesis at all stages of the disease. This has led to great recent interest in the targeting of inflammation as a means to prevent and treat atherosclerosis, with initial clinical studies appropriately evaluating the impact on recurrent events^49^. The Canakinumab Anti-inflammatory Thrombosis Outcome Study (CANTOS) was the first human trial to demonstrate that the targeting of inflammation has therapeutic validity in atherosclerosis^50^. In subjects administered canakinumab, a high-affinity anti-IL-1β monoclonal antibody, the rate of major adverse cardiovascular events was decreased. However, there was an associated increase in infections, including fatal infections, due to common bacterial and viral species. The Cardiovascular Inflammation Reduction Trial (CIRT) tested the impact of intervening against inflammation with low-dose methotrexate^51^. Because of lack of efficacy, CIRT was halted prematurely. Whereas CANTOS interestingly showed a decrease in lung cancer with the intervention, in CIRT there was an increase in cutaneous cancer with methotrexate treatment. The Colchicine Cardiovascular Outcomes Trial (COLCOT) was a third large clinical trial targeting inflammation, doing so with the natural product colchicine^52^. In COLCOT the intervention yielded a decrease in myocardial infarction, stroke and hospitalization for coronary revascularization. However, there was an increase in the occurrence of pneumonia. Therefore, the targeting of inflammatory mechanisms may provide opportunities to favorably impact atherosclerotic CVD, but approaches aimed at major inflammatory pathways run the risk of negatively affecting host response and possibly also immune surveillance for malignancy. We now demonstrate that the pharmacologic inhibition of cyp27a1 in the setting of hypercholesterolemia is antiatherogenic (Supplementary Fig. 10b), possibly revealing a means to intervene against hypercholesterolemia-related inflammation without disturbing innate or adaptive immune responses. Importantly, the effect of cyp27a1 inhibition on cancer may actually be advantageous, since we and others have shown that 27HC promotes the progression of a variety of cancers, and cyp27a1 inhibition had favorable impact on a preclinical model of breast cancer^36,53,54^. By targeting the driver of cholesterol-induced inflammation, cyp27a1 inhibition or other yet-to-be-developed interventions negating the 27HC-driven crosstalk between macrophages and endothelial NF-κB may provide an attractive, previously unpredicted means to combat the considerable risk of atherosclerotic cardiovascular disease that remains despite lipid-lowering therapies.

## Methods

Sample size was based on *n* values needed to evaluate differences between groups in prior studies. No statistical methods were used to predetermine sample size. Whenever possible, group assignments were randomized and the investigators were blinded to allocation during experiments and outcome assessment. All animal experiments were approved by the institutional animal care and use committee at UT Southwestern Medical Center.

### Animal models

Experiments were performed in wild-type, apoE^-/-^, cyp27a1^fl/fl^, ERα^fl/fl^, sept11^fl/fl^, LysM-Cre and VECad-Cre mice, or in offspring from their mating. In the generation of mice with either monocyte/macrophage gene silencing or endothelial cell gene silencing, floxed and Cre+ males were bred with floxed and Cre- females to yield study mice. Littermates were studied without versus with Cre expression. All mice were on C57BL/6 J background, and all the studies were performed in male mice to avoid possible shielding of actions of 27HC by estrogens^25,26^. The mice were housed at 23 °C with light cycles of 12 h of light beginning at 6:00am and 12 h of dark beginning at 6:00 pm, humidity was 30–70%, and H_2_O was provided ad libitum. In floxed cyp27a1 mice (cyp27a1^fl/fl^)^54^, loxP sites were inserted into intron 1 and intron 2 of the cyp27a1 gene. Floxed septin 11 mice (sept11^fl/fl^) were generated using CRISPR-Cas9 gene editing methodology to insert loxP sites in introns 2 and 3 of the septin 11 gene. In the characterization of the cyp27a1^fl/fl^ and sept11^fl/fl^ mice, the mice received intravenous injections of adeno-associated virus 8 (AAV8) vector encoding GFP driven by a liver thyroid hormone-binding globulin (TBG) promoter (AAV8-TBG-GFP) or TBG-driven Cre recombinase (AAV-TBG-Cre) at a dose of 4 × 10^11^ genome copies per mouse. AAV-TBG-GFP (Addgene plasmid # 105535) and AAV-TBG-Cre (Addgene plasmid # 107787), gifts from James M. Wilson, were packaged into 293 T cells by 3-plasmid transfection (Shuttle, Rep/Cap, pAdDeltaF6 helper), and the AAV were precipitated by PEG and purified by iodixanol density gradient at the Baylor College of Medicine Gene Vector Core. Two weeks following AAV injection, liver cyp27a1 or septin 11 expression was evaluated by immunoblotting. To generate hypercholesterolemic mice lacking cyp27a1 selectively in macrophages, cyp27a1^fl/fl^ mice were crossed with mice expressing LysM-Cre^55^, which is expressed in myeloid cells and neutrophils, with expression primarily in mature macrophages compared to monocytes^55,56^, and placed on apoE^-/-^ background. Our single cell RNAseq studies on inflammatory cells in atherosclerotic plaques in mice revealed that *cyp27a1* transcript abundance is minimal in neutrophils compare to monocytes and macrophages (Supplementary Fig. 1b, c). Thus, the primary manipulation that results from the use of LysM-Cre mice is the deletion of *cyp27a1* in macrophages (cyp27a1^∆MAC^). Effective loss of cyp27a1 in myeloid lineages was determined by immunoblotting of cyp27a1 in bone marrow-derived macrophages. To generate hypercholesterolemic mice lacking ERα selectively in endothelial cells (ERα^ΔEC^), ERα^fl/fl^ mice^57^ were crossed with VECad-Cre mice^58^ and placed on apoE^-/-^ background. A parallel approach was employed to generate hypercholesterolemic mice lacking septin 11 selectively in endothelial cells (sept11^ΔEC^). Effective selective gene excision from endothelium was evaluated by Q-RT-PCR for *septin 11* in peritoneal macrophages versus primary aortic endothelial cells. Endothelial cell deletion of *ERα* was also confirmed by Q-RT-PCR.

For studies of atherosclerosis, mice on apoE^-/-^ background were fed an atherogenic diet (D12108C, 20% fat, 1.25% cholesterol, Research Diets Inc.) for 8 weeks following weaning. To administer 27HC to mice, 27HC was purchased from Avanti Polar Lipid Inc. (Alabaster, AL) and dissolved in 30% hydroxypropyl-beta-cyclodextrin (Sigma, 332593). Vehicle or 27HC was injected subcutaneously at a dose of 20 mg/kg body weight, either every 2 days for 8 weeks in atherosclerosis studies, or daily for 3 days for in vivo intravital microscopy studies of leukocyte-endothelial cell adhesion or evaluation of impact on circulating leukocytes. For studies of cyp27a1 inhibition, the small molecule inhibitor (GX273297X)^35,36^ was synthesized from chenodeoxycholic acid by Sai Life Science, LTD and dissolved in 30% hydroxypropyl-beta-cyclodextrin, with facilitation by dispersion by mortar and pestle and sonication. Vehicle or GX273297X (100 mg/kg body weight) was administered by daily subcutaneous injection during the last 4 weeks of an 8 week period on an atherogenic diet. To determine if the effects of the inhibitor reflected the loss of 27HC production, the effect of replenishment of 27HC was determined.

To evaluate the endothelial cell requirements for the effects of macrophage-derived 27HC on atherosclerosis and other phenotypes, bone marrow transplant studies were performed employing male control cyp27a1^fl/fl^ versus cyp27a1^ΔMAC^ donors and either ERα^fl/fl^ versus ERα^ΔEC^ or sept11^fl/fl^ versus sept11^ΔEC^ recipients, all on apoE^-/-^ background. At 6 weeks of age recipients were subjected to irradiation at 400-600 Rads twice, 4 hours apart, and they then were intravenously infused with bone marrow cells pooled from same age and sex donors^59^. After 2 weeks of bone marrow reconstitution the recipients were administered the atherogenic diet, and studies were performed 8 weeks later. Bone marrow-derived macrophages were studied to evaluate the efficiency of recovery of *cyp27a1* expression versus persistent loss by immunoblotting for cyp27a1 and beta-actin.

### Cell isolation

Circulating monocytes were isolated after mice received either standard chow or atherogenic diet for 4 weeks post-weaning by previously described methods^60^. Following red blood cell removal using RBC Lysis Buffer, cells were washed X3 with flow cytometry staining (FACS) buffer (Thermo Fisher Scientific, 00-4222-26) and blocked with anti-mouse CD16/32 antibody (Biolegend, 101320, 1:100) for 10 min. Monocytes were then isolated using antibodies against CD45 (PerCP, Biolegend, 103130, Clone 30-F11, 1:100), Ly6C (FITC, Biolgend, 128006, Clone HK1.4, 1:100) and CD115 (PE/Cy7, Biolegend, 135524, Clone AFS98, 1:100). Cells were incubated with anti-CD45/Ly6C/CD155 antibody mix for 30 min on ice protected from light, and sorted using a BD FACS Aria II SORP cell sorter after three washes with FACS buffer. CD115- and Ly6C-double positive monocytes were sorted from propidium iodide (PI) negative leukocytes identified by CD45 staining. Analysis was performed with FlowJo-v10.7.2. The gating strategies for sorting CD115 + Ly6C+ cells are shown in Supplementary Fig. 2. RNA was isolated from 10,000 cells using the RNeasy Micro Kit (Qiagen, 74004), and Q-RT-PCR was performed for cyp27a1.

To obtain bone marrow-derived macrophages (BMDM), bone marrow cells were collected from the femur and tibia by flushing with serum-free RPMI1640 medium, passaged through a cell strainer, and seeded in 10 cm tissue culture plates that were then placed in a humidified incubator with 5% CO_2_ at 37 °C. BMDM differentiation was induced with recombinant macrophage colony-stimulating factor (M-CSF, 10 ng/ml) (R&D Systems, 416-ML-010/CF) in RPMI 1640 supplemented with 10% heat-inactivated FBS and 1% penicillin/streptomycin for 7 days. Cells were washed twice with PBS and differentiation media was replaced every other day.

Peritoneal macrophages were isolated from mice after feeding with either standard chow or atherogenic diet for 4 weeks post-weaning by injecting 10 ml of cold PBS buffer into the peritoneal cavity. Red blood cells were removed from the collected cells by RBC Lysing Buffer (Thermo Scientific, 00-4333-57), and macrophages were cultured in RPMI 1640 medium with 10% FBS overnight.

Endothelial cells were isolated using previously described methods^61^. Male mice at 4–5 weeks of age were anaesthetized with CO_2_ and perfused with 20 ml cold PBS via left ventricle puncture. Lung and aorta were dissected and placed in FACS buffer on ice, minced and digested by incubation in FACS buffer containing 1 mg/mL collagenase I (Thermo Fisher Scientific, 17100017), 10 mg/mL BSA, and 1 U/mL dispase (Millipore Sigma, D4693) in a revolving shaker at 37 °C for 45 min. Following passage through a 70um cell strainer, the cells were incubated with CD31 antibody (BD, 550274, 1:200) bound to Dynabeads (Thermo Fisher Scientific, 11035) for 30 min with gentle rotation every 10 min, and centrifuged at 400 xg for 5 minutes at 4 °C. Following washing of Dynabeads and related cells with FACS buffer 3 times to remove non-endothelial cells, the pellet was resuspended in Trizol for RNA isolation. All solutions used contained RNAse inhibitor (Thermo Fisher Scientific, AM2694).

### Single cell RNAseq analysis

Previously deposited **s**ingle-cell RNAseq data of CD45+ plaque leukocytes from three prior mouse studies^16–18^ were analyzed. In these studies, atherosclerotic hypercholesterolemia was induced by either global genetic deletion of LDLR or AAV-driven expression of PCSK9 in liver and western diet feeding for 14–20 weeks. To evaluate the impact on plaque immune cells of less severe hypercholesterolemia, a cohort of mice were placed on a chow diet for an additional 4 weeks in two of the studies^17,18^. Cohorts of mice receiving additional pro-resolving treatments or undergoing additional genetic modifications were removed from the analysis. This yielded 13,140 cells from across the three independent experiments passing quality filters as described in their respective publications^16–18^. All single-cell analyses were carried out using the Seurat version 4.3.0 package in R version 4.2.0. We used the sctransform function from Seurat to perform normalization and batch correction between the three datasets. Principal component analysis was carried out on the combined set of cell transcripts, and after reviewing principal component heatmaps and jackstraw plots, clustering was performed using the Seurat function FindClusters and clustering resolution was set at 0.5. Cell annotations were determined by canonical marker expression. All scRNA-seq data can be found in the Gene Expression Omnibus through accession numbers GSE141038, GSE161494 and GSE168389.

### Gene expression profiling in human arteries

To compare *cyp27a1* and septin 11 expression in human atherosclerotic versus normal arteries, two publicly-available independent human atherosclerosis cohorts with gene expression data were downloaded from the Gene Expression Omnibus (GEO, https://www.ncbi.nlm.nih.gov/geo/). The accession numbers were GSE116243 and GSE43292. For the GSE116243 cohort (Cohort I), which included atherosclerotic aortic lesions, the gene expression profiling was generated by RNA sequencing. For comparison, RNA sequencing datasets of aortas from healthy donors were downloaded from the Genotype-Tissue Expression (GTEx) database (https://gtexportal.org/home/) with accession number phs000424.v6.p1. RNA sequencing reads from FASTQ files were quality-filtered using the FASTQ Quality Filter (–q 20 –p 75) from the FASTX-toolkit (http://hannonlab.cshl.edu/fastx_toolkit/). The filtered reads were then aligned to the hg38 reference genome by HISAT2 aligner^62^. Cufflinks51 was used to assemble and estimate the relative abundance of transcripts at the gene and transcript levels^63^. For the GSE43292 cohort (Cohort II), which matched atherosclerotic carotid artery with healthy carotid artery from the same subject, gene expression profiling was generated using the Affymetrix Human Gene 1.0 ST Array. Signal intensity CEL files were downloaded from GEO and then analyzed using R/BioConductor package with RMA method and custom PERL scripts. Data are presented as box-and-whisker plots, with the central line denoting the median value, edges of the box representing upper and lower quartiles, and whiskers showing minimum and maximum values after excluding outliers outside 1.5X the interquartile range. Notably, although the analyses of the two cohorts used different technology platforms to measure gene expression, the same findings were obtained.

### Atherosclerosis, lipid and oxysterol assessments

Atherosclerosis lesions were evaluated as previously described^3^. Briefly, mice were euthanized with CO_2_, blood was collected for lipid analyses or complete blood count, and the vascular system was perfused with ice-cold phosphate-buffered saline followed by 4% paraformaldehyde solution administered by left ventricle puncture. Adventitial fat was removed using a dissecting microscope before harvesting the heart and aorta. Tissues were immersed in 10% formalin for fixation overnight, the heart was dehydrated in 30% sucrose in PBS at 4 °C overnight and embedded in optimal cutting temperature compound (OTC, Fisher Healthcare), and serial frozen sections (8 um) of the aortic root were obtained. Ten to fifteen slides with six sequential sections each were prepared from each heart, and 1-2 slides per mouse were stained with Oil Red O for assessment of aortic sinus lesions. For en face analysis, the entire aorta from the ascending aorta through the bifurcation of the iliac arteries was stained with Oil Red O for 2 h at room temperature, adjoining tissues were removed, and the aorta was opened longitudinally and pinned onto a black silicon bed. Lesions areas were quantified by morphometry of obtained images using Image J (NIH).

Serum total cholesterol and triglyceride concentrations were determined by colorimetric enzymatic assay (Infinity, Thermo Scientific). HDL levels were measured by precipitating apoB-containing lipoproteins using phosphotungstate-magnesium and measurement using the HDL Cholesterol E kit (Wako, 997-01301). Plasma lipid profiles were obtained by column fractionation and quantification of fraction cholesterol content^3^.

Oxysterols were detected and quantified in mouse serum or aorta using previously-established methods^25^.

### Histology and Immunostaining

In aortic root sections hematoxylin and eosin (H&E) staining was performed to evaluate atherosclerotic lesion size and necrotic core area. Macrophage abundance and adhesion molecule expression were assessed by blocking in buffer containing 10% donkey serum and 5% BAS for 1 h at room temperature following antigen retrieval with citric acid (pH6.0), and incubation at 4 °C overnight with the following primary antibodies: Mac2 (Cedarlane, CL8942AP, 1:600), ICAM-1 (R&D, AF796, 1:400), VCAM-1 (R&D, AF643, 1:400), and CD31 (Abcam, ab28364, 1:500). The secondary antibodies were donkey anti-rat conjugated to Alexa 488 (Thermo Fisher Scientific, A-21208, 1:600), donkey anti-goat conjugated to Alexa 647 (Thermo Fisher Scientific, A-21447, 1:600), and donkey anti-rabbit conjugated to Alexa 488 (Thermo Fisher Scientific, A-21206, 1:600). Nuclei were visualized using ProLong™ Gold Antifade mounting media containing DAPI (Thermo Fisher Scientific, P36935). All fluorescence signals were captured at the same settings using a Zeiss LSM 880 inverted confocal microscope. Confocal microscopy data analysis was by ZEN Blue Edition software. ICAM-1 and VCAM-1 fluorescence intensity overlapping with CD31 signal was quantified and normalized to CD31+ area in at least three fields per mouse using Image J. Apoptosis within the lesion was assessed by TUNEL staining performed with the In Situ Cell Death Detection Kit, TMR red (Sigma, 12156792910) following the manufacturer’s instructions.

### Monocyte recruitment

Monocyte recruitment to aortic root atherosclerotic lesions was evaluated following atherogenic diet feeding for 8 weeks using previously described methods^64^. Monocytes were labeled in vivo by retro-orbital injection with 250ul of 1 μM Fluoresbrite green fluorescent (YG) plain microspheres (Polysciences Inc., 17154) diluted 1:4 in sterile PBS. Blood was obtained 24 h later to perform flow cytometry to assess YG beads incorporation efficiency. Briefly, monocytes were identified by staining for CD45 (CD45: Phycoerythrin (PE)-Cy7, BD, 561868, Clone 30-F11, 1:100) and CD115 (CD115-PE, BD, 566839, Clone AFS98, 1:100), and characterized to be Ly6C^lo^ or Ly6C^hi^ (Ly6C-APC, BD, 560595, Clone AL-21, 1:100) by BD FACSCalibur™ Flow Cytometer. Green fluorescence revealed YG-bead abundance. The incorporation of YG bead-containing monocytes in aortic root sections was determined by visualizing green fluorescence 24 h following intravenous YG-bead injection. YG-beads per aortic root section were counted and the quantitation was corrected for labeling efficiency in circulating monocytes.

### In vivo leukocyte–endothelial adhesion

Leukocyte-endothelial adhesion was evaluated according to our previously established method^3^. Briefly, 4 week-old male mice were injected with 100 ul rhodamine-6G (0.05% w/v) via the optic vascular plexus to label leukocytes. Under anesthesia, the mesentery was exposed on a clear dish for observation and recording of images of leukocyte adhesion and rolling in the mesenteric microvasculature using a Regita digital camera (400x magnification; QImaging). The velocity of leukocyte rolling was calculated using Image-Pro V.6.2 (Media Cybernetics). In select experiments, mice were administered vehicle or 27HC (20 mg/kg body weight) daily by subcutaneous injection for 3d, and the study was performed on day 4.

### Lentivirus production

For lentivirus production, shuttle plasmid (5 μg), psPAX2 packaging plasmid (3 μg), and pMD2.G envelope plasmid (2 μg) were co-transfected into HEK-293FT cells (ThermoFisher Scientific, Cat# R0007) in DMEM using Fugene6. Six hours and 48 h post-transfection, the medium was replaced with DMEM containing 2 mM caffeine and 30% FBS. The lentiviral particles were harvested 48 h and 72 h after transfection, passed through a 0.45-μm filter (Millipore) to remove cellular debris, concentrated by ultracentrifugation at 100,000 *× g* for 90 min at 4 °C, and resuspended in Dubelcco’s phosphate-buffered saline (Gibco) overnight at 4 °C. The lentiviral preparations were then aliquotted and stored at −80 °C.

### Cell culture, gene silencing, mutanesis and reconstitution

Primary human aortic endothelial cells (HAEC; Lonza, Cat # CC-2535) were maintained in EBM2 medium supplemental with 10% (v/v) fetal bovine serum and studied at passage 3-6. Gene silencing was achieved using lentiviruses encoding shRNA targeting *ERα* (TRCN0000003300), *septin 11* (TRCN0000165270), or *Jnk1* (TRCN0000352648), and effective knockdown was confirmed by immunoblot analysis. Following initial queries made in pull-downs (see below), deletion mutant forms of septin 11 for use in intact cells were generated by site-directed mutagenesis^3^. Endogenous *septin 11* was knocked down by shRNA targeting the 3’UTR of the human septin 11 gene (TRCN0000160602), and reconstitution was then performed with wild-type or mutant forms of septin 11 using CMV promoter-driven lentiviral constructs. Polybrene (8 ug/ml, EMD Millipore) was added to the medium to enhance lentiviral transduction efficiency, and experiments were performed 48 h later.

### Quantitative RT–PCR

Transcript abundance was evaluated in mouse aorta, mouse aortic endothelial cells, peritoneal macrophages or circulating monocytes, or in HAEC by Q-RT-PCR using previously established methods^3^. Transcript levels were normalized to the abundance of mRNA for hypoxanthine-guanine phosphoribosyltransferase (HPRT). QPCR primers or company sources are provided in Supplementary Table 4.

### In vitro monocyte-endothelial cell adhesion

The adhesion of U937 monocytes to HAEC monolayers was evaluated as previously described^65^. Briefly, the monocytes were added onto the HAEC, cells were co-incubated at 37 °C with 75 rpm shaking for 20 min, non-adherent monocytes were removed by washing with fetal bovine serum (FBS, 100%), and the cells were fixed with 1% PFA for 10 min at room temperature. Images were taken randomly at 5 different positions in each well, and monocytes were counted by Image J. shRNAs were employed to knock down ERα or septin 11 expression 48 h prior to study.

### NF-kB reporter activity

Lentivirial vectors encoding an NF-kB firefly luciferase reporter (gift from Darrell Kotton, Addgene plasmid #49343) or a control renilla luciferase reporter (gift from Reuben Shaw, Addgene plasmid #74444) were produced as described above. HAEC in 96-well plates were transduced with the two reporters, employing the renilla luciferase reporter to control for infection efficiency, and 48 h later the cells were treated with vehicle, TNFα (5 ng/ml), 17β-estradiol (E2, 10^−8^M), TNFα plus E2, or 27HC (20uM) for 6 h. In select experiments, cells were also subjected to control shRNA or shRNA targeting *ERα* or *septin 11* prior to the evaluation of NF-κB reporter activity. Luciferase abundance was determined using the Dual-Glo Luciferase Assay (Promega), which sequentially measures firefly and renilla luciferase signal.

### Liquid chromatography and tandem mass spectrometry (LC-MS/MS)

To identify proteins that interact with ERα in HAEC upon E2 versus 27HC treatment, adenovirus encoding human ERα driven by a CMV promoter (ViraQuest, Inc.) was employed to overexpress the receptor. HAEC were infected with the adenovirus (MOI 200) in EBM2 medium with 2% FBS for 5 h, and EBM2 medium with 10% FBS was then added for 72 h. Subsequently the cells were treated with vehicle, E2 (10^−8^M) or 27HC (10uM) for 10 min, proteins were crosslinked using 1% formaldehyde (8 min) followed by quenching with 2 M glycine, cells were washed, proteins were extracted, and ERα was immunoprecipitated with anti-ERα antibody (HC-20, Santa Cruz Biotechnology, sc-543, 1:50). The associated proteins were evaluated by LC-MS/MS. Samples were electroporated 10 mm into the top of a pre-cast SDS-PAGE gel (Bio-Rad), stained with Coomassie blue and excised. Following alkylation and reduction with dithiothreitol and iodoacetamide (Sigma), samples were digested overnight with trypsin (Promega) and then subjected to LC–MS/MS using a QExactive mass spectrometer (Thermo Electron) coupled to an Ultimate 3000 RSLC-Nano liquid chromatography system (Dionex). Peptides were loaded onto a 180-μm i.d., 5-cm-long, self-packed column containing 1.9 μm C18 resin (Dr. Maisch, Ammerbuch, Germany) and eluted with a gradient of 0–40% buffer containing 80% (v/v) ACN, 10% (v/v) trifluoroethanol, and 0.08% formic acid for 60 min. Up to 10 high-energy, collision-induced dissociation fragment spectra were obtained for each full spectrum acquired. Raw MS data files were converted to peak list format using ProteoWizard Msconvert, and the resulting files were analyzed using the Central Proteomics Facilities Pipeline. Label-free quantification of proteins across samples was performed using the Normalized Spectral Index method (SINQ). Pertinent protein interaction dynamics were those displaying a change of 2-fold or more upon E2 or 27HC treatment.

### Co-immunoprecipitation and immunoblot analyses

Co-immunoprecipitation studies were conducted to evaluate interactions between ERα, wild-type or mutant forms of septin 11 or the Jnk kinase signaling pathway members MKK7 and GADD45β in HAEC. Cells were lysed in lysis buffer (25 mM Tris-HCl, pH 7.4, 150 mM NaCl, 1% NP-40, 1 mM EDTA, and 5% glycerol) supplemented with protease inhibitor cocktail (P8340, Sigma) on ice for 1 h with vortexing every 20 min. Supernatants collected following centrifugation at 18,000 g at 4 °C for 10 min were incubated with control IgG, anti-ERα (Santa Cruz Biotechnology, sc-543, 1:50), anti-septin 11 (Abcam, ab183529, 1:1000), or anti-MKK7 (Cell Signaling Tech, 4172, 1:1000) at 4 °C overnight. Protein A/G plus agarose beads (Pierce) were added, and supernatants and antibodies and beads were incubated for 2 h. The beads were subsequently pelleted and washed three times, and bound proteins were eluted with acid glycine. Precipitated proteins were mixed with SDS sample buffer and analyzed by SDS-PAGE and immunoblotting. Primary antibodies used were anti-ERα (Abcam, ab32063, 1:1000), anti-septin 11 (Abcam, ab183529, 1:1000), anti-MKK7 (Cell Signaling Tech, 4172, 1:1000), and anti-GADD45β (Abcam, ab205252, 1:500; Sigma, SAB2108614, 1:1000). HRP-conjugated secondary antibodies and chemiluminescent detection and densitometry were then used.

In cell culture experiments, additional immunoblot analyses were performed using the following antibodies: anti-ERα (Santa Cruz Biotechnology, sc-543, 1:1000; Abcam, ab32063, 1:1000), anti-phospho-SAPK/JNK (Thr183/Tyr185) (Cell Signaling Tech, 9251, 1:1000), anti-SAPK/JNK (Cell Signaling Tech, 9252, 1:1000), anti-JNK1(Cell Signaling Tech, 3708, 1:1000), anti-phospho-MKK7 (Ser271/Thr275; Cell Signaling Tech, 4171, 1:1000), anti-MKK7 (Cell Signaling Tech, 4172, 1:1000), anti-phospho-MLK3 (Thr277/Ser281) (Abcam, ab191530, 1:1000), anti-CYP27A1 (Abcam, ab126785, 1:1000), anti-Flag (Sigma, F1804, 1:5000), anti-HA (Sigma, H3663, 1:3000), and anti-MLK3 (Cell Signaling Tech, 2817, 1:1000). Protein loading was assessed using anti-calnexin (Enzo Life Sciences, Inc., ADI-SPA-860-F, 1:2000) or anti-GAPDH (Santa Cruz Biotechnology, sc-365062, 1:2000) or β-actin (Sigma, A1978, 1:5000) antibodies.

### Protein preparation and pull-down experiments

Expression vectors encoding HA-MKK7, flag-septin 11-WT (wild-type), and c-Myc-GADD45β (GenScript Inc.) driven by CMV were transfected into HEK-293FT cells. To test the requirement for septin 11-MKK7 interaction in the role of septin 11 in cellular responses to 27HC, flag-septin 11-∆G4 and flag-septin 11-∆GTP were also generated. All constructs were confirmed by sequencing. Seventy-two hours post-transfection, the cells were pelleted and lysed, and proteins were extracted using a buffer containing 25 mM Tris-HCl, pH 7.4, 150 mM NaCl, 1 mM EDTA, 1% NP-40, 5% glycerol, and protease inhibitors. The recombinant proteins were purified using anti-HA agarose (Thermo Fisher Scientific, 26181), anti-flag M2 affinity gel (Sigma, A2220), or anti-c-Myc agarose beads (ThermoFisher Scientific, 20168), and elution with 0.1 M glycine, pH3.5, followed by neutralization to pH 7.0. Proteins were freshly prepared prior to use. In pull-down assays the beads were pre-blocked with 1% BSA to reduce nonspecific binding, and the bait protein was added and incubated with beads for 1 h at 37 °C. After extensive washing the prey protein(s) was added and incubation resumed at 37 °C for 1 h. Following washing X3, protein complexes were eluted and samples were subjected to SDS-PAGE and immunoblot analysis.

### Statistics and reproducibility

Results are expressed as mean ± SEM. Comparisons between two groups were done by two-sided unpaired Student’s t-test or by two-tailed unpaired Mann-Whitney U-test for data with normal and non-normal distribution, respectively. Three or more groups were compared by one-way analysis of variance (ANOVA) with Tukey’s post-hoc test or by Krusakal-Wallis with Dunn’s post-hoc testing for data with normal and non-normal distribution, respectively. Statistical analysis was performed using Graph-Pad Prism8 or a higher version. *P* < 0.05 was considered statistically significant. When representative findings are presented, similar results were obtained at least three times. Observations in cell culture were confirmed in three independent experiments.

### Reporting summary

Further information on research design is available in the Nature Portfolio Reporting Summary linked to this article.

## Supplementary information

Supplementary Information

Peer Review File

Description of Additional Supplementary Files

Supplementary Movie 1

Supplementary Movie 2

Supplementary Movie 3

Supplementary Movie 4

Supplementary Movie 5

Supplementary Movie 6

Reporting Summary

## Data Availability

The raw LC/MS-MS data in this study has been uploaded to the MassIVE data repository with accession number MSV000091880 at https://massive.ucsd.edu/ProteoSAFe/dataset.jsp?task=aba429f8538a465a85a530f075ec7287. The publicly available scRNA-seq data re-analyzed in this study has been deposited in the Gene Expression Omnibus database under the accession codes GSE141038^16^, GSE161494^17^ and GSE168389^18^ at https://www.ncbi.nlm.nih.gov/geo/query/acc.cgi?acc=GSE141038, https://www.ncbi.nlm.nih.gov/geo/query/acc.cgi?acc=GSE161494 and https://www.ncbi.nlm.nih.gov/geo/query/acc.cgi?acc=GSE168389. Source data are provided with this paper.

## Source data

Source Data
