# Supplementary material for: Macrophage-to-endothelial cell crosstalk by the cholesterol metabolite 27HC promotes atherosclerosis in male mice
Source: Nat Commun. 2023 Jul 25;14:4101. doi: 10.1038/s41467-023-39586-z (PMC10368733; doi:10.1038/s41467-023-39586-z)

Supplementary Information Table 1: Serum Lipids

Genotype / Treatment	N	Total Cholesterol (mg/dL)	Triglyceride (mg/dL)	HDL – cholesterol (mg/dL)
cyp27a1$^{fl/fl}$	8	1222.68+/-49.50	94.66+/-6.99	49.38+/-5.42
cyp27a1$^{\Delta Mac}$	10	1248.86+/-47.63	111.14+/-8.88	46.00+/-6.27
ERa$^{fl/fl}$ Veh.	13	1080.67+/-33.79	80.52+/-11.79	23.19+/-3.14
ERa$^{fl/fl}$ 27HC	14	1050.76+/-17.26	65+/-3.81	17.05+/-0.31
ERa$^{\Delta EC}$ Veh.	10	1079.2+/-27.25	80.12+/-8.48	19.44+/-0.92
ERa$^{\Delta EC}$ 27HC	12	1024.17+/-35.29	59.41+/-3.95	18.23+/-0.78
Donor cyp27a1$^{\Delta MAC}$ Recip.ERa$^{\Delta EC}$	12	1049.08+/-37.07	101.68+/-14.03	44.56+/-4.96
Donor cyp27a1$^{\Delta MAC}$ Recip. ERa$^{fl/fl}$	12	1022.15+/-38.22	113.41+/-14.14	39.96+/-5.24
Donor cyp27a1$^{fl/fl}$ Recip. ERa$^{\Delta EC}$	11	1040.80+/-42.16	96.77+/-14.50	35.40+/-4.59
Donor cyp27a1$^{fl/fl}$ Recip. ERa$^{fl/fl}$	11	1046.2+/-33.09	99.92+/-14.46	39.26+/-4.35
sept11$^{fl/fl}$	10	896.52+/-41.62	71.52+/-9.43	40.45+/-2.82
sept11$^{\Delta EC}$	12	957.88+/-25.29	88.91+/-7.30	45.65+/-3.25
Donor cyp27a1$^{\Delta MAC}$ Recip. sept11$^{\Delta EC}$	12	993.08+/-40.99	112.91+/-8.89	31.97+/-3.39
Donor cyp27a1$^{\Delta MAC}$ Recip. sept11$^{fl/fl}$	10	1033.80+/-45.86	103.76+/-10.37	31.19+/-2.24
Donor cyp27a1$^{fl/fl}$ Recip. sept11$^{\Delta EC}$	11	909.30+/-44.35	101.67+/-13.75	35.42+/-1.92
Donor cyp27a1$^{fl/fl}$ Recip. sept11$^{fl/fl}$	10	964.64+/-35.56	98.80+/-11.43	35.45+/-3.17
Veh.	8	1070.57+/-69.36	98.85+/-7.92	41.82+/-4.77
GW	10	990.43+/-41.93	100.97+/-8.61	37.79+/-3.68
3 Groups: Veh.	9	981.48+34.46	53.76+/-5.11	31.58+/-2.29
3 Groups: GW	12	992.83+/-21.33	55.44+/-4.42	33.18+/-1.30
3 Groups: GW+27HC	13	926.77+/-20.76	57.94+/-4.25	31.73+/-0.86
cyp27a1$^{fl/fl}$ Veh.	9	993.53+/-27.66	112.88+/-5.65	36.50+/-3.08
cyp27a1$^{fl/fl}$ GW	11	937.40+/-29.29	119.27+/-5.19	33.48+/-3.24
cyp27a1$^{\Delta MAC}$ Veh.	10	948.35+/-23.16	117.27+/-6.30	36.45+/-3.70
cyp27a1$^{\Delta MAC}$ GW	11	1013.92+/-34.53	117.78+/-6.66	35.28+/-3.10

All mice were apoE null background and fed an atherogenic diet.

Supplementary Information Table 2: ERα Interactomes: Same Direction of Change

Increased ERα Association			Decreased ERα Association		
With E2 Only	With 27HC Only	In Common with E2 and 27HC	With E2 Only	With 27HC Only	In Common with E2 and 27HC
AACS	APRT	DDB1	AHSG	AACS	A2M
ACAT2	ARF5	FKBP10	CACYBP	AAK1	AAMP
ACTBL2	CDSN	HSP90AA1	DNAJA2	AARS1	ABCE1
AHNAK	DCD	HSPA8	IRGQ	ABCF1	ABCF3
AKT1	DSG1	INPP1	KIF1B	ABHD14B	ACAT1
ALDH1A3	DSP	ITGB1	MT2A	ACAA2	ACBD3
ASAP1	FLG2	KIF1B	TARDBP	ACADVL	ACO1
ATP5F1B	H2AC4	KRT75	TOMM34	ACLY	BACH
CAD	H3-3A	KRT9	TUBB4B	ACTBL2	ACTR1A
CALML3	H4C1	SEMG1	XPO5	ACTN1	ACTR2
CALML5	IGLL5	TTLL12		ACTN4	ADSL
CASP14	KRT10	TUBB		ACTR3	AGFG1
CMPK1	KRT17	TUBB2A		ADK	AHCYL2
CRABP2	KRT80	B3KVR1		ADRM1	AKAP12
CS	MCMBP	F5H4X1		ADSS2	AKR1B1
CSTB	PKP1	J3QST3		AHCY	AKR7A2
DCPS	RAB3GAP1	IGHG1		AHNAK	ANXA3
DNM2	RPS5	IGHG4		AHSA1	AP1B1
EHD2	RPS9	HSP71		AK1	AP2A2
EIF3B	SLC25A5			ALDH1A3	AP2M1
EIF3G	TUBA1A			ALDH7A1	APOL2
EPPK1	TUBB4A			ALDOA	ARCN1
ERO1A	B4DR52			ALDOC	ARHGAP17
ETFB				ANXA1	ARHGAP5
F5				ANXA2	ARHGDIA
GLUL				AP2A1	ARHGDIB
GSDMA				API5	ARPC1A
HNRNPA2B1				APOB	ARPC1B
HSPA5				ARHGAP1	ARPC2
IDH1				ARHGAP18	ASAP2
INF2				ARHGEF1	ASNS
JUP				ATP5F1B	ATG3
KPRP				ATP6V1B2	ATG7
KRT16				ATP6V1H	ATP1A1

KRT18				ATXN10	ATP6V1C1
KRT23				BCAR1	ATP6V1E1
KRT5				BCL2L1	ATXN2L
KRT6A				C3	BAG2
KRT6B				C4B_2	BAG3
KRT77				CAD	BASP1
KRT78				CAND1	BCL10
KRT79				CAP1	BIN1
LGALS7				CAPN1	BLVRA
MDH1				CAPN2	BORCS6
MDH2				CAPNS1	BTAF1
MRI1				CAPZA1	CAPG
MTA2				CAPZB	CAPRIN1
MYL6				CBR1	CARM1
NCCRP1				CCAR1	CASP4
NUBP2				CCT2	CBR3
PDE12				CCT3	CCAR2
PEF1				CCT5	CCDC43
PGM2				CCT6A	CCDC50
PLEC				CCT7	CCDC6
POF1B				CCT8	CCND3
PPL				CD9	CCNDBP1
PRDX2				CDC123	CDK1
PREP				CDK16	CDV3
PRRC1				CDK2	CEP170
PSMA2				CFL1	CHMP1A
PSMB2				CHMP4B	CHMP5
PSMB6				CKAP5	CKB
PSMC3				CLIC1	CLASP2
PTGES3L-AARSD1				CLIC4	CLIC3
QRICH1				CLTC	CLIP1
RAB10				CNDP2	CLUH
RAB5B				CNN2	COL1A1
RACK1				CNN3	COL1A2
RANBP3				COPB1	COPA
RBBP7				COPS2	COPG1
RPA1				COPS7A	COPS3
RPL24				CPNE3	COPS4
RPL6				CS	COPS6
RPRD1B				CSE1L	COPS7B
RPS2				CSTF2	CORO1C
RPS3				CTPS1	CORO7

RPS8				CUL3	CPNE1
S100A8				CYFIP1	CRIP2
S100A9				CYRIB	CSK
SEPTIN11				DDX19A	CTBP2
SERPINB3				DDX3X	CTNND1
SERPINB4				DDX6	CTTN
SERPINB6				DHX15	CUL2
SFN				DHX9	CUL4B
SLC25A3				DNM1L	CYRIA
SNRNP200				DPYSL2	DAB2
SNTB2				DPYSL3	DREB
SNX3				DSC1	DBNL
SPRR1B				DTYMK	DDX1
SPTBN1				EEF2	DFFA
SRRT				EHD1	DGKA
SUCLG2				EHD2	DHX29
SUPT6H				EHD4	DIS3L2
TAF15				EIF2S1	DLGAP4
TAGLN				EIF2S3	DNAJB1
TAGLN2				EIF3CL	DNAJB4
TGM1				EIF3G	DNAJC3
TJP1				EIF3H	DNAJC7
TNPO2				EIF4A1	DNM2
TPI1				EIF4A3	DRG1
TRAPPC5				EIF4E	DYM
TRIM29				EIF5	DYNC1H1
TRIP12				EIF5B	DYNC1LI1
VDAC1				EIF6	ECD
VPS29				ENO1	ECHDC1
WASF2				EPN1	EEF1B2
A2A376				EPRS1	EFHD2
A6NEC2				ESD	U5S1
B4DDF9				ETHE1	EIF2AK2
B4DJA5				F2	EIF2B2
B4DMK0				FABP5	EIF2S2
B7Z2X9				FAM114A1	EIF3E
F2Z2U8				FASN	EIF3I
F8W6I7				FDPS	EIF3J
G8JLA8				FDXR	EIF3L
H7BYP0				FEN1	EIF4G2
J3KPN6				FERMT2	EIF4H
K7ENP3				FERMT3	EIF5A

M0QZK8				FH	ELP1
IGHG2				FHOD1	ETF1
IGHA1				FKBP4	EZR
LAC2				FLNA	FAF1
Q3SYB4				FLNC	FAM114A2
YJ005				FSCN1	FAM50A
DECOY1_75073				FUBP1	FARSB
				FXR2	FBLIM1
				G6PD	FHL1
				GARS1	FKBP15
				GET4	FKBP5
				GGA3	FLII
				GLRX3	CG055
				GMPPA	FOXK1
				GPN1	FUS
				GPS1	GALE
				GRHPR	GAPVD1
				GSK3A	GART
				GSK3B	GBF1
				GYG1	GBP1
				HAT1	GC
				HDLBP	GCLM
				HNRNPA3	GCN1
				HNRNPD	GET3
				HNRNPK	GFPT1
				HNRNPM	GIPC1
				HNRNPM	GMPPB
				HNRNPU	GNAI2
				HOOK3	GNAI3
				HSP90B1	GOLPH3
				HSPA1L	GSDME
				HSPA5	ERF3A
				HSPA9	GSPT2
				HSPB1	GSTM1
				IARS1	GSTM3
				IDH1	GSTO1
				IMPA2	GTF2F1
				IMPDH2	GYS1
				INF2	HBS1L
				IPO4	HCLS1
				IPO7	HECTD1
				IPO9	HGS

				IQGAP1	HIP1R
				ITGA5	ROAA
				ITIH4	HNRNPDL
				KHSRP	HSD17B10
				KIF5B	HSPA14
				KIFBP	HSPBP1
				KPNA1	HSPG2
				KPNA2	HTRA1
				KPNA3	HUWE1
				KPNA4	IDE
				KPNB1	IGBP1
				KRT18	ILK
				KRT6A	ILKAP
				LDHA	ITIH2
				LDHB	KCTD12
				LPP	KIF13B
				LUC7L3	KIF1C
				MAGED2	KLC2
				MAP2K1	KPNA6
				MAP2K2	KRT7
				MAP4	LARS1
				MAP7D1	LASP1
				MAPK1	LCP1
				MAPRE1	LGALS1
				MCAM	LIMA1
				MDH1	LPXN
				MIF	LRRC40
				MMS19	LRRC47
				MSN	LRRC57
				MTHFD1	LRRF1
				MVP	LTA4H
				MYH9	MAD2L1
				MYO1C	MAGED1
				MYO9B	MAP1B
				NAGK	MTAP2
				NAMPT	MAP2K3
				NAPRT	MAP2K4
				NCKAP1	MAP4
				NCL	MARCKS
				NIBAN2	MAT2A
				NNMT	MCM3
				NONO	MCM4

				NPLOC4	MCM6
				NRBP1	MEMO1
				NUBP2	MGLL
				NUP93	MICAL1
				OTUB1	MX1
				P4HB	MYO1E
				PA2G4	NAPA
				PAFAH1B2	NEK7
				PAFAH1B3	NFKB1
				PAICS	NFKB2
				PAK2	NSFL1C
				PALM2AKAP2	NUBP1
				PAPOLA	NUDC
				PAPSS1	NUDCD1
				PCBP1	NUDCD3
				PDCD6IP	NXN
				PDCL3	OLA1
				PDE12	OPTN
				PDIA3	OSBP
				PFKL	OTUD6B
				PFKM	OXCT1
				PFKP	OXSR1
				PFN1	PABPC4
				PGK1	PACS1
				PGLS	PACSIN2
				PGP	PAIP1
				PHGDH	PALMD
				PICALM	PBDC1
				PIK3C2A	PCMT1
				PKM	PCNA
				PLAA	PCNP
				PLEC	PCYT1A
				PLG	PDLIM1
				PLS3	PDLIM4
				POSTN	PDLIM5
				PPP2CB	PDS5A
				PPP2R1A	PKN1
				PPP2R2A	PKN2
				PPP3CA	PLCG1
				PPP5C	POLR1C
				PPP6R1	POLR2B
				PRDX6	PPM1F

				PREP	PPM1G
				PRKAR2A	PPP1CA
				PRKAR2B	PPP1R12A
				PRPSAP1	PPP1R13L
				PRRC1	PPP1R18
				PSMA1	PPP1R7
				PSMA2	PPP2R5D
				PSMA3	PPP4R1
				PSMA4	PPP6R3
				PSMA7	PRKACB
				PSMB2	PRKAR1A
				PSMB4	PRKCA
				PSMB5	PRMT1
				PSMC2	PRPS2
				PSMC4	PSMA5
				PSMC5	PSMC1
				PSMD1	PSMC3
				PSMD12	PSMC6
				PSMD13	PSMD14
				PSMD2	PSMD6
				PSMD3	PTGR1
				PSMD4	PTPN11
				PSMD5	PXN
				PSMD8	PYGB
				PSME1	RAD23B
				PSME2	RAP1GDS1
				PSMF1	RBM39
				PSMG1	RELA
				PTBP1	RIPK2
				PTGES3L-AARSD1	RIPOR1
				PTPN12	RNPEP
				PUF60	RPL4
				PYGL	RPL7
				QRICH1	RPS6KA3
				RAB3GAP2	RRAGC
				RAB5C	RRM2
				RACK1	RSU1
				RAN	RTCB
				RANBP3	SAMD9
				RANGAP1	SCRN1
				RARS1	SDSL
				RASIP1	SEC23A

				RBBP4	SEC23B
				RBBP7	SETD3
				RBM12	SF1
				RECQL	SH3GLB1
				RNF103-CHMP3	SHC1
				RNH1	SHTN1
				RPA1	SLFN5
				RPAP3	SLK
				RPL10A	SMS
				RPL13	SNRPA1
				RPS2	SNX6
				RPS3	SP100
				RPS4X	SPAG9
				RPS6	SPAST
				RPS8	SPATA5
				RRM1	SPR
				RRM2B	SQSTM1
				RTCA	SRM
				RUVBL2	SRSF11
				S100A8	ST13
				SAE1	STAT1
				SAR1B	STAT6
				SARS1	STK10
				SBDS	STK17A
				SEC24A	STK24
				SEC24D	STK26
				SEPTIN11	STK39
				SEPTIN2	STUB1
				SEPTIN9	STXBP1
				SERPINB8	SULT1A1
				SERPINE1	SWAP70
				SERPINH1	SYAP1
				SF3A1	TAOK3
				SF3B1	TARS1
				SF3B3	TBC1D9B
				SFPQ	TBCB
				SGTA	TBCD
				SH3GL1	THBS1
				SH3PXD2B	TIAL1
				SHMT2	TLN2

				SIRT2	TMOD3
				SMTN	TPM2
				SNAP29	TPM4
				SND1	TRIM28
				SNRNP200	TRIP13
				SNX17	TSC22D2
				SNX3	TTC1
				SPART	TTN
				SRP54	TUBA1C
				SRP68	TXNDC9
				SRSF6	UAP1
				STAU1	UBE2M
				STIP1	UBE3C
				STMN1	UBE4B
				SUGT1	UBFD1
				SULT1B1	UBQLN1
				SUPT6H	UBQLN2
				SYNCRIP	UBQLN4
				TBCC	UBR4
				TCERG1	UBXN1
				TCP1	UBXN7
				TFG	UCHL1
				TGM2	UCHL5
				THBS4	UGP2
				TIGAR	UNC45A
				TJP2	UPF1
				TKT	USP14
				TLN1	USP25
				TMPO	USP9X
				TNKS1BP1	VASP
				TNPO1	VCPIP1
				TPI1	VPS4A
				TRAPPC5	VTA1
				TRIM25	WARS1
				TRIP12	WDR1
				TUFM	WNK1
				TWF1	WNK4
				TXNL1	WWTR1

				UBA2	XPO1
				UBA5	YARS1
				UBAP2L	YBX1
				UBE2E3	YRDC
				UBE2O	YTHDF3
				UBE2S	1433B
				UBE2Z	YWHAH
				UCHL3	ZC3HAV1
				UCK2	ZPR1
				UGDH	A6NCA8
				UMPS	A6NFN2
				UROD	A6NN80
				USO1	A8MTP3
				USP5	B1AKJ5
				VARS1	B2RPK0
				VAT1	B3KQZ9
				VCL	B3KRS5
				VCP	B3KSH1
				VPS35	B3KXW5
				VPS4B	B4DDM6
				WASF2	B4DG50
				WASHC2A	B4DGB4
				XRCC5	B4DLW8
				XRCC6	B4DS13
				YKT6	B4DTL2
				YTHDF2	B4DUS9
				YWHAE	B4DUX5
				YWHAG	B4DVY1
				YWHAQ	B4DWJ2
				ZNF185	B4DX29
				ZYX	B4DXI8
				A2A376	B4DXJ1
				A6NE09	B4DXX7
				A6NG51	B4DZI8
				A6NKH4	B4E0X2
				A8MUB1	B4E2Q4
				B0UX83	B4E363
				B3KVK7	B5MCQ5

				B3KWE1	B7Z1R5
				B4DF38	B7Z341
				B4DFA2	B7Z3P1
				B4DFC9	B7Z419
				B4DFL2	B7Z8H8
				B4DH53	B7ZKK9
				B4DKT0	B7ZKM0
				B4DL37	B7ZKM8
				B4DNJ6	B9ZVX0
				B4DQJ8	C9JZR4
				B4DT77	E5RJ68
				B4DVE7	E7EMH5
				B4DXJ9	E7EPD0
				B4DZP4	E7EPK1
				B4E1G6	E7EQD5
				B4E2V5	E7ESA8
				B7Z2X9	E7EU23
				B7ZA04	E7EUT8
				E7ENR4	E7EW20
				E7EP00	E9PC74
				E7ER89	E9PL22
				E7ES43	E9PM46
				E7EUY0	E9PR44
				E7EVH7	F5GWS3
				F5GWY2	F5GX05
				F5GXC8	F5H1Z9
				F5H335	F5H2A7
				F5H564	F5H2S7
				F5H897	F5H5M9
				F8VWC5	F8W914
				F8W6I7	G5E9I6
				F8W720	G5E9Q2
				F8W7M9	H0Y4G9
				F8W845	H0YL12
				G8JLA8	H0YLA4
				H3BLZ8	J3KNW4
				H7BYP0	Q5VY93
				H7BZT4	Q75MJ1

				J3KNQ4	PKN1
				J3QT22	GDS1
				K7EKV8	DBNL
				K7ENL3	TPM4
				K7ENP3	FLII
				IGHA1	AP1B1
				F203B	LRRF1
				Q3SYB4	DECOY1_74907
				H90B4	
				Q6IC75	
				YJ005	
				DECOY1_2717	
				DECOY1_75073	

Supplementary Information Table 3: ERα Interactomes: Opposite Direction of Change

Decreased ERα Association with E2	Increased ERα Association with 27HC	Increased ERα Association with E2	Decreased ERα Association with 27HC	Overlap of Increased Association with E2 and Decreased Association with 27HC
AHSG	APRT	ACAT2	AAK1	AACS
CACYBP	ARF5	AKT1	AARS1	ACTBL2
DNAJA2	CDSN	ASAP1	ABCF1	AHNAK
IRGQ	DCD	CALML3	ABHD14B	ALDH1A3
KIF1B	DSG1	CALML5	ACAA2	ATP5F1B
MT2A	DSP	CASP14	ACADVL	CAD
TARDBP	FLG2	CMPK1	ACLY	CS
TOMM34	H2AC4	CRABP2	ACTN1	EHD2
TUBB4B	H3-3A	CSTB	ACTN4	EIF3G
XPO5	H4C1	DCPS	ACTR3	HSPA5
	IGLL5	DNM2	ADK	IDH1
	KRT10	EIF3B	ADRM1	INF2
	KRT17	EPPK1	ADSS2	KRT18
	KRT80	ERO1A	AHCY	KRT6A
	MCMBP	ETFB	AHSA1	MDH1
	PKP1	F5	AK1	NUBP2
	RAB3GAP1	GLUL	ALDH7A1	PDE12
	RPS5	GSDMA	ALDOA	PLEC
	RPS9	HNRNPA2B1	ALDOC	PREP
	SLC25A5	JUP	ANXA1	PRRC1
	TUBA1A	KPRP	ANXA2	PSMA2
	TUBB4A	KRT16	AP2A1	PSMB2
	B4DR52	KRT23	API5	PTGES3L-AARSD1
		KRT5	APOB	QRICH1
		KRT6B	ARHGAP1	RACK1
		KRT77	ARHGAP18	RANBP3
		KRT78	ARHGEF1	RBBP7
		KRT79	ATP6V1B2	RPA1
		LGALS7	ATP6V1H	RPS2
		MDH2	ATXN10	RPS3
		MRI1	BCAR1	RPS8
		MTA2	BCL2L1	S100A8
		MYL6	C3	SEPTIN11
		NCCRP1	C4B_2	SNRNP200
		PEF1	CAND1	SNX3
		PGM2	CAP1	SUPT6H
		POF1B	CAPN1	TPI1
		PPL	CAPN2	TRAPPC5
		PRDX2	CAPNS1	TRIP12
		PSMB6	CAPZA1	WASF2
		PSMC3	CAPZB	A2A376
		RAB10	CBR1	B7Z2X9
		RAB5B	CCAR1	F8W6I7
		RPL24	CCT2	G8JLA8
		RPL6	CCT3	H7BYP0
		RPRD1B	CCT5	K7ENP3
		S100A9	CCT6A	IGHA1

		SERPINB3	CCT7	Q3SYB4
		SERPINB4	CCT8	YJ005
		SERPINB6	CD9	DECOY1_75073
		SFN	CDC123	
		SLC25A3	CDK16	
		SNTB2	CDK2	
		SPRR1B	CFL1	
		SPTBN1	CHMP4B	
		SRRT	CKAP5	
		SUCLG2	CLIC1	
		TAF15	CLIC4	
		TAGLN	CLTC	
		TAGLN2	CNDP2	
		TGM1	CNN2	
		TJP1	CNN3	
		TNPO2	COPB1	
		TRIM29	COPS2	
		VDAC1	COPS7A	
		VPS29	CPNE3	
		A6NEC2	CSE1L	
		B4DDF9	CSTF2	
		B4DJA5	CTPS1	
		B4DMK0	CUL3	
		F2Z2U8	CYFIP1	
		J3KPN6	CYRIB	
		M0QZK8	DDX19A	
		IGHG2	DDX3X	
		LAC2	DDX6	
			DHX15	
			DHX9	
			DNM1L	
			DPYSL2	
			DPYSL3	
			DSC1	
			DTYMK	
			EEF2	
			EHD1	
			EHD4	
			EIF2S1	
			EIF2S3	
			EIF3CL	
			EIF3H	
			EIF4A1	
			EIF4A3	
			EIF4E	
			EIF5	
			EIF5B	
			EIF6	
			ENO1	
			EPN1	
			EPRS1	
			ESD	
			ETHE1	
			F2	
			FABP5	
			FAM114A1	

			FASN	
			FDPS	
			FDXR	
			FEN1	
			FERMT2	
			FERMT3	
			FH	
			FHOD1	
			FKBP4	
			FLNA	
			FLNC	
			FSCN1	
			FUBP1	
			FXR2	
			G6PD	
			GARS1	
			GET4	
			GGA3	
			GLRX3	
			GMPPA	
			GPN1	
			GPS1	
			GRHPR	
			GSK3A	
			GSK3B	
			GYG1	
			HAT1	
			HDLBP	
			HNRNPA3	
			HNRNPD	
			HNRNPK	
			HNRNPM	
			HNRNPM	
			HNRNPU	
			HOOK3	
			HSP90B1	
			HSPA1L	
			HSPA9	
			HSPB1	
			IARS1	
			IMPA2	
			IMPDH2	
			IPO4	
			IPO7	
			IPO9	
			IQGAP1	
			ITGA5	
			ITIH4	
			KHSRP	
			KIF5B	
			KIFBP	
			KPNA1	
			KPNA2	
			KPNA3	
			KPNA4	
			KPNB1	

			LDHA	
			LDHB	
			LPP	
			LUC7L3	
			MAGED2	
			MAP2K1	
			MAP2K2	
			MAP4	
			MAP7D1	
			MAPK1	
			MAPRE1	
			MCAM	
			MIF	
			MMS19	
			MSN	
			MTHFD1	
			MVP	
			MYH9	
			MYO1C	
			MYO9B	
			NAGK	
			NAMPT	
			NAPRT	
			NCKAP1	
			NCL	
			NIBAN2	
			NNMT	
			NONO	
			NPLOC4	
			NRBP1	
			NUP93	
			OTUB1	
			P4HB	
			PA2G4	
			PAFAH1B2	
			PAFAH1B3	
			PAICS	
			PAK2	
			PALM2AKAP2	
			PAPOLA	
			PAPSS1	
			PCBP1	
			PDCD6IP	
			PDCL3	
			PDIA3	
			PFKL	
			PFKM	
			PFKP	
			PFN1	
			PGK1	
			PGLS	
			PGP	
			PHGDH	
			PICALM	
			PIK3C2A	
			PKM	

			PLAA	
			PLG	
			PLS3	
			POSTN	
			PPP2CB	
			PPP2R1A	
			PPP2R2A	
			PPP3CA	
			PPP5C	
			PPP6R1	
			PRDX6	
			PRKAR2A	
			PRKAR2B	
			PRPSAP1	
			PSMA1	
			PSMA3	
			PSMA4	
			PSMA7	
			PSMB4	
			PSMB5	
			PSMC2	
			PSMC4	
			PSMC5	
			PSMD1	
			PSMD12	
			PSMD13	
			PSMD2	
			PSMD3	
			PSMD4	
			PSMD5	
			PSMD8	
			PSME1	
			PSME2	
			PSMF1	
			PSMG1	
			PTBP1	
			PTPN12	
			PUF60	
			PYGL	
			RAB3GAP2	
			RAB5C	
			RAN	
			RANGAP1	
			RARS1	
			RASIP1	
			RBBP4	
			RBM12	
			RECQL	
			RNF103-CHMP3	
			RNH1	
			RPAP3	
			RPL10A	
			RPL13	
			RPS4X	
			RPS6	
			RRM1	

			RRM2B	
			RTCA	
			RUVBL2	
			SAE1	
			SAR1B	
			SARS1	
			SBDS	
			SEC24A	
			SEC24D	
			SEPTIN2	
			SEPTIN9	
			SERPINB8	
			SERPINE1	
			SERPINH1	
			SF3A1	
			SF3B1	
			SF3B3	
			SFPQ	
			SGTA	
			SH3GL1	
			SH3PXD2B	
			SHMT2	
			SIRT2	
			SMTN	
			SNAP29	
			SND1	
			SNX17	
			SPART	
			SRP54	
			SRP68	
			SRSF6	
			STAU1	
			STIP1	
			STMN1	
			SUGT1	
			SULT1B1	
			SYNCRIP	
			TBCC	
			TCERG1	
			TCP1	
			TFG	
			TGM2	
			THBS4	
			TIGAR	
			TJP2	
			TKT	
			TLN1	
			TMPO	
			TNKS1BP1	
			TNPO1	
			TRIM25	
			TUFM	
			TWF1	
			TXNL1	
			UBA2	
			UBA5	

			UBAP2L	
			UBE2E3	
			UBE2O	
			UBE2S	
			UBE2Z	
			UCHL3	
			UCK2	
			UGDH	
			UMPS	
			UROD	
			USO1	
			USP5	
			VARS1	
			VAT1	
			VCL	
			VCP	
			VPS35	
			VPS4B	
			WASHC2A	
			XRCC5	
			XRCC6	
			YKT6	
			YTHDF2	
			YWHAE	
			YWHAG	
			YWHAQ	
			ZNF185	
			ZYX	
			A6NE09	
			A6NG51	
			A6NKH4	
			A8MUB1	
			B0UX83	
			B3KVK7	
			B3KWE1	
			B4DF38	
			B4DFA2	
			B4DFC9	
			B4DFL2	
			B4DH53	
			B4DKT0	
			B4DL37	
			B4DNJ6	
			B4DQJ8	
			B4DT77	
			B4DVE7	
			B4DXJ9	
			B4DZP4	
			B4E1G6	
			B4E2V5	
			B7ZA04	
			E7ENR4	
			E7EP00	
			E7ER89	
			E7ES43	
			E7EUY0	

			E7EVH7	
			F5GWY2	
			F5GXC8	
			F5H335	
			F5H564	
			F5H897	
			F8VWC5	
			F8W720	
			F8W7M9	
			F8W845	
			H3BLZ8	
			H7BZT4	
			J3KNQ4	
			J3QT22	
			K7EKV8	
			K7ENL3	
			F203B	
			H90B4	
			Q6IC75	
			DECOY1_2717	

Supplementary Table 4: Quantitative RT-PCR SYBR Green and TaqMan Resources

	Gene Name	Primer Sequence (5' -> 3')	Reference
SYBR Green	mouse CD31 Forward	CTGCCAGTCCGAAAATGGAAC	PrimerBank ID: 6679273a1
	mouse CD31 Reverse	CTTCATCCACTGGGGCTATC	
	mouse Cyp27a1 Forward	CCAGGCACAGGAGAGTACG	PrimerBank ID: 30578401a1
	mouse Cyp27a1 Reverse	GGGCAAGTGCAGCACATAG	
	mouse HPRT Forward	AGTCCCAGCGTCGTGATTAG	PrimerBank ID: 96975137c1
	mouse HPRT Reverse	TTTCCAAATCCTCGGCATAATGA	
	mouse Septin 11 Forward	GACCCAGCTACTCACAACGAG	PrimerBank ID: 57634517c2
	mouse Septin 11 Reverse	TGGGCGTCAATGTATTCCACT	
	mouse ERα Forward	CCTCCCGCCTTCTACAGGT	PrimerBank ID: 6679695a1
	mouse ERα Reverse	CACACGGCACAGTAGCGAG	
		Cat# & Assay ID	
TaqMan	mouse ABCA1	4331182, Mm00442646_m1	
	mouse ABCG1	4331182, Mm00437390_m1	
	mouse NR1H3	4331182, Mm00443451_m1	
	mouse NR1H2	4331182, Mm00437265_g1	
	mouse SREBP1c	4331182, Mm00550338_m1	
	mouse APOE	4331182, Mm01307193_g1	
	mouse FASN	4331182, Mm00662319_m1	
	mouse HPRT	4331182, Mm03024075_m1	

SUPPLEMENTARY FIGURES

Supplementary Fig. 1

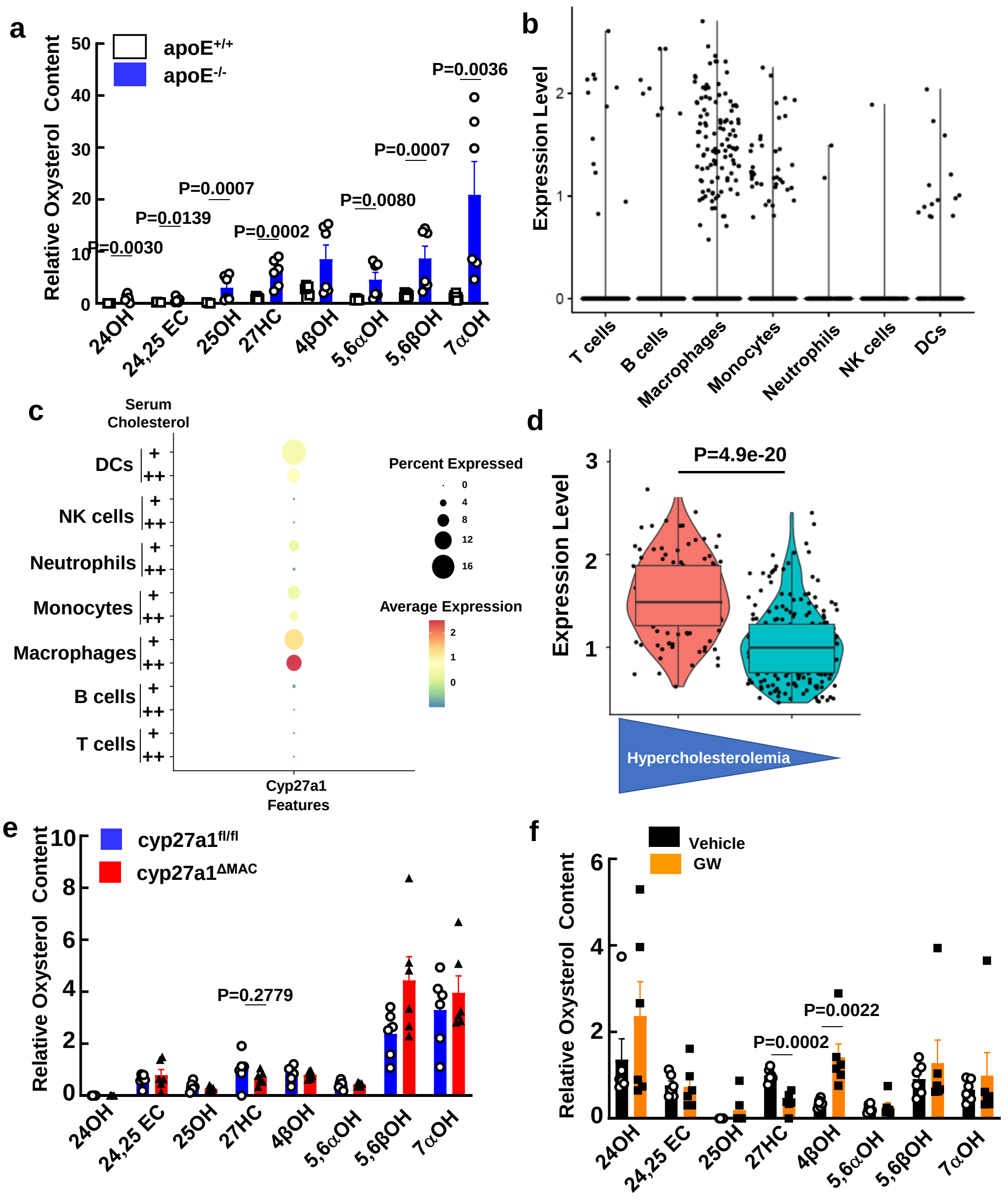

Supplementary Fig. 1. Aorta oxysterol content with hypercholesterolemia, plaque immune cell cyp27a1 expression with hypercholesterolemia and improved cholesterolemia, and oxysterol content with macrophage cyp27a1 deletion or cyp27a1 inhibitor treatment. a, Oxysterol content was compared in aortas from standard chow-fed apoE$^{+/+}$ mice and from apoE$^{-/-}$ mice fed an atherogenic diet for 8 weeks, n=8,6. The oxysterols quantified were 27HC, 24S-

hydroxycholesterol (24OH), 25-epoxycholesterol (24,25EC), 25-hydroxycholesterol (25OH), 4β-hydroxycholesterol (4βOH), 5,6α-epoxycholesterol (5,6αOH), 5,6β-epoxycholesterol (5,6βOH), and 7α-hydroxycholesterol (7αOH). **b-d**. Single cell RNAseq findings for cyp27a1 expression in plaque immune cells. **b**. Violin plots for cyp27a1 expression in the setting of hypercholesterolemia. **c**. Dot plots for cyp27a1 expression in the setting of greater versus less hypercholesterolemia (following switch to standard chow diet). **d**. Violin plots and overlying box plots for lesion macrophage cyp27a1 expression in the setting of greater versus less hypercholesterolemia. The box plot central horizontal line denotes the median value, the edges represent the upper and lower quartiles, and the vertical line indicates the minimum and maximum values. Each dot represents a *Cyp27a1*-expressing cell, and the distribution of the data is represented by the shape of the violin in the background. The P value by two-sided Wilcoxon rank sum test is shown. **e**, Oxysterol content was compared in aortas from apoE$^{-/-}$ background cyp27a1$^{fl/fl}$ and cyp27a1$^{\Delta MAC}$ mice, n=6. **f**, Oxysterol content was compared in aortas from apoE$^{-/-}$ mice following treatment with vehicle or the cyp27a1 inhibitor GW273297X (GW) for 4 weeks, n=6. Results are expressed relative to 27HC content in the control group for each experiment. In **a**, **e** and **f**, data are mean±SEM, and P values by two-sided Student's t-test are shown. Source data are provided as a Source Data file.

Supplementary Fig. 2

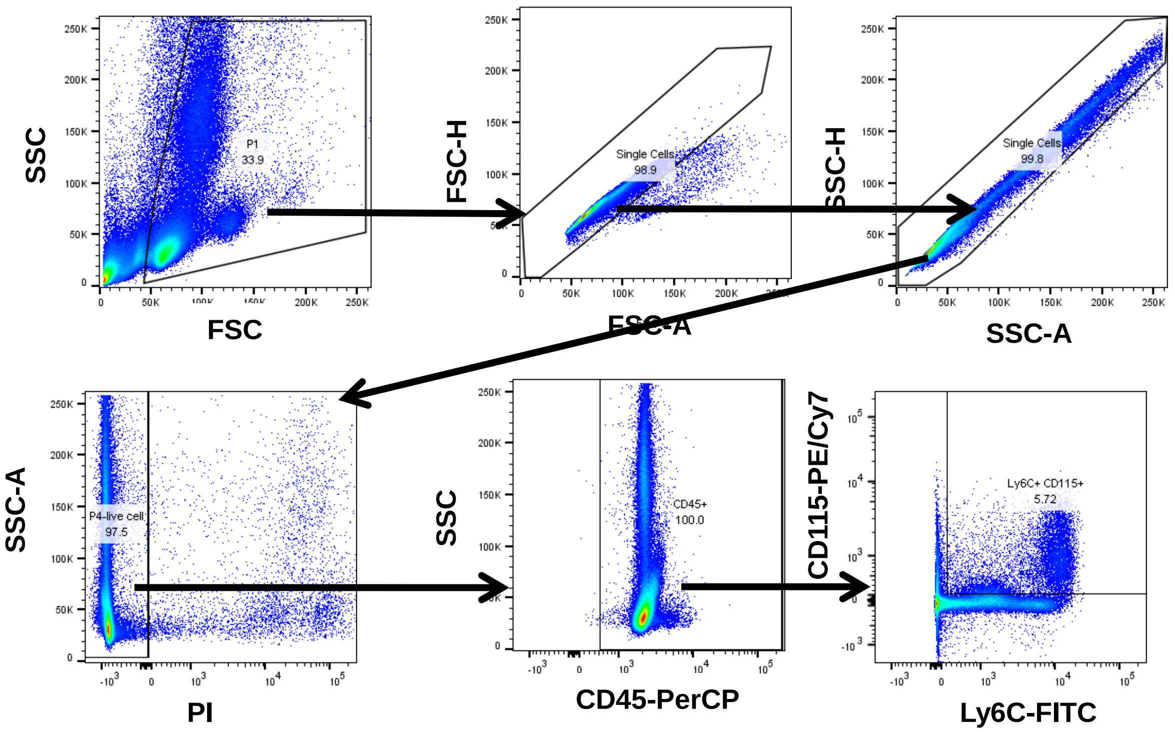

Supplementary Fig. 2. Gating strategy for isolation of circulating monocytes. Following RBC removal, cells were gated in FSC/SSC according to cell size and granularity to identify single cells. The resulting population was gated according to cell viability using propidium iodide (PI). PI negative cells were gated for positivity for CD45, and CD45+ cells were then gated for positivity for CD115 and Ly6C.

Supplementary Fig. 3

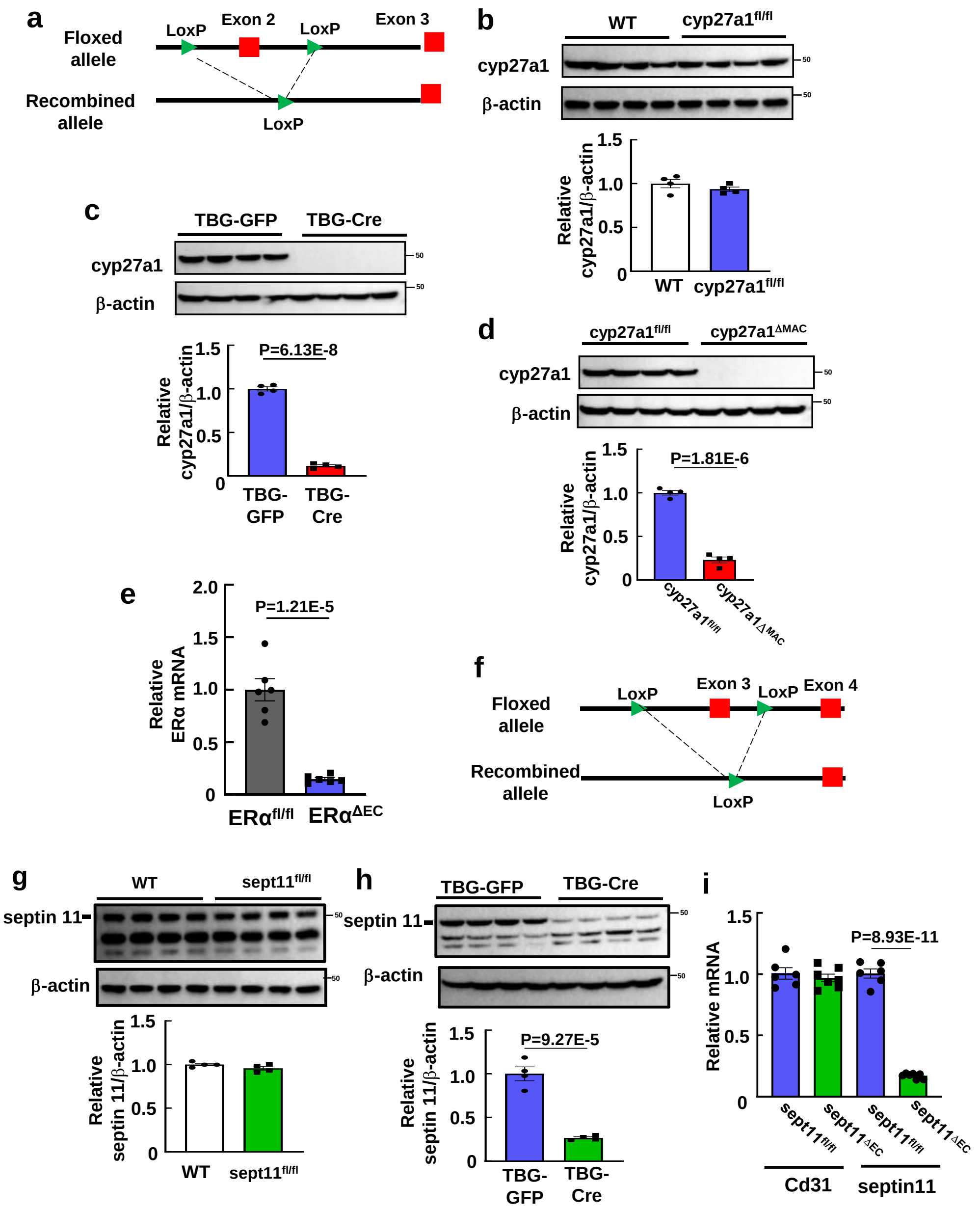

Supplementary Fig. 3. Establishment and characterization of mice lacking macrophage cyp27a1 and mice lacking endothelial ERα or septin 11. a, f, Schematics for floxed allele of cyp27a1 in cyp27a1$^{fl/fl}$ mice (**a**), and floxed allele of septin 11 in sept11$^{fl/fl}$ mice (**f**). The recombined alleles following Cre recombinase introduction are also shown. **b**, Liver cyp27a1

protein abundance in wild type (WT) versus cyp27a1$^{fl/fl}$ mice. Immunoblots for cyp27a1 and β-actin for 4 mice per group are shown in the upper panel, and quantification of cyp27a1 expressed relative to abundance in WT mice is in the lower panel. **c**, Liver cyp27a1 protein abundance 2 weeks following intravenous injection of AAV8 encoding TBG-driven GFP (TBG-GFP) versus TBG-driven Cre recombinase (TBG-Cre) in cyp27a1$^{fl/fl}$ mice. Immunoblots for cyp27a1 and β-actin for 4 mice per group are shown in the upper panel, and quantification of cyp27a1 expressed relative to abundance in TBG-GFP mice is in the lower panel. **d**, Cyp27a1 protein abundance in bone marrow-derived macrophages in cyp27a1$^{fl/fl}$ and cyp27a1$^{\Delta MAC}$ mice. Immunoblots for cyp27a1 and β-actin for 4 mice per group are shown in the upper panel, and quantification of cyp27a1 expressed relative to abundance in cyp27a1$^{fl/fl}$ mice is in the lower panel. **e**. ERα transcript abundance by Q-RT-PCR in endothelial cells isolated from ERα$^{fl/fl}$ and ERα$^{\Delta EC}$ mice. N=6. **g,** Liver septin 11 protein abundance in wild type (WT) versus sept11$^{fl/fl}$ mice. Immunoblots for septin 11 and β-actin for 4 mice per group are shown in the upper panel, and quantification of septin 11 expressed relative to abundance in WT mice is in the lower panel. **h**, Liver cyp27a1 protein abundance 2 weeks following intravenous injection of AAV8 encoding TBG-TBG versus TBG-Cre in sept11$^{fl/fl}$ mice. Immunoblots for sept11 and β-actin for 4 mice per group are shown in the upper panel, and quantification of septin 11 expressed relative to abundance in TBG-GFP mice is in the lower panel. **i**, Septin 11 transcript abundance by Q-RT-PCR in endothelial cells isolated from sept11$^{fl/fl}$ and sept11$^{\Delta EC}$ mice. CD31 mRNA abundance was evaluated to compare the relative yield of endothelial cells, and findings are expressed relative to abundance in cells from sept11$^{fl/fl}$ mice. N=6,7,6,7. Data are mean±SEM, P values by two-sided Student's t-test are shown. Source data are provided as a Source Data file.

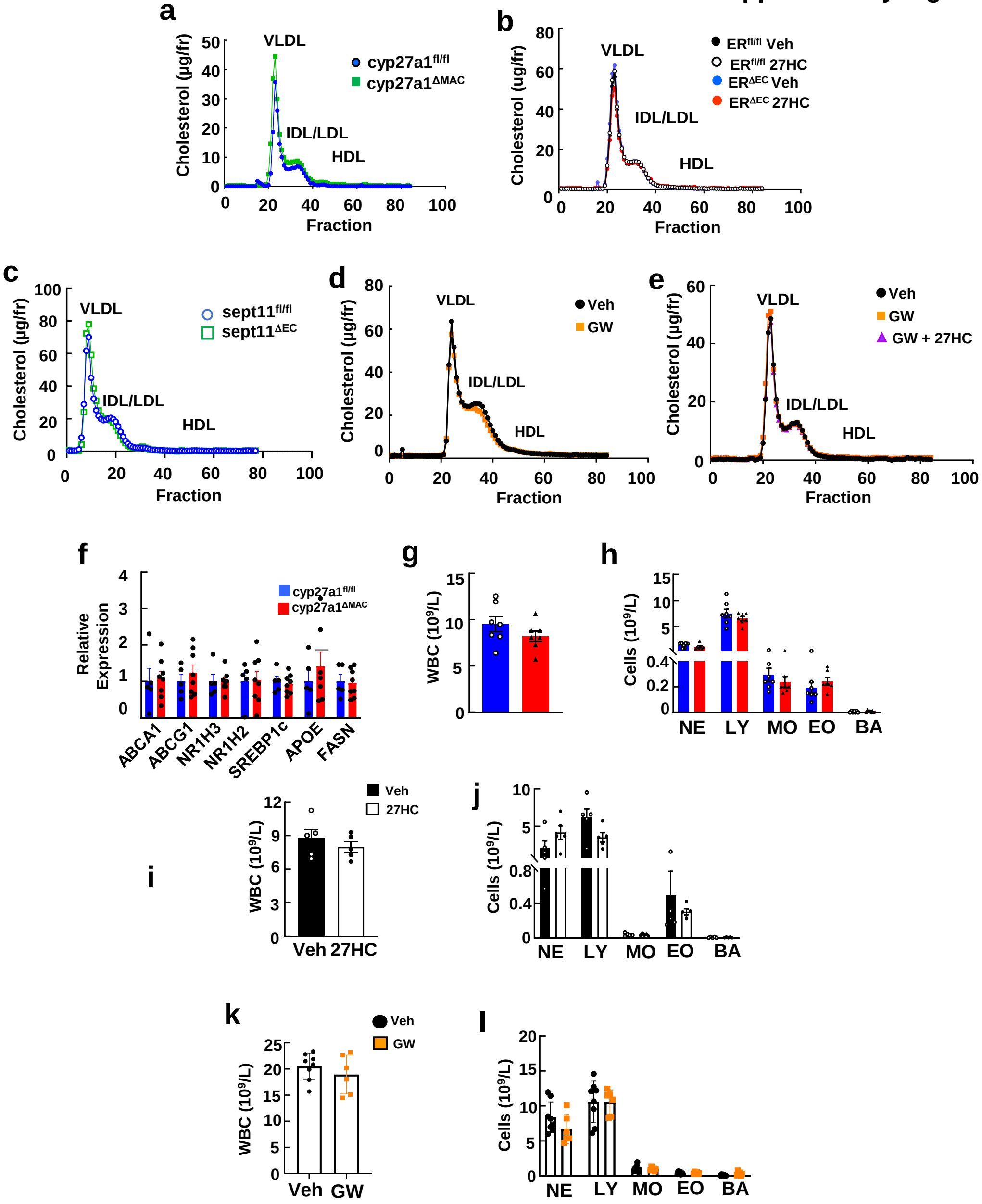

Supplementary Fig. 4. Effects of 27HC manipulation and endothelial ERα or septin 11 deletion on lipoprotein profile and macrophages and circulating leukocytes. Representative lipoprotein profiles are shown for apoE$^{-/-}$ background mice on atherogenic diet for 8 weeks in the following groups: **a**, cyp27a1$^{fl/fl}$ versus cyp27a1$^{\Delta MAC}$ mice, **b**, vehicle versus 27HC administration to ERα$^{fl/fl}$ versus ERα$^{\Delta EC}$ mice, **c**, sept11$^{fl/fl}$ versus sept11$^{\Delta EC}$ mice; **d**, vehicle versus GW273297X

(GW) administration; and **e**, vehicle versus GW versus GW plus 27HC administration. **f,** LXR target gene expression was evaluated in peritoneal macrophages from apoE$^{-/-}$ background cyp27a1$^{fl/fl}$ versus cyp27a1$^{\Delta MAC}$ mice fed an atherogenic diet (n=5 and 8). **g, h**, Peripheral white blood cell counts (**g**) and differential cell counts (**h**) were determined in the same mice (n=7). Leukocytes evaluted were neutrophils (NE), lymphocytes (LY), monocytes (MO), eosinophils (EO) and basophils (BA). **i, j**, The effect of 27HC administration on peripheral white blood cell count (**i**) and differential cell count (**j**) was determined in apoE$^{-/-}$ mice fed an atherogenic diet administered vehicle or 27HC (n=5). **k, l**, The effect of vehicle versus GW273297X (GW) administration on peripheral white blood cell count (**k**) and differential cell count (**l**) was determined in apoE$^{-/-}$ mice fed an atherogenic diet (n=8,6). Data are mean±SEM. Source data are provided as a Source Data file.

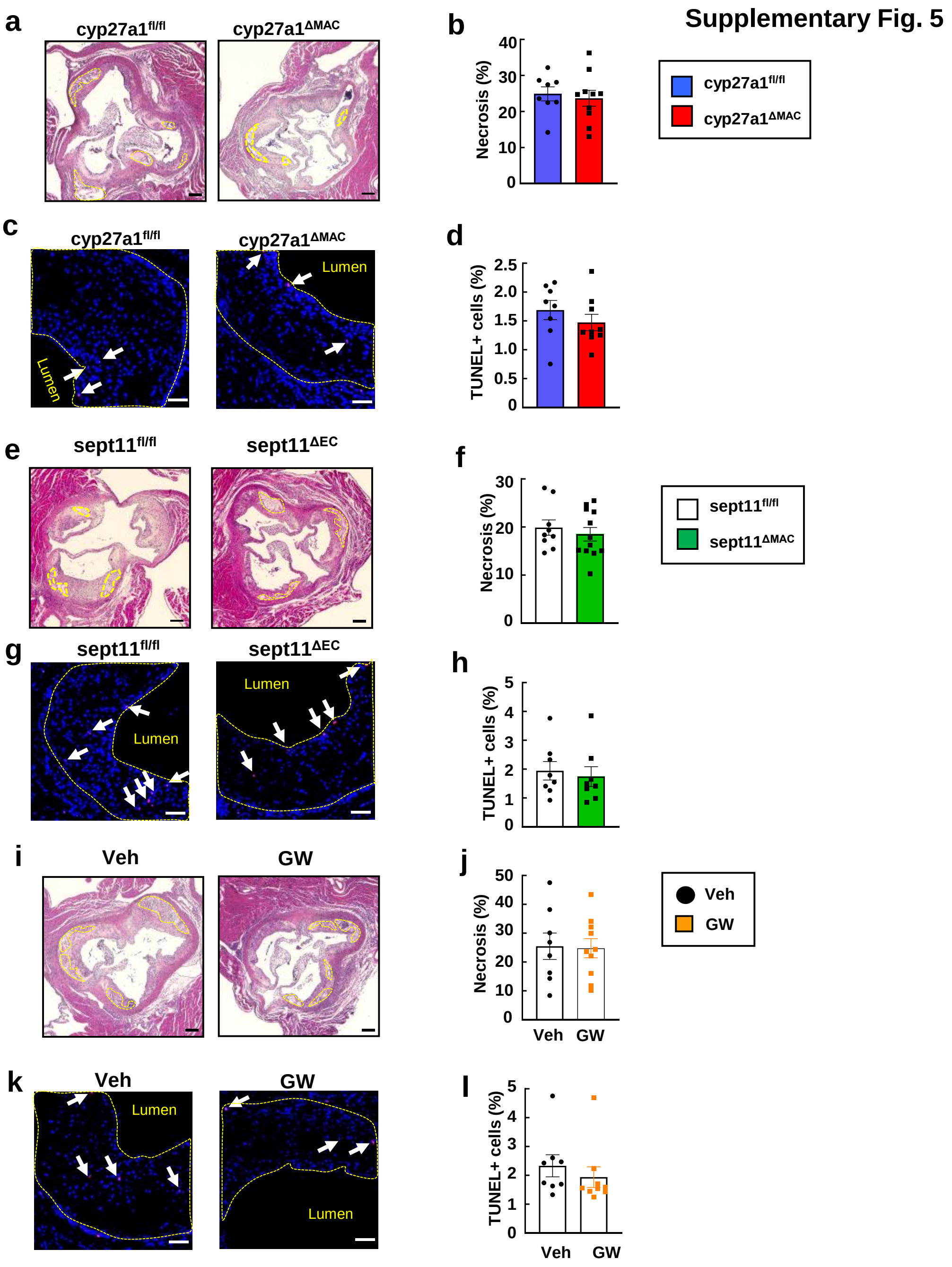

Supplementary Fig. 5. **Effects of 27HC manipulation and endothelial septin 11 deletion on atherogenic lesion necrotic area and apoptosis.** Representative images and quantification are shown for necrotic area and TUNEL staining of atherosclerotic lesions in apoE$^{-/-}$ background mice on atherogenic diet in the following groups: **a-d**, cyp27a1$^{fl/fl}$ versus cyp27a1$^{\Delta MAC}$ (in **b** and **d**,

n=8,10 and n=8,9, respectively); **e-h**, sept11$^{fl/fl}$ versus sept11$^{\Delta EC}$ (in **f** and **h**, n=9,12 and n=8, respectively); and **i-l**, vehicle versus GW273297X (GW) administration (in **j** and **l**, n=8,10 and n=8,9, respectively). In the representative images in **a**, **e**, and **i** necrotic areas are outlined by yellow hatched lines, and in **c**, **g**, and **k** the lesion areas are outlined and the arrows indicate TUNEL+ cells. Scale bar equals 100um (**a, e, i**) or 50 um (**c, g, k**). Data are mean±SEM. Source data are provided as a Source Data file.

Supplementary Fig. 6

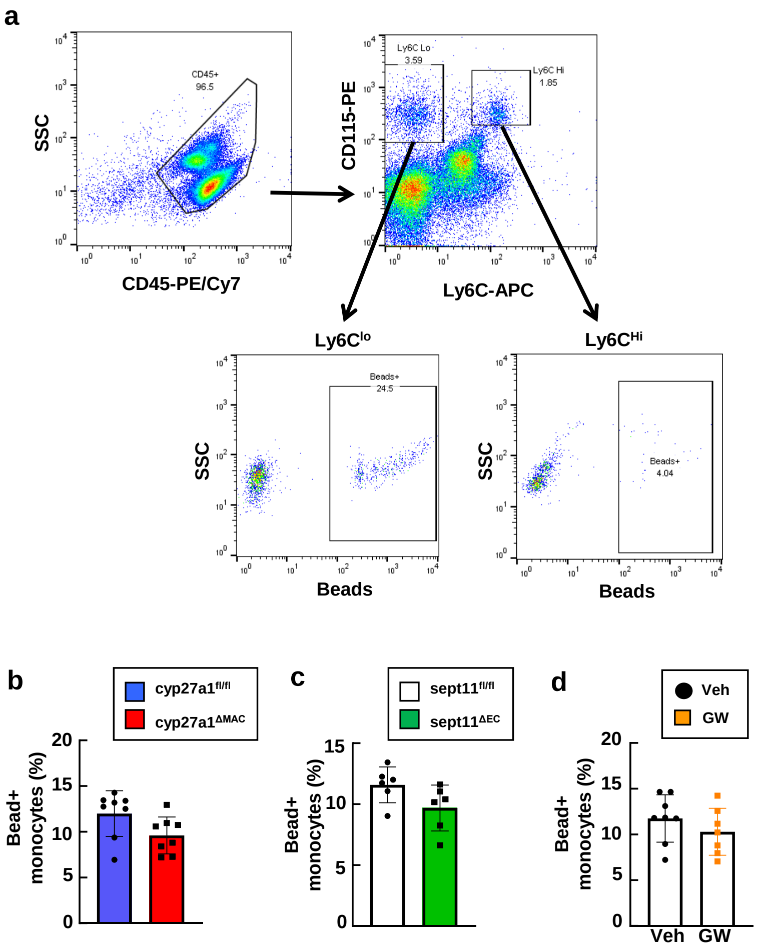

Supplementary Fig. 6. Determination of efficiency of monocyte labeling in studies of atherosclerotic lesion monocyte recruitment. Mice on apoE$^{-/-}$ background were injected intravenously with green fluorescent (YG) microspheres, and 24h later blood was obtained to evaluate monocyte YG bead incorporation by flow cytometry. **a**, Gating strategy. Cells were gated for positivity for CD45, and CD45+ cells were then gated for positivity for CD115 and Ly6C. CD115+ cells determined to be Ly6C^{lo} or Ly6C^{hi} were then gated for positivity for the YG beads. Using the approach outlined in **a**, monocyte labeling efficiency was evaluated in apoE$^{-/-}$ background cyp27a1$^{fl/fl}$ versus cyp27a1$^{\Delta MAC}$ (**b**, n=8), sept11$^{fl/fl}$ versus sept11$^{\Delta EC}$ (**c**, n=6), and vehicle versus GW273297X (GW) treated mice (**d**, n=8,7). Data are mean±SEM. Source data are provided as a Source Data file.

Supplementary Fig. 7

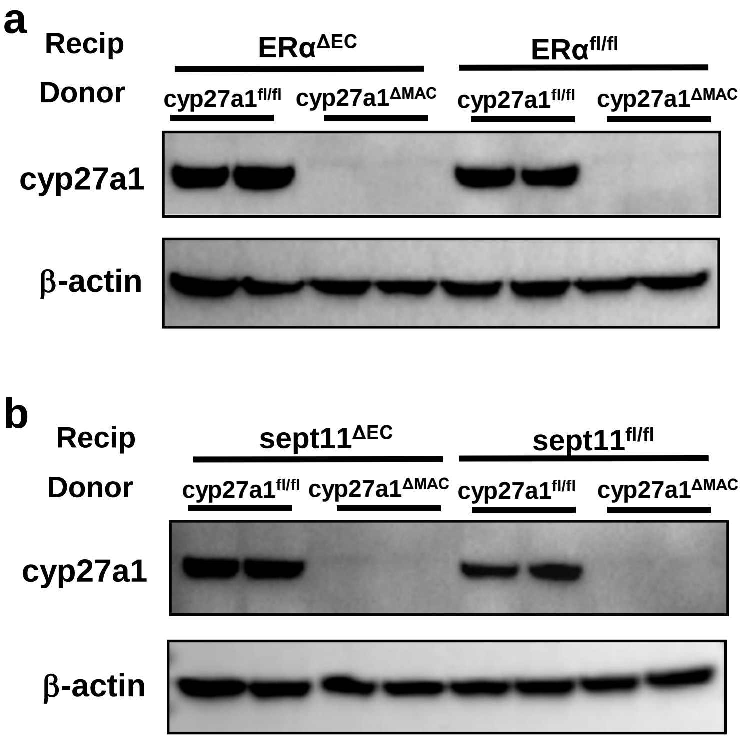

Supplementary Fig. 7. Evaluations of bone marrow reconstitution following bone marrow transplant. Studies were performed employing control cyp27a1$^{fl/fl}$ versus cyp27a1$^{\Delta MAC}$ donors and either ERα$^{fl/fl}$ versus ERα$^{\Delta EC}$ recipients (**a**) or sept11$^{fl/fl}$ versus sept11$^{\Delta EC}$ recipients (**b**), all on apoE$^{-/-}$ background. After 8 weeks of atherosclerotic diet intake begun 2 weeks post-transplant, bone marrow-derived macrophages were obtained and immunoblotting was performed for cyp27a1 and β-actin. Findings for 2 mice per group are shown.

Supplementary Fig. 8

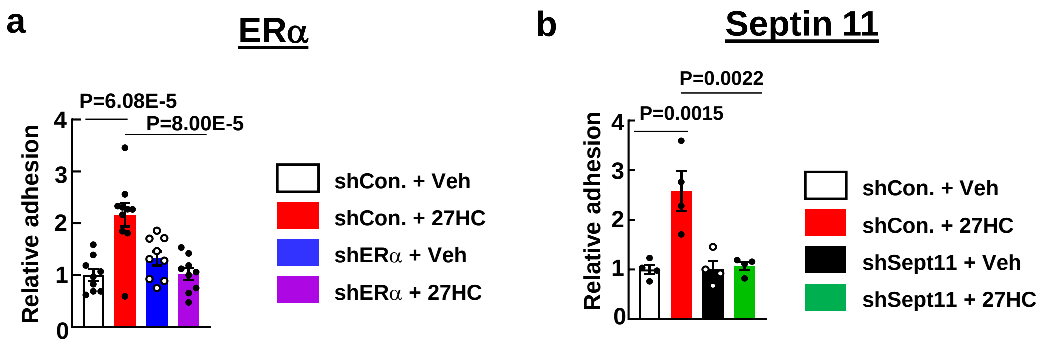

Supplementary Fig. 8. 27HC activation of monocyte-endothelial cell adhesion is mediated by ERα and septin 11. Forty-eight hours following transduction of HAEC with control shRNA or shRNA targeting ERα (**a**, n=9,10,9,9) or septin 11 (**b**, n=4), the effects of treatment with vehicle versus 27HC (20uM for 16h) on monocyte-endothelial cell adhesion were evaluated. Data are mean±SEM, P values by ANOVA with Tukey's post-hoc testing are shown. Source data are provided as a Source Data file.

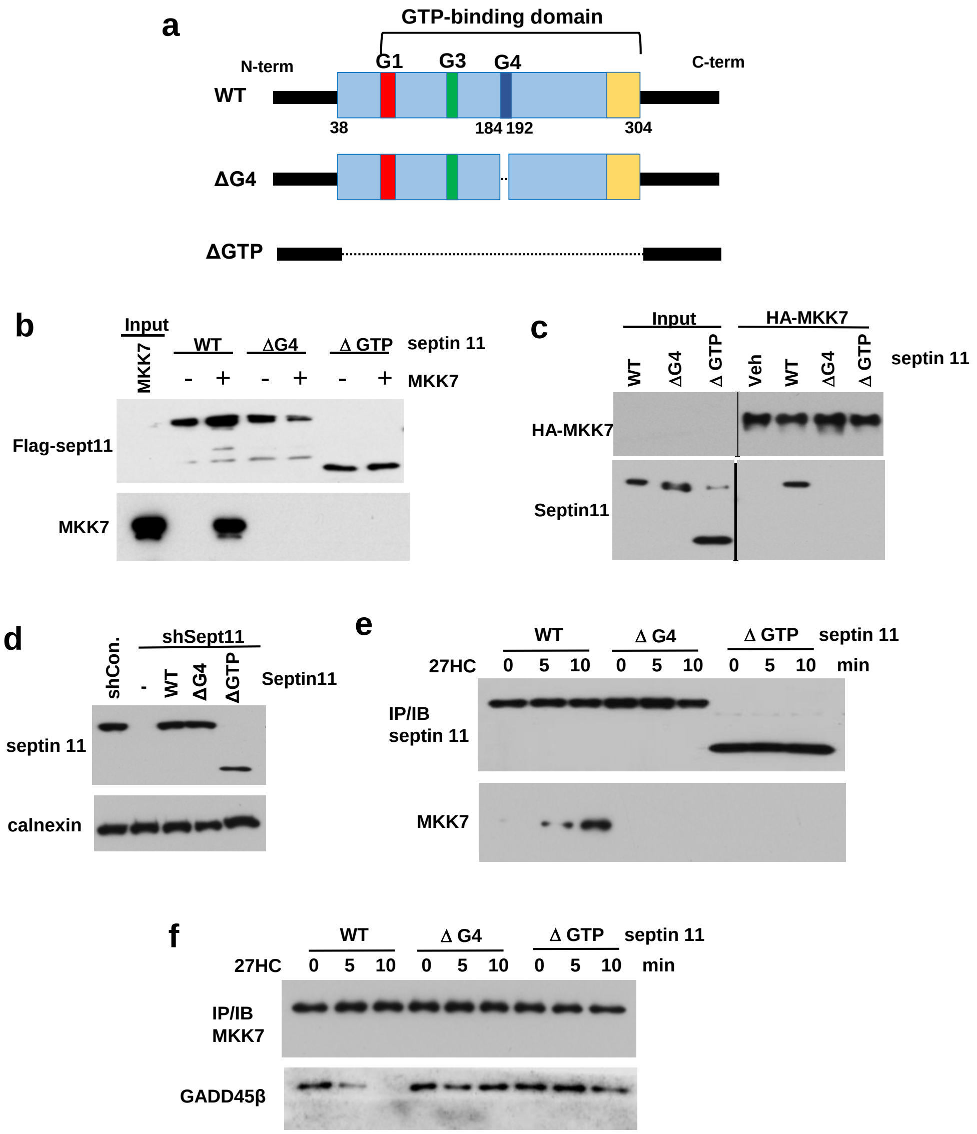

Supplementary Fig. 9. Septin 11 interaction with MKK7 is required for 27HC to cause GADD45β release from MKK7 in intact endothelial cells. **a-c**, Flag-tagged wild-type and mutant forms of septin 11, and HA-tagged MKK7 were generated and purified, and pull-down experiments were performed. **a**, Schematic of wild-type septin 11 (WT) depicting the N-terminal and C-terminal domains and the GTP-binding domain, which includes three common GTP-binding motifs (G1, G3 and G4). Schematics are also provided for deletion mutants lacking AA184-192 (septin 11-ΔG4) or AA38-304 (septin 11-ΔGTP). **b**, Pull-downs in which flag-tagged septin 11

WT or mutant proteins were bound, vehicle or MKK7 was added, and interactions were evaluated by immunoblotting. **c**, HA-tagged MKK7 was bound, vehicle or WT versus mutant septin 11 proteins were added, and interactions were evaluated by immunoblotting. **d-f**, Endogenous septin 11 was silenced in HAEC using shRNA, and reconstitution was performed with either WT septin 11, septin 11-ΔG4 or septin 11-ΔGTP (**d**). Cells were treated with 27HC for 0 to 10 min, septin 11 was IP'd, and immunoblotting was performed for MKK7 and septin 11 (**e**), or MKK7 was IP'd and immunoblotting was done for MKK7 and GADD45β (**f**). The findings in **b-f** were confirmed in two independent experiments.

Supplementary Fig. 10

a

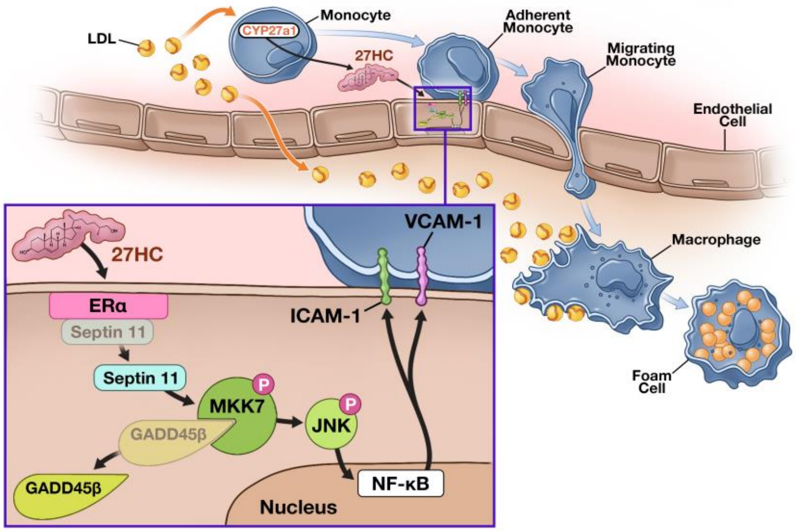

b

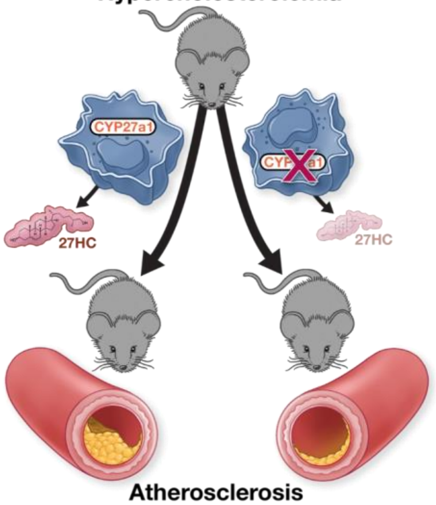

Supplementary Fig. 10. Summary schematics. a, Hypercholesterolemia-driven endothelial activation, monocyte recruitment, foam cell formation and lesion development are promoted by a previously unidentified crosstalk between monocytes/macrophages and endothelial cells mediated by the cholesterol metabolite 27HC. Monocyte/macrophage-derived 27HC binds to endothelial cell ERα, resulting in the disassociation of the cytosolic adaptor protein septin 11 from ERα, septin

11 recruitment to MKK7 leading to release of the inhibitory protein GADD45β, MKK7 activation which activates Jnk, and Jnk-dependent activation of NF-κB that results in the upregulation of ICAM-1 and VCAM-1 expression. **b**, In the setting of hypercholesterolemia, the pharmacologic inhibition of cyp27a1 in monocytes/macrophages is antiatherogenic.

Supplementary Fig. 3

b

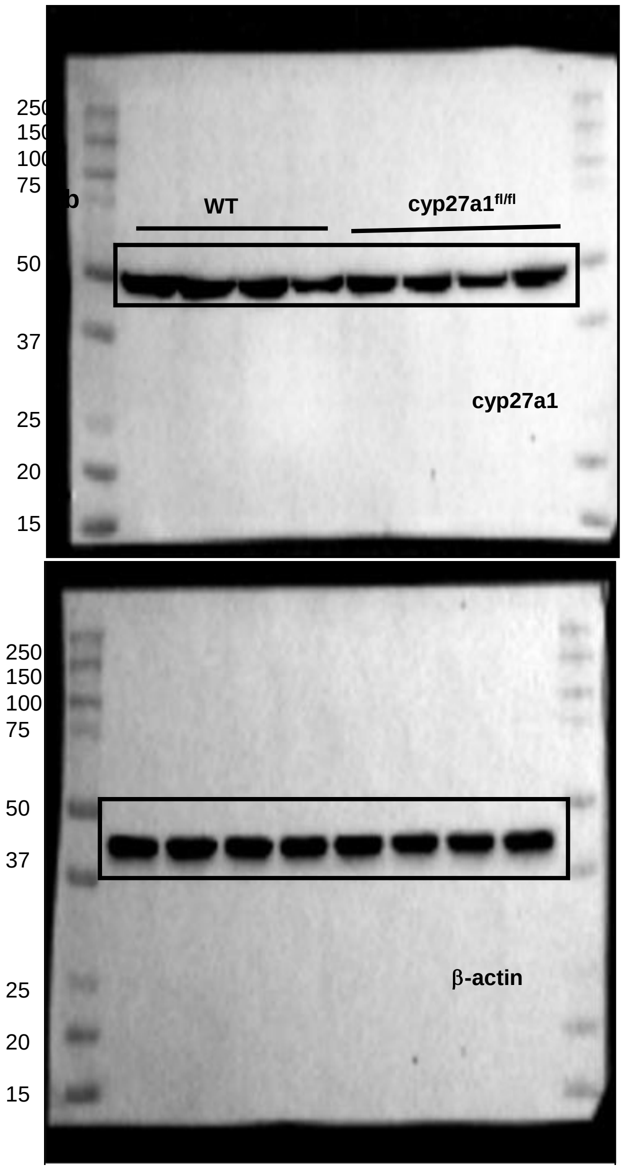

c

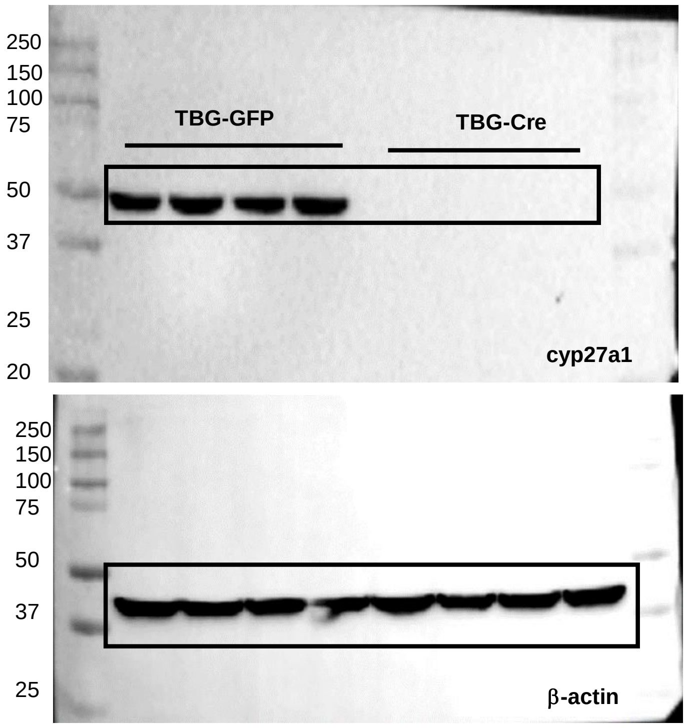

d

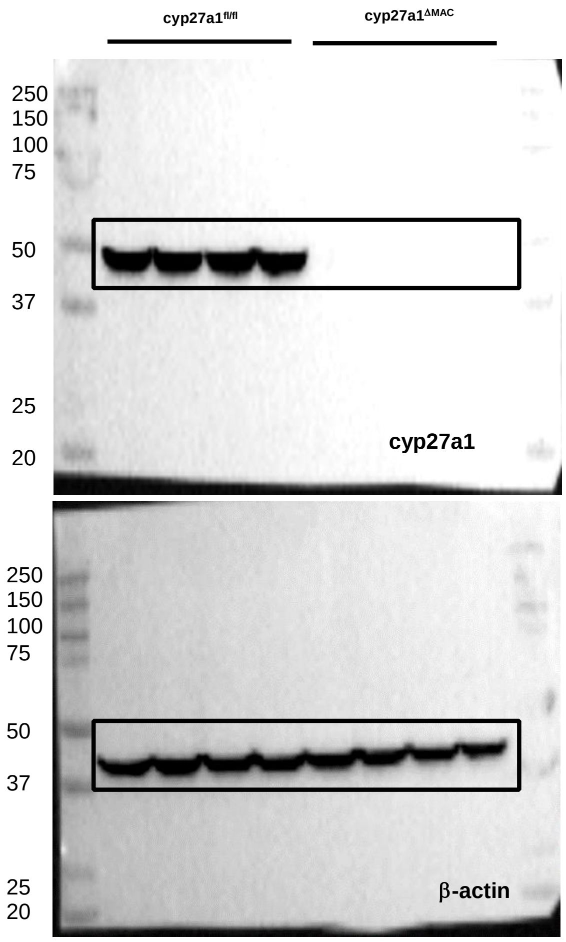

Supplementary Fig. 3

g

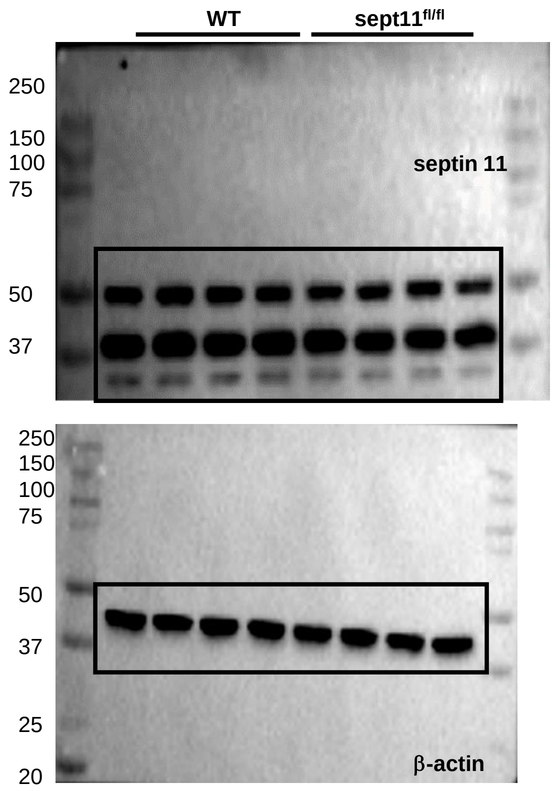

h

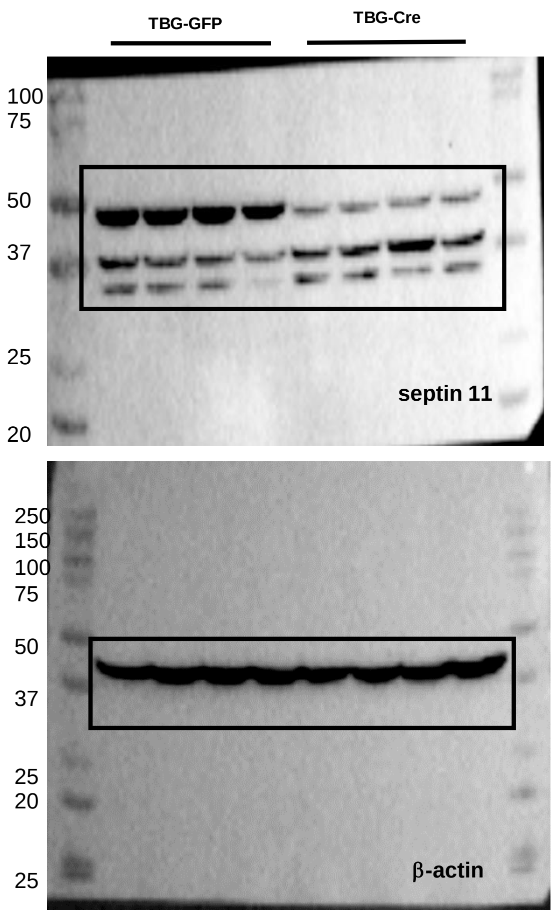

Supplementary Fig. 7

a

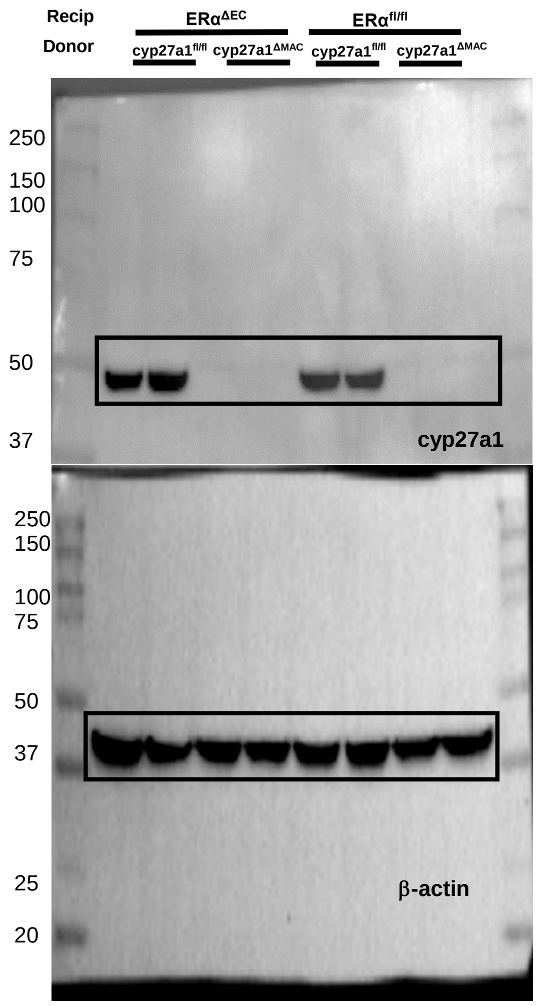

b

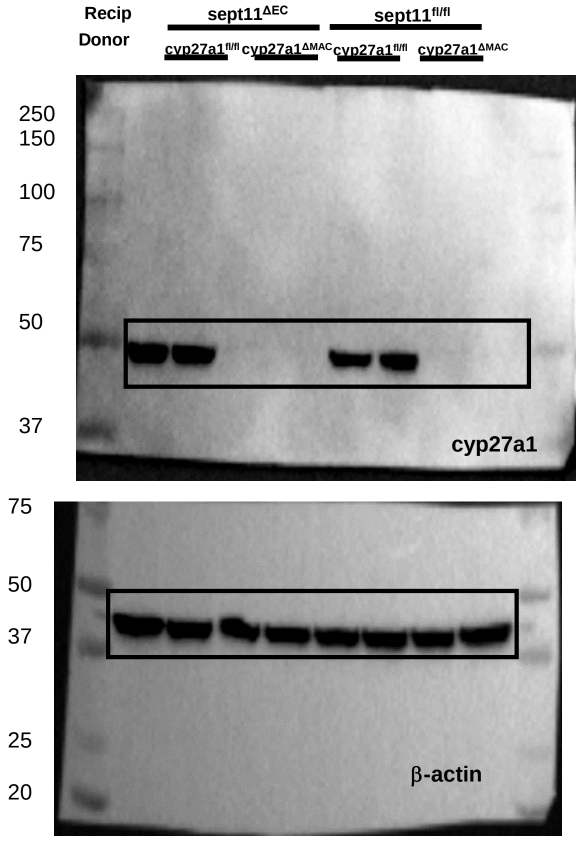

Supplementary Fig. 9

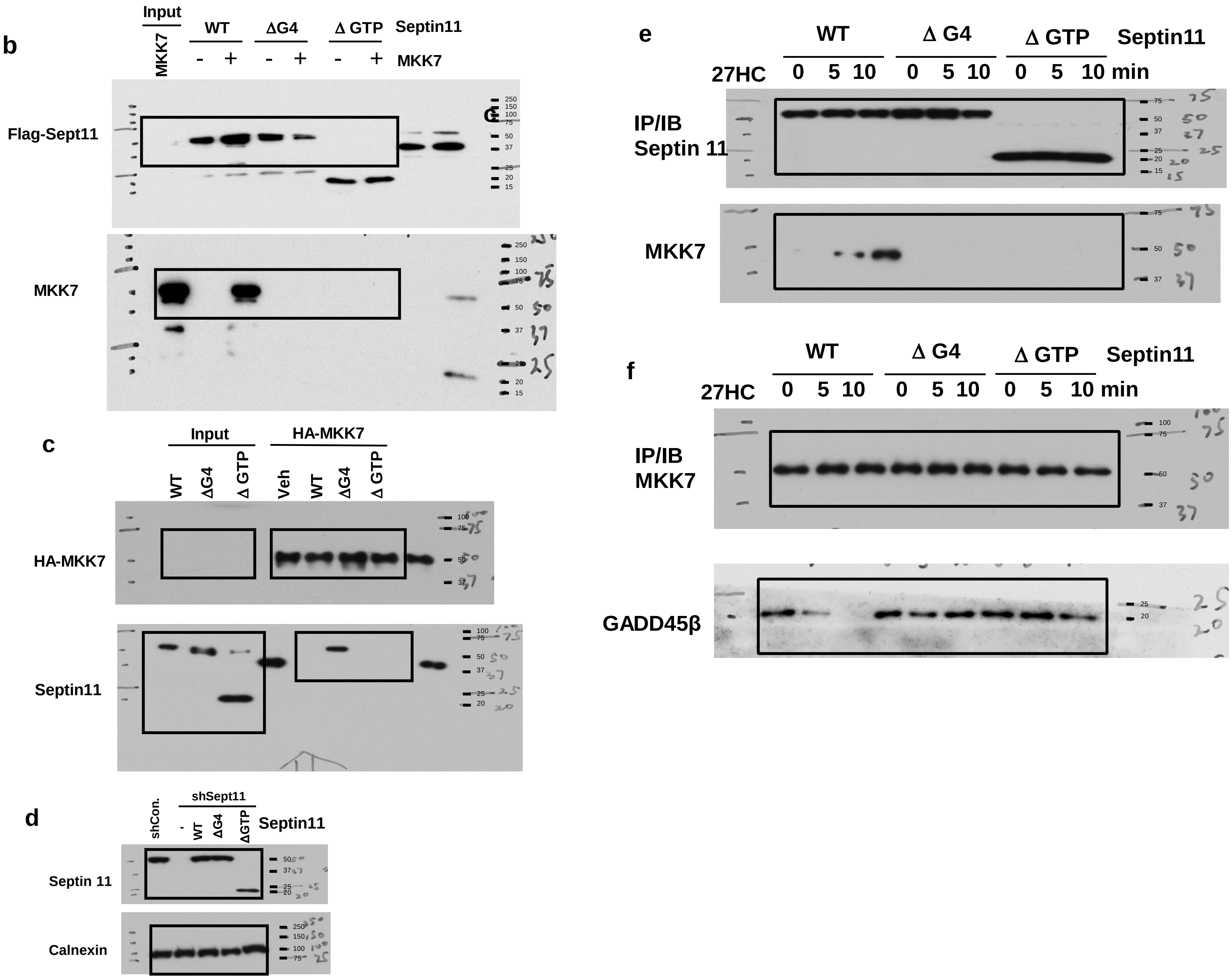

Supplement: Supplementary file 1 — Supplementary Information [file 41467_2023_39586_MOESM1_ESM.pdf]
