# Supplementary material for: Macrophage-to-endothelial cell crosstalk by the cholesterol metabolite 27HC promotes atherosclerosis in male mice
Source: Nat Commun. 2023 Jul 25;14:4101. doi: 10.1038/s41467-023-39586-z (PMC10368733; doi:10.1038/s41467-023-39586-z)
Supplement: Supplementary file 3 — Description of Additional Supplementary Files [file 41467_2023_39586_MOESM3_ESM.pdf]

Description of Additional Supplementary Files

Supplementary Movie 1. Intravital microscopy of leukocyte-endothelial cell adhesion in the mesenteric microcirculation of apoE-/- background cyp27a1 fl/fl mice. 4 week-old male mice were injected with 100 ul rhodamine-6G (0.05% w/v) via the optic vascular plexus to label leukocytes. Under anesthesia, the mesentery was exposed on a clear dish, and images of leukocyte adhesion and rolling in the mesenteric microvasculature were recorded.

Supplementary Movie 2. Intravital microscopy of leukocyte-endothelial cell adhesion in the mesenteric microcirculation of apoE-/- background cyp27a1 ΔMAC mice. Using the approach described for Supplementary Movie 1, images of leukocyte adhesion and rolling in the mesenteric microvasculature were recorded.

Supplementary Movie 3. Intravital microscopy of leukocyte-endothelial cell adhesion in the mesenteric microcirculation of ERαfl/fl control mice administered vehicle. 4 week-old male mice received daily subcutaneous injections of vehicle for 3 days. On day 4 leukocytes were labeled by the administration of rhodamine-6G, and images of leukocyte adhesion and rolling in the mesenteric microvasculature were recorded.

Supplementary Movie 4. Intravital microscopy of leukocyte-endothelial cell adhesion in the mesenteric microcirculation of ERαfl/fl control mice administered 2 27HC. 4 week-old male mice received daily subcutaneous injections of 27HC for 3 days. On day 4 leukocytes were labeled by the administration of rhodamine-6G, and images of leukocyte adhesion and rolling in the mesenteric microvasculature were recorded.

Supplementary Movie 5. Intravital microscopy of leukocyte-endothelial cell adhesion in the mesenteric microcirculation of mice selectively deficient in ERα in endothelial cells (ERαΔEC) administered vehicle. 4 week-old male mice received daily subcutaneous injections of vehicle for 3 days. On day 4 leukocytes were labeled by the administration of rhodamine-6G, and images of leukocyte adhesion and rolling in the mesenteric microvasculature were recorded.

Supplementary Movie 6. Intravital microscopy of leukocyte-endothelial cell adhesion in the mesenteric microcirculation of ERαΔEC mice administered 27HC. 4 week-old male mice received daily subcutaneous injections of 27HC for 3 days. On day 4 leukocytes were labeled by the administration of rhodamine-6G, and images of leukocyte adhesion and rolling in the mesenteric microvasculature were recorded.
