# Supplementary material for: Macrophage-to-endothelial cell crosstalk by the cholesterol metabolite 27HC promotes atherosclerosis in male mice
Source: Nat Commun. 2023 Jul 25;14:4101. doi: 10.1038/s41467-023-39586-z (PMC10368733; doi:10.1038/s41467-023-39586-z)
Supplement: Supplementary file 10 — Reporting Summary [file 41467_2023_39586_MOESM10_ESM.pdf]

nature portfolio

Corresponding author(s): Linzhang Huang and Philip W. Shaul

Last updated by author(s): Jun 9, 2023

Reporting Summary

Nature Portfolio wishes to improve the reproducibility of the work that we publish. This form provides structure for consistency and transparency in reporting. For further information on Nature Portfolio policies, see our Editorial Policies and the Editorial Policy Checklist.

Statistics

For all statistical analyses, confirm that the following items are present in the figure legend, table legend, main text, or Methods section.

n/a	Confirmed	
☐	☒	The exact sample size (*n*) for each experimental group/condition, given as a discrete number and unit of measurement
☒	☐	A statement on whether measurements were taken from distinct samples or whether the same sample was measured repeatedly
☐	☒	The statistical test(s) used AND whether they are one- or two-sided<br>*Only common tests should be described solely by name; describe more complex techniques in the Methods section.*
☒	☐	A description of all covariates tested
☐	☒	A description of any assumptions or corrections, such as tests of normality and adjustment for multiple comparisons
☐	☒	A full description of the statistical parameters including central tendency (e.g. means) or other basic estimates (e.g. regression coefficient) AND variation (e.g. standard deviation) or associated estimates of uncertainty (e.g. confidence intervals)
☐	☒	For null hypothesis testing, the test statistic (e.g. *F*, *t*, *r*) with confidence intervals, effect sizes, degrees of freedom and *P* value noted<br>*Give P values as exact values whenever suitable.*
☒	☐	For Bayesian analysis, information on the choice of priors and Markov chain Monte Carlo settings
☒	☐	For hierarchical and complex designs, identification of the appropriate level for tests and full reporting of outcomes
☒	☐	Estimates of effect sizes (e.g. Cohen's *d*, Pearson's *r*), indicating how they were calculated

Our web collection on statistics for biologists contains articles on many of the points above.

Software and code

Policy information about availability of computer code

Data collection	Aorta and aortic root atherosclerosis lesions were visualized using a Nikon 3500 digital SLR camera and a BZ-X710 microscope (Keyence), respectively; western blot densitometry data by Epson Scan ver.3.9.2.1 (Seiko Epson Corp.) or Bio-Rad ChemiDoc XRS Imaging System; leukocyte adhesion and rolling using a Regita digital camera; circulating monocyte sorting and monocyte YG-beads labeling efficiency were done using a BD FACS Aria II SORP cell sorter and BD FACSCalibur™ Flow Cytometer, respectively; qRT-PCR was performed on a QuantStudio Real-Time PCR System; and immunofluorescence images were captured using a LSM 880 confocal microscope with Airyscan.
Data analysis	Western blot band quantification and image analyses, including aortic root section Mac2 content, necrotic area, TUNEL staining, and adhesion molecule abundance were done by Image J version 1.53c or Fiji-open source software; flow cytometry data analysis was performed using FlowJo v10.7.2; confocal microscopy data analysis was by ZEN Blue Edition software; leukocyte adhesion and rolling data analysis using Image-Pro V.6.2 (Media Cybernetics); single-cell analyses were carried out using the Seurat version 4.3.0 package in R version 4.2.0; statistical analyses were performed by Prism version 8 or higher (GraphPad Inc.).

For manuscripts utilizing custom algorithms or software that are central to the research but not yet described in published literature, software must be made available to editors and reviewers. We strongly encourage code deposition in a community repository (e.g. GitHub). See the Nature Portfolio guidelines for submitting code & software for further information.

Data

Policy information about availability of data

All manuscripts must include a data availability statement. This statement should provide the following information, where applicable:

- Accession codes, unique identifiers, or web links for publicly available datasets
- A description of any restrictions on data availability
- For clinical datasets or third party data, please ensure that the statement adheres to our policy

The raw LC/MS-MS data in this study has been uploaded to the MassIVE data repository with accession number MSV000091880 at https://massive.ucsd.edu/ProteoSAFe/dataset.jsp?task=aba429f8538a465a85a530f075ec7287. The publicly available scRNA-seq data re-analyzed in this study has been deposited in the Gene Expression Omnibus database under the accession codes GSE14103816, GSE16149417 and GSE16838918 at https://www.ncbi.nlm.nih.gov/geo/query/acc.cgi?acc=GSE141038, https://www.ncbi.nlm.nih.gov/geo/query/acc.cgi?acc=GSE161494 and https://www.ncbi.nlm.nih.gov/geo/query/acc.cgi?acc=GSE168389. Source data are provided with this paper.

Human research participants

Policy information about studies involving human research participants and Sex and Gender in Research.

Reporting on sex and gender	Not applicable.
Population characteristics	Not applicable.
Recruitment	Not applicable.
Ethics oversight	Not applicable.

Note that full information on the approval of the study protocol must also be provided in the manuscript.

Field-specific reporting

Please select the one below that is the best fit for your research. If you are not sure, read the appropriate sections before making your selection.

☒ Life sciences ☐ Behavioural & social sciences ☐ Ecological, evolutionary & environmental sciences

For a reference copy of the document with all sections, see nature.com/documents/nr-reporting-summary-flat.pdf

Life sciences study design

All studies must disclose on these points even when the disclosure is negative.

Sample size	No power calculations were performed to predetermine sample size. For evaluating atherosclerotic lesion development and related phenotypes in mice, sample sizes were chosen based on experience with previous studies which indicated that differences in lesion severity can be discerned with n=8-10 per group (PMID: 24954418). It should be noted that group size is impacted by the number of mice born of the desired sex in the desired genetic groups in the cohorts. The number of independent mice employed is apparent in the data and listed in the figure legend. In experiments in cell culture power analysis was not performed, and sample sizes were chosen based on experience with previous studies which indicated that n=6 per group is most often sufficient to discern differences in endpoints between study groups (PMID: 24954418)
Data exclusions	No data were excluded from data analysis.
Replication	With the exception of the bone marrow transplant experiments, the findings in atherosclerosis studies were replicated in 2 separate cohorts. The bone marrow transplant studies were not replicated due to ethical and financial limitations as they required large numbers of mice. Findings in cell culture studies were replicated in at least 3 independent experiments.
Randomization	Within all genotype groups of mice, animals were randomly assigned to the experimental groups. In cell culture experiments, wells or plates were randomly assigned to study groups.
Blinding	The investigators were not blinded to experimental group assignment in the mouse experiments because the group differences were genetically-based and not within the control of the investigator. In the cell culture experiments blinding was not possible because treatments by the investigator were required, such as incubation of cells with vehicle versus 27HC.

Reporting for specific materials, systems and methods

We require information from authors about some types of materials, experimental systems and methods used in many studies. Here, indicate whether each material, system or method listed is relevant to your study. If you are not sure if a list item applies to your research, read the appropriate section before selecting a response.

Materials & experimental systems

n/a	Involved in the study
☐	☒ Antibodies
☐	☒ Eukaryotic cell lines
☒	☐ Palaeontology and archaeology
☐	☒ Animals and other organisms
☒	☐ Clinical data
☒	☐ Dual use research of concern

Methods

n/a	Involved in the study
☒	☐ ChIP-seq
☐	☒ Flow cytometry
☒	☐ MRI-based neuroimaging

Antibodies

Antibodies used

Cyp27a1 (ab126785, Abcam), calnexin (ADI-SPA-860-F, Enzo Life Sciences, Inc.), CD31 (BD, 550274), Mac2 (Cedarlane, CL8942AP), ICAM-1 (R&D, AF796), VCAM-1 (R&D, AF643), CD31 (Abcam, ab28364), donkey anti-rat conjugated to Alexa 488 (Thermo Fisher Scientific, A-21208), donkey anti-goat conjugated to Alexa 647 (Thermo Fisher Scientific, A-21447), and donkey anti-rabbit conjugated to Alexa 488 (Thermo Fisher Scientific, A-21206). CD45 (CD45: Phycoerythrin (PE)-Cy7, BD, 561868, Clone 30-F11), CD115 (CD115-PE, BD, 566839, Clone AFS98), Ly6C (Ly6C-APC, BD, 560595, Clone AL-21), ERalpha (sc-543, Santa Cruz; ab32063, Abcam), phospho-SAPK/JNK (Thr183/Tyr185) (9251, Cell Signaling Tech), SAPK/JNK (9252, Cell Signaling Tech), JNK1(3708, Cell Signaling Tech), phospho-MKK7 (Ser271/Thr275; 4171, Cell Signaling Tech), MKK7 (4172, Cell Signaling Tech), phospho-MLK3 (Thr277/Ser281) (ab191530, Abcam), MLK3 (2817, Cell Signaling Tech), GAPDH (sc-365062, Santa Cruz), mouse CD16/32 antibody (Biolegend, 101320), CD45 (PerCP, Biolegend, 103130, Clone 30-F11), Ly6C (FITC, Biolgend, 128006, Clone HK1.4), CD115 (PE/Cy7, Biolegend, 135524, Clone AFS98), Septin 11 (ab183529, Abcam), GADD45β (ab205252, Abcam, GADD45β (SAB2108614, Sigma), beta-actin (A1978, Sigma), Flag (F1804, Sigma), HA (H3663, Sigma), Goat anti-Rabbit IgG Antibody, (H+L) HRP conjugate (AP307P, Millipore Sigma), Goat Anti-Mouse IgG Antibody, (H+L) HRP conjugate (AP308P, Millipore Sigma)

Validation

The specificity of the antibodies was provided by the commercial sources as follows:
CD31 https://www.bdbiosciences.com/en-us/products/reagents/flow-cytometry-reagents/research-reagents/single-color-antibodies-ruo/purified-rat-anti-mouse-cd31.550274
Mac2 https://www.cedarlanelabs.com/Products/Detail/CL8942AP?lob=AllProducts
ICAM-1 https://www.rndsystems.com/products/mouse-icam-1-cd54-antibody_af796
VCAM-1 https://www.rndsystems.com/products/mouse-vcam-1-cd106-antibody_af643
CD31 https://www.abcam.com/products/primary-antibodies/cd31-antibody-ab28364.html
donkey anti-rat conjugated to Alexa 488 (Thermo Fisher Scientific, A-21208),
https://www.thermofisher.com/antibody/product/Donkey-anti-Rat-IgG-H-L-Highly-Cross-Adsorbed-Secondary-Antibody-Polyclonal/A-21208
donkey anti-goat conjugated to Alexa 647 (Thermo Fisher Scientific, A-21447),
https://www.thermofisher.com/antibody/product/Donkey-anti-Goat-IgG-H-L-Cross-Adsorbed-Secondary-Antibody-Polyclonal/A-21447
donkey anti-rabbit conjugated to Alexa 488 (Thermo Fisher Scientific, A-21206).
https://www.thermofisher.com/antibody/product/Donkey-anti-Rabbit-IgG-H-L-Highly-Cross-Adsorbed-Secondary-Antibody-Polyclonal/A-21206
CD45 (CD45-PE-Cy7) https://www.bdbiosciences.com/en-us/products/reagents/flow-cytometry-reagents/research-reagents/single-color-antibodies-ruo/pe-cy-7-rat-anti-mouse-cd45.561868
CD115 (CD115-PE) https://www.bdbiosciences.com/en-us/products/reagents/flow-cytometry-reagents/research-reagents/single-color-antibodies-ruo/pe-rat-anti-mouse-cd115-csf-1r.566839
Ly6C (Ly6C-APC) https://www.bdbiosciences.com/en-us/products/reagents/flow-cytometry-reagents/research-reagents/single-color-antibodies-ruo/apc-rat-anti-mouse-ly-6c.560595
ERalpha (sc-543, Santa Cruz; ab32063, Abcam),
https://www.scbt.com/p/eralpha-antibody-hc-20?requestFrom=search
https://www.abcam.com/products/primary-antibodies/estrogen-receptor-alpha-antibody-e115-chip-grade-ab32063.html
phospho-SAPK/JNK (Thr183/Tyr185)
https://www.cellsignal.com/products/primary-antibodies/phospho-sapk-jnk-thr183-tyr185-antibody/9251
SAPK/JNK https://www.cellsignal.com/products/primary-antibodies/sapk-jnk-antibody/9252
JNK1 https://www.cellsignal.com/products/primary-antibodies/jnk1-2c6-mouse-mab/3708
phospho-MKK7 https://www.cellsignal.com/products/primary-antibodies/phospho-mkk7-ser271-thr275-antibody/4171
MKK7 https://www.cellsignal.com/products/primary-antibodies/mkk7-antibody/4172
phospho-MLK3 https://www.abcam.com/products/primary-antibodies/mlk3-phospho-t277--s281-antibody-ab191530.html
MLK3 https://www.cellsignal.com/products/primary-antibodies/mlk3-antibody/2817
Calnexin: https://www.enzolifesciences.com/ADI-SPA-860/calnexin-polyclonal-antibody/
GAPDH https://www.scbt.com/p/gapdh-antibody-g-9
mouse CD16/32 antibody https://www.biolegend.com/en-us/products/trustain-fcx-anti-mouse-cd16-32-antibody-5683
CD45-PerCP https://www.biolegend.com/en-us/products/percp-anti-mouse-cd45-antibody-4265
Ly6C-FITC https://www.biolegend.com/en-us/products/fitc-anti-mouse-ly-6c-antibody-4896

CD115-PE/Cy7 https://www.biolegend.com/en-us/products/pe-cyanine7-anti-mouse-cd115-csf-1r-antibody-12376
Septin 11 https://www.abcam.com/products/primary-antibodies/septin-11-antibody-ab183529.html
Cyp27a1 https://www.abcam.com/products/primary-antibodies/cyp27a1-antibody-epr7529-ab126785.html
GADD45β https://www.abcam.com/products/primary-antibodies/gadd45b-antibody-ab205252.html
GADD45β https://www.sigmaaldrich.com/US/en/product/sigma/sab2108614
Beta-actin https://www.sigmaaldrich.com/US/en/product/sigma/a1978
HA https://www.sigmaaldrich.com/US/en/product/sigma/h3663
Flag https://www.sigmaaldrich.com/US/en/product/sigma/f1804
Goat anti-Rabbit IgG Antibody, (H+L) HRP conjugate https://www.emdmillipore.com/US/en/product/Goat-Anti-Rabbit-IgG-Antibody-HL-HRP-conjugate,MM_NF-AP307P
Goat Anti-Mouse IgG Antibody, (H+L) HRP conjugate
https://www.emdmillipore.com/US/en/product/Goat-Anti-Mouse-IgG-Antibody-HL-HRP-conjugate,MM_NF-AP308P

In some instances we additionally independently validated the specificity of the antibodies used in western blotting by deletion of the protein in cell culture or in vivo. This was done for JNK1 (Fig. 3l), cyp27a1 (Supplementary FIg. 3c,d and Supplementary Fig. 7a,b), and Septin 11 (Supplementary Fig. 3h).
For immunofluorescence, we validated the specificity by performing control studies omitting the primary antibody.

Eukaryotic cell lines

Policy information about cell lines and Sex and Gender in Research

Cell line source(s)	Cell line source- Primary human aortic endothelial cells (HAEC) were purchased from Lonza (cat# CC-2535), and HEK-293FT cells were purchased from ThermoFisher Scientific (Catalog No. R70007)
Authentication	HAEC express endothelial markers CD31/105 and von Willebrand Factor VIII. HAEC used in the studies were also positive for labeled LDL uptake. Homogeneous transcriptome patterns and low variance have recently been demonstrated in HEK-293FT cells in culture conditions using single cell RNA sequencing (PMID: 36708997).
Mycoplasma contamination	The cells were tested negative for mycoplasma, bacteria, yeast and fungi.
Commonly misidentified lines (See ICLAC register)	No commonly misidentified cell lines were used.

Animals and other research organisms

Policy information about studies involving animals; ARRIVE guidelines recommended for reporting animal research, and Sex and Gender in Research

Laboratory animals	The Institutional Animal Care and Use Committee at UT Southwestern Medical Center approved all animal experiments. All mice used for these studies were on a C57BL/6J background and housed in a specific pathogen- free facility at UT Southwestern Medical Center. The mice were housed at 23C with light cycles of 12h of light beginning at 6:00am and 12h of dark beginning at 6:00pm, humidity was 30-70%, and H2O was provided ad libitum.<br>ApoE-/- mice were purchased from the Jackson Laboratory (Strain #002052). ER fl/fl mice were provided by Sohaib Khan (University of Cincinnati Cancer Center). VECad-Cre mice provided by Luisa Iruela-Arispe (UCLA). LysM-Cre mice were purchased from the Jackson Laboratory (Strain #004781). In cyp27a1fl/fl mice loxP sites were inserted into intron 1 and intron 2 of the cyp27a1 gene. In sept11fl/fl mice loxP sites were inserted into intron 2 and intron 3 of the septin 11 gene. For studies of atherosclerosis, mice on apoE-/- background were fed an atherogenic diet (D12108C, 20% fat, 1.25% cholesterol, Research Diets Inc.) for 8 weeks following weaning. Bone marrow transplant experiments were conducted with recipient mice between the age of 6-8 weeks. All donor mice were 6-8 weeks of age. Following transplant the mice were placed on an atherogenic diet for 8 weeks. For studies of cyp27a1 inhibition, the small molecule inhibitor (GX273297X) was synthesized from chenodeoxycholic acid by Sai Life Science, LTD and dissolved in 30% hydroxypropyl-beta-cyclodextrin, with facilitation by dispersion by mortar and pestle and sonication. Vehicle or GX273297X (100 mg/kg body weight) was administered by daily subcutaneous injection during the last 4 weeks of an 8 week period on an atherogenic diet. To administer 27HC to mice, 27HC was purchased from Avanti Polar Lipid Inc. (Alabaster, AL) and dissolved in 30% hydroxypropyl-beta-cyclodextrin (Sigma). Vehicle or 27HC was injected subcutaneously at a dose of 20 mg/kg body weight, either every 2 days for 8 weeks in atherosclerosis studies, or daily for 3 days for in vivo intravital microscopy studies of leukocyte-endothelial cell adhesion or evaluation of the impact on circulating leukocytes.
Wild animals	No wild animals were used.
Reporting on sex	The findings were obtained exclusively in male mice. This was purposeful because estrogens antagonize the actions of 27-hydroxycholesterol, a key aspect of the biology being studied.
Field-collected samples	There were no field-collected samples.
Ethics oversight	The Institutional Animal Care and Use Committee at UT Southwestern Medical Center approved all animal experiments.

Note that full information on the approval of the study protocol must also be provided in the manuscript.

Flow Cytometry

Plots

Confirm that:

☒ The axis labels state the marker and fluorochrome used (e.g. CD4-FITC).

☒ The axis scales are clearly visible. Include numbers along axes only for bottom left plot of group (a 'group' is an analysis of identical markers).

☒ All plots are contour plots with outliers or pseudocolor plots.

☒ A numerical value for number of cells or percentage (with statistics) is provided.

Methodology

Sample preparation	Monocyte recruitment studies- After mice were fed an atherogenic diet for 8 weeks, Ly6Clo monocytes were labeled in vivo by retro-orbital injection with 250ul 1 µm Fluoresbrite green fluorescent (YG) plain microspheres diluted 1:4 in sterile PBS. 24h later, blood was taken for flow cytometry to measure YG-beads incorporation efficiency. Briefly, leukocytes were identified by CD45 staining (CD45: Phycoerythrin (PE)-Cy7, BD, 561868, Clone 30-F11). Ly6Chi monocytes (CD115hiLy6Chi) and Ly6Clo monocytes (CD115hiLy6Clo) were further characterized from monocyte populations that are both CD115 (CD115-PE, BD, 566839, Clone AFS98) positive and Ly6C (Ly6C-APC, BD, 560595, Clone AL-21) positive. Green fluorescence revealed YG-bead abundance.<br>Isolation of circulating monocytes- After mice were fed an atherogenic diet for 4 weeks, following red blood cells removal and washing, samples were washed and blocked with anti-mouse CD16/32 antibody. Ly6C (FITC, Biolegend, 128006, Clone HK1.4) and CD115 (PE/Cy7, Biolegend, 135524, Clone AFS98) double positive monocytes were further characterized from propidium iodide (PI) negative leukocytes identified by CD45 staining (PerCP, Biolegend, 103130, Clone 30-F11). This was accomplished by incubating the cells with CD45/Ly6C/CD155 cocktails for 30 min on ice protected from light before sorting.
Instrument	Samples were analyzed using a BD FACSCalibur™ Flow Cytometer or a BD FACS Aria II SORP cell sorter.
Software	FlowJo-v10.7.2.
Cell population abundance	During the sorting of circulating monocytes, 2-6% of CD115+, Ly6C+ cells were gated from alive CD45+ cells population.<br>In the studies of YG-bead labeled monocytes, about 10% of cells was labeled with YG-bead.
Gating strategy	Unstained cells were used as gating controls. Compensation was performed with single antibody-stained cells and fluorescence minus one (FMO) controls. Single alive cells were first gated in FSC/SSC according to cell size and granularity followed by gating with PI. Subsequently the cells were gated according to positivity or negativity for the specific surface markers being interrogated.

☒ Tick this box to confirm that a figure exemplifying the gating strategy is provided in the Supplementary Information.
