# Supplementary material for: Macrophage-to-endothelial cell crosstalk by the cholesterol metabolite 27HC promotes atherosclerosis in male mice
Source: Nat Commun. 2023 Jul 25;14:4101. doi: 10.1038/s41467-023-39586-z (PMC10368733; doi:10.1038/s41467-023-39586-z)
Supplement: Supplementary file 11 — Source Data [file 41467_2023_39586_MOESM11_ESM.zip › Yu.MacCyp27a1.SourceBlots.Fig3 4.0609.pptx]

## Slide 1
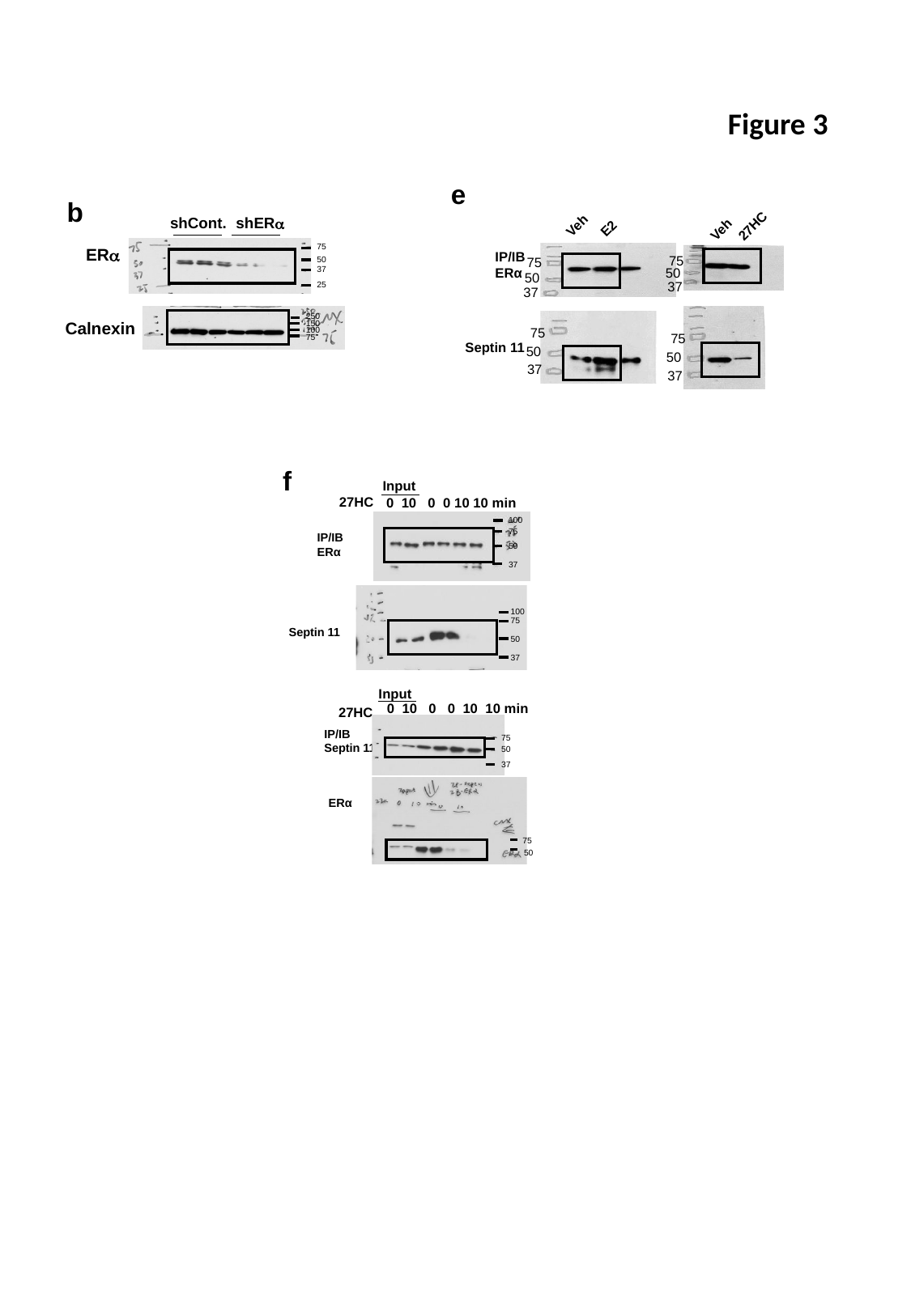

Figure 3
e
Veh
27HC
E2
Veh
IP/IB
ERα
Septin 11
b
shCont.
shERα
ERα
Calnexin
75
75
75
50
37
50
50
37
25
37
250
150
75
100
75
75
50
50
37
37
f
Input
27HC
0 10 0 0 10 10 min
100
75
IP/IB
ERα
50
37
100
75
Septin 11
50
37
Input
0 10 0 0 10 10 min
IP/IB
Septin 11
ERα
27HC
75
50
37
75
50

## Slide 2
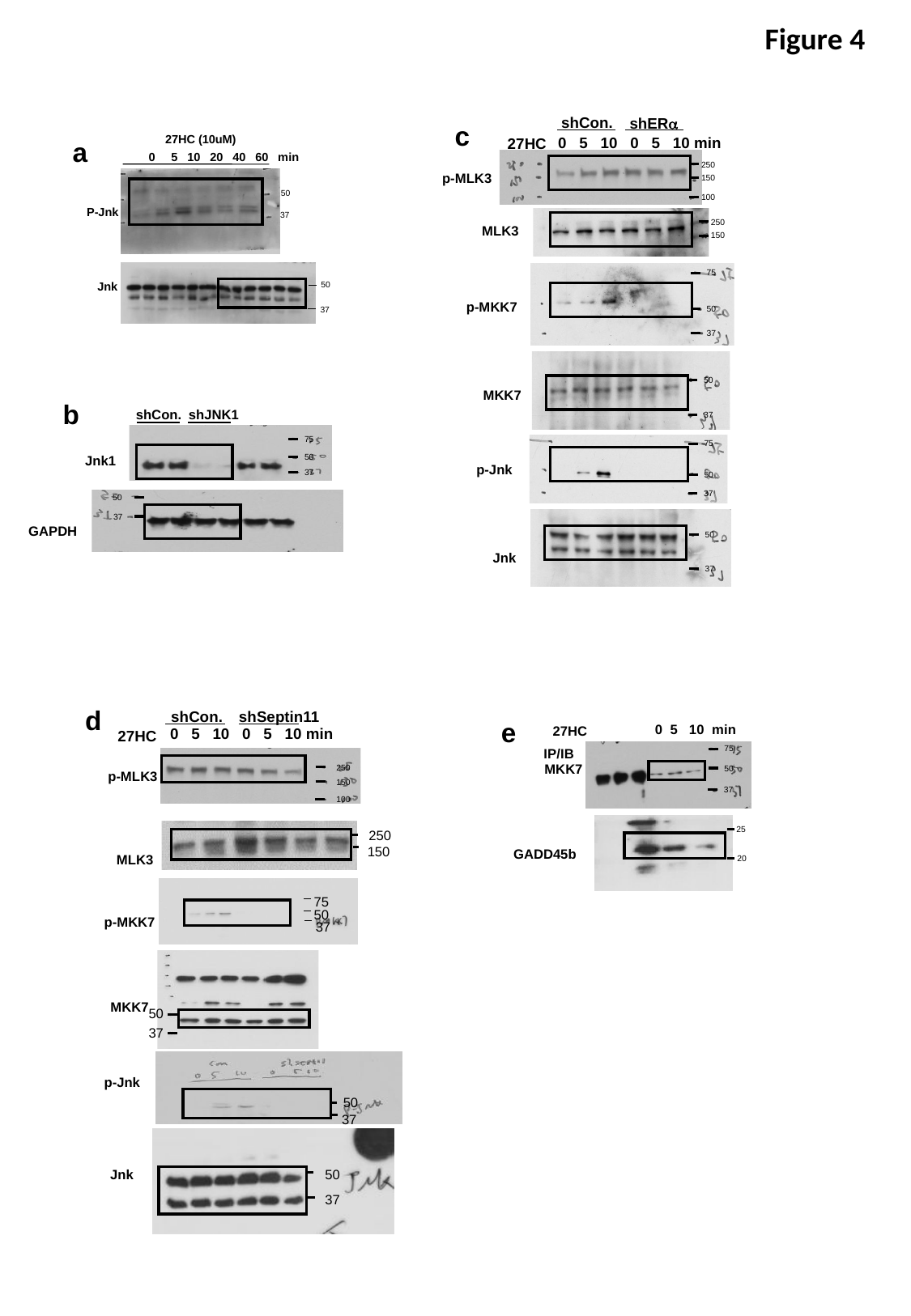

Figure 4
shCon.
shERα
c
 0 5 10 0 5 10 min
a
27HC (10uM)
0 5 10 20 40 60 min
50
37
P-Jnk
50
Jnk
37
27HC
250
p-MLK3
150
100
250
MLK3
150
75
p-MKK7
50
37
50
MKK7
b
shCon.
shJNK1
37
75
75
50
Jnk1
p-Jnk
37
50
37
50
37
GAPDH
50
Jnk
37
d
shSeptin11
shCon.
 0 5 10 0 5 10 min
27HC
p-MLK3
250
150
MLK3
75
50
37
p-MKK7
MKK7
p-Jnk
50
37
50
37
Jnk
50
37
e
0 5 10 min
27HC
IP/IB
MKK7
25
GADD45b
20
75
250
50
150
37
100

## Slide 3
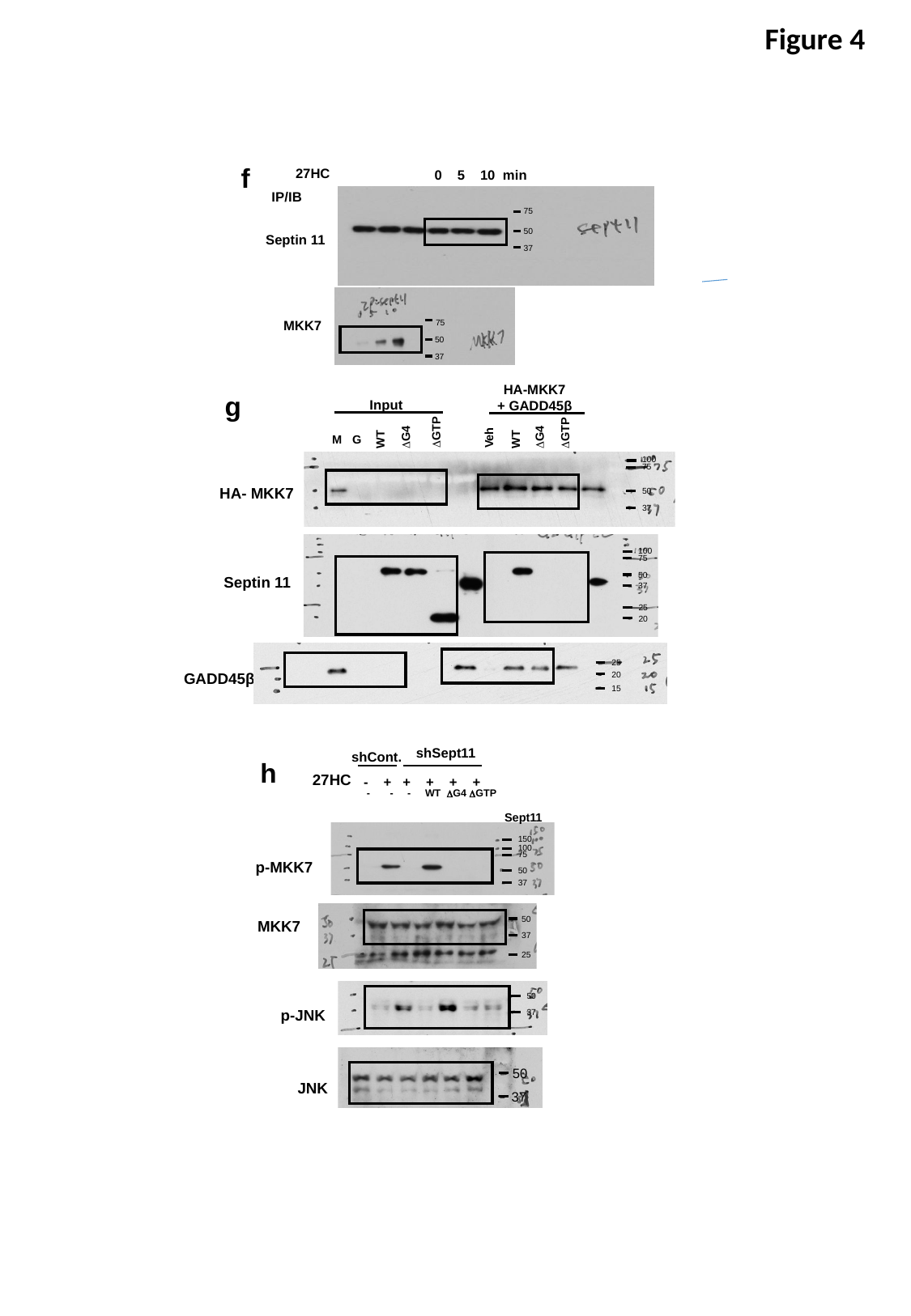

Figure 4
f
27HC
0 5 10 min
IP/IB
75
50
Septin 11
37
75
MKK7
50
37
HA-MKK7
+ GADD45β
g
Input
ΔGTP
ΔGTP
ΔG4
ΔG4
Veh
WT
M
G
WT
100
75
HA- MKK7
50
37
100
75
50
Septin 11
37
25
20
25
GADD45β
20
15
shSept11
shCont.
h
27HC
 - + + + + +
 - - - WT ΔG4 ΔGTP
Sept11
150
100
75
p-MKK7
50
37
50
MKK7
37
25
50
p-JNK
37
50
JNK
37
